# Development and validation of an early warning model for hospitalized COVID-19 patients: a multi-center retrospective cohort study

**DOI:** 10.1186/s40635-022-00465-4

**Published:** 2022-09-19

**Authors:** Jim M. Smit, Jesse H. Krijthe, Andrei N. Tintu, Henrik Endeman, Jeroen Ludikhuize, Michel E. van Genderen, Shermarke Hassan, Rachida El Moussaoui, Peter E. Westerweel, Robbert J. Goekoop, Geeke Waverijn, Tim Verheijen, Jan G. den Hollander, Mark G. J. de Boer, Diederik A. M. P. J. Gommers, Robin van der Vlies, Mark Schellings, Regina A. Carels, Cees van Nieuwkoop, Sesmu M. Arbous, Jasper van Bommel, Rachel Knevel, Yolanda B. de Rijke, Marcel J. T. Reinders

**Affiliations:** 1 Department of Intensive Care, Erasmus University Medical Center, Rotterdam, The Netherlands; 2 EEMCS, Pattern Recognition and Bio-Informatics Group, Delft University of Technology, Delft, The Netherlands; 3 Department of Clinical Chemistry, Erasmus University Medical Center, Rotterdam, The Netherlands; 4 Department of Intensive Care, Haga Teaching Hospital, The Hague, The Netherlands; 5 General Internal Medicine, Department of Internal Medicine, Amsterdam Public Health Research Institute, Amsterdam UMC, Location VU University Medical Centre, Amsterdam, The Netherlands; 6 Department of Clinical Epidemiology, Leiden University Medical Center, Leiden, The Netherlands; 7 Department of Internal Medicine, Maasstad Teaching Hospital, Rotterdam, The Netherlands; 8 Department of Internal Medicine, Albert Schweitzer Teaching Hospital, Dordrecht, The Netherlands; 9 Department of Rheumatology, Haga Teaching Hospital, The Hague, The Netherlands; 10 Team Business Intelligence, Maasstad Teaching Hospital, Rotterdam, The Netherlands; 11 Department of Rheumatology, Leiden University Medical Center, Leiden, The Netherlands; 12 Department of Infectious Diseases, Leiden University Medical Center, Leiden, The Netherlands; 13 Team Business Intelligence, Albert Schweitzer Teaching Hospital, Dordrecht, The Netherlands; 14 Department of Clinical Chemistry, MaasstadLab, Maasstad Teaching Hospital, Rotterdam, The Netherlands; 15 Department of Internal Medicine, Ikazia Teaching Hospital, Rotterdam, The Netherlands; 16 Department of Internal Medicine, Haga Teaching Hospital, The Hague, The Netherlands; 17 Department of Intensive Care, Leiden University Medical Center, Leiden, The Netherlands; 18 Translational Clinical Research Institute, Newcastle University, Newcastle, UK

**Keywords:** COVID-19, Early warning score, Intensive care, Machine learning, Artificial intelligence, Medical prediction model, Dynamic model updating

## Abstract

**Background:**

Timely identification of deteriorating COVID-19 patients is needed to guide changes in clinical management and admission to intensive care units (ICUs). There is significant concern that widely used Early warning scores (EWSs) underestimate illness severity in COVID-19 patients and therefore, we developed an early warning model specifically for COVID-19 patients.

**Methods:**

We retrospectively collected electronic medical record data to extract predictors and used these to fit a random forest model. To simulate the situation in which the model would have been developed after the first and implemented during the second COVID-19 ‘wave’ in the Netherlands, we performed a temporal validation by splitting all included patients into groups admitted before and after August 1, 2020. Furthermore, we propose a method for dynamic model updating to retain model performance over time. We evaluated model discrimination and calibration, performed a decision curve analysis, and quantified the importance of predictors using SHapley Additive exPlanations values.

**Results:**

We included 3514 COVID-19 patient admissions from six Dutch hospitals between February 2020 and May 2021, and included a total of 18 predictors for model fitting. The model showed a higher discriminative performance in terms of partial area under the receiver operating characteristic curve (0.82 [0.80–0.84]) compared to the National early warning score (0.72 [0.69–0.74]) and the Modified early warning score (0.67 [0.65–0.69]), a greater net benefit over a range of clinically relevant model thresholds, and relatively good calibration (intercept = 0.03 [− 0.09 to 0.14], slope = 0.79 [0.73–0.86]).

**Conclusions:**

This study shows the potential benefit of moving from early warning models for the general inpatient population to models for specific patient groups. Further (independent) validation of the model is needed.

**Supplementary Information:**

The online version contains supplementary material available at 10.1186/s40635-022-00465-4.

## Background

The COVID-19 pandemic has continued to put pressure on hospital care worldwide. As COVID-19 patients may deteriorate rapidly and unexpectedly, timely identification of deterioration is needed to guide changes in clinical management, e.g., admission to intensive care units (ICUs). Widely used Early warning scores (EWSs) based on aggregate-weighted vital signs have been developed for this purpose already, i.e., the Modified Early Warning Score (MEWS) [1], National Early Warning Score (NEWS) [2, 3] and its successor, NEWS2 [4]. A recent systematic review [5] showed that NEWS2 has been validated for COVID-19 patients in various studies. However till date, only one study [6] validated it for the purpose it was originally designed for, namely longitudinal monitoring to identify clinical deterioration over a 24-h interval. Moreover, these existing EWSs were designed for the general inpatient population and do not differentiate between various rates of oxygen delivery. Consequently, there is significant concern that these scores underestimate severity of illness in COVID-19 patients [7–11]. Many new prognostic models for COVID-19 have been developed [12], but most of these are intended to predict outcomes at the point of hospital admission instead of longitudinal inpatient monitoring. Moreover, most use relatively long or unspecified prediction horizons, whereas for the task of early warning, a prediction horizon limited to a few days is recommended [13].

We aimed to develop an early warning model for longitudinal monitoring of hospitalized COVID-19 patients, based on patient demographics and vital signs, and benchmark it against existing EWSs.

## Methods

The Medical Ethics Committee at Erasmus MC, Rotterdam, The Netherlands, waived the need for patient informed consent and approved an opt-out procedure for the collection of COVID-19 patient data during the COVID-19 crisis. The study is reported in accordance with the TRIPOD guidelines [14].

### Study population and data collection

The study was performed in six hospitals in the Netherlands, South Holland province, consisting of two academic hospitals and four teaching hospitals. We collected electronic medical record (EMR) data from patients admitted with COVID-19, defined as a positive real-time reverse transcriptase polymerase chain reaction (RT-PCR) assay for SARS-CoV-2 or a COVID-19 Reporting and Data System (CO-RADS) score [15] ≥ 4 and clinical suspicion without obvious other causes of respiratory distress. The periods of data collection varied per hospital and ranged between February 2020 and May 2021.

### Outcome

We used patient deterioration as a primary outcome, defined as a composite outcome of intensive care unit (ICU) admission or unexpected death on the ward, within 24 h from the moment of prediction. We qualified each patient death as unexpected unless it occurred after initiation of end-of-life care (EoLC) or a ‘do not admit to ICU’ order.

### Participants

We handled patients who returned to the same hospital for COVID-19-related matters, after being discharged first, as separate admissions. We validated the model using the observation set definition [16], i.e., we collected multiple observation sets (‘samples’) of each patient at different time points, using the most recently observed set of predictors. We collected samples starting at 8 h after hospital admission and added one every 24 h until discharge, ICU admission, or death. We labeled samples as positive if ICU admission or death occurred within 24 h from the moment of sampling, and negative otherwise. We excluded patients (1) who were admitted to the ICU straight from home or the emergency department, (2) who were hospitalized shorter than eight hours, and (3) for who EoLC or a ‘do not admit to ICU’ order was initiated somewhere during hospitalization. We censored patients who were transferred to other hospitals at the moment of transfer. For patients who were still admitted when the data were collected, we censored at 24 h before the final observed measurement, consequently excluding still admitted patients who stayed shorter than 24 h.

### Predictors

As recommended by Wynants and colleagues [12], we selected a set of candidate predictors which were identified as clinically important in COVID-19 patients in the literature (Additional file 1: Table S1). Additionally, to effectively model the degree of supplemental oxygen (O_2_) a patient required, we added O_2_ both as a binary (yes/no) and continuous (L/min) predictor. To measure O_2_ relative to the patient’s oxygenation, we added the SpO_2_ to O_2_ ratio (SpO_2_/O_2_). We added changes (∆s) in frequently measured vital signs to model their dynamics. We added the AVPU (Alert, Verbal, Pain, Unresponsive) score [17] using ordinal encoding (i.e., A = 0, V = 1, P = 2, U = 3). Finally, to correct for time dependency of some included predictors and model the effect of duration of the hospitalization on the prior deterioration risk, we added the current length-of-stay on the ward as a predictor. We excluded predictors with entry densities (i.e., fractions of non-empty daily measurements) less than 50% within the development set. More details on the definitions of the candidate predictors can be found in Additional file 1: appendix A.

### Missing data

We imputed the categorical predictors for each sample separately by fitting a logistic regression model for sex and a multinomial logistic regression model for AVPU, using sex or AVPU as outcomes and the remaining data as predictors. To impute the missing values among the continuous predictors, we used the ‘IterativeImputer’ function offered by scikit-learn in Python [18], which imputes each predictor with missing values based on the other predictors with Bayesian ridge regression in an iterated round-robin fashion (Additional file 1: appendix B).

### Model development

We fitted a random forest (RF) model to discriminate between positive and negative samples. To examine the added value of the inclusion of non-linear predictor–outcome relations by the RF model, we also fitted a logistic regression (LR) model with L2 regularization. First, we normalized the samples by centering each predictor and scaling them by the standard deviation (based on the development set). After imputation, we optimized the ‘maximum tree depth’ and ‘max features’ hyperparameters of the RF model and the regularization strength (*λ*) of the LR model using an exhaustive grid search in a stratified tenfold cross-validation procedure within the development set (optimizing the area under the receiver operating characteristic curve). Additional file 1: Table S2 shows the hyperparameter grids that were searched. Finally, we fitted the models with the optimized hyperparameters using the development set and validated them using the test set.

### Model validation

We validated the models temporally, and evaluated two model implementation strategies: a static and a dynamic strategy. First (static strategy), to simulate the situation in which the models would have been developed after the first COVID-19 ‘wave’ in the Netherlands and implemented during the second wave, we split the data for patients admitted before and after August 1, 2020, forming the development set and test set, respectively. We fitted an RF and LR model using the development set and validated these using the test set, as described in Sect. 3.6 (Additional file 1: Fig. S1). We refer to these as the ‘static’ models.

**Fig. 1:**
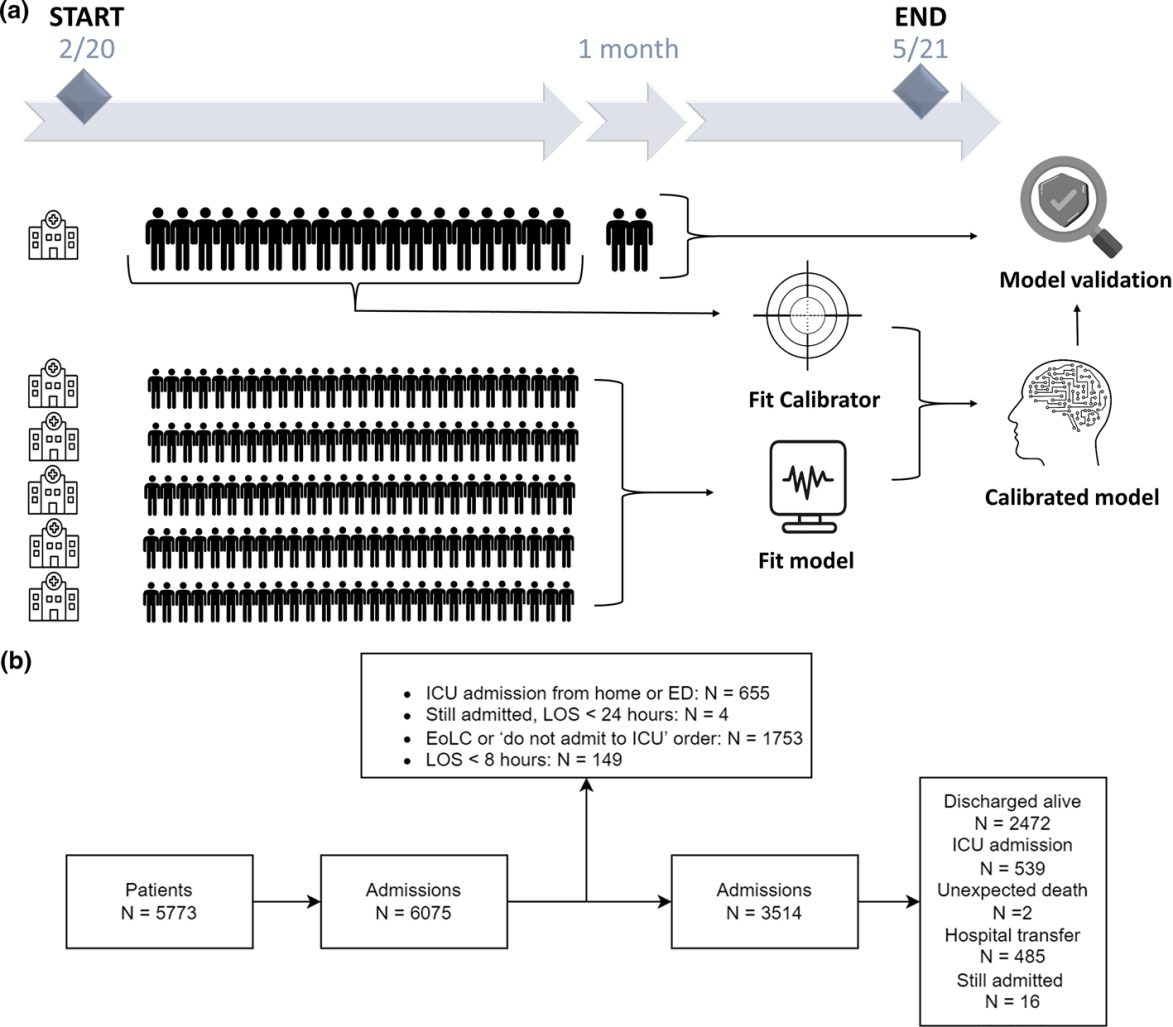
Study design. **a** Schematic representation of the dynamic model updating procedure. For example, to predict deterioration for patients admitted to hospital A in October 2020, the model is fitted using patient data collected up to that date in the remaining hospitals, and a calibrator is fitted using patient data collected up to that date in hospital A. These two combined result in calibrated predictions. This process is repeated each month, for each hospital, from August 2020 until May 2021. **b** Flowchart of patient inclusion. ICU = intensive care unit, ED = emergency department, EoLC = end-of-life care, LOS = length-of-stay

Second (dynamic strategy), as changes over time may lead to degraded model performance, we simulated the situation in which models would have been developed after the first wave, implemented, and updated each month during the second wave. Therefore, each month from August 2020 to May 2021, we updated the static models using patient data that would have been available up to that point and validated these using data of the next month (Fig. 1a). The model updating we implemented was twofold: model fitting and hospital-specific recalibration. The latter was performed with a mapping function (i.e., a calibrator), which we fitted using isotonic regression [19] and which re-maps the predictions of the fitted model (Additional file 1: appendix C). Each month, for each hospital, we fitted models using all available data up to that month of the five other hospitals and recalibrated the models using all available data up to that month of the hospital itself. Thus, to validate the model each month, models are updated solely based on data that would have been available up to that month, avoiding any leakage from the development set to the test set. We refer to these as the ‘dynamic’ models. An evaluation of other dynamic model updating strategies can be found in Additional file 1: appendix G.

Additionally, we validated an RF and LR model in a more classical way (retrospectively), using a ‘leave-one-hospital-out’ cross-validation procedure. That is, in each iteration, all patients from five hospitals across the full study period formed the development set and all patients from the remaining hospital formed the test set (Additional file 1: Fig. S2).

### Evaluation metrics

Model discrimination for medical prediction models is often quantified by the area under the receiver operating characteristic curve (AUC). In this setting, we consider a false positive rate (FPR) > 0.33 as clinically undesirable as we argue that it will lead to alert fatigue [20]. Moreover, other NEWS validation studies [21, 22] have shown that the recommended triggers (i.e., NEWS = 5 or NEWS = 7 [3, 4]) appear in the receiver operating characteristic curve in FPR ranges between 0 and 0.33. Therefore, we consider the partial AUC [23] (pAUC, Additional file 1: appendix F.2) between 0 and 0.33 FPR as our primary endpoint and the (complete) AUC as a secondary endpoint. Also, the positive predictive value (PPV) is suggested as a useful metric to evaluate the clinical usability of EWSs [16, 21, 24]. Therefore, we evaluated the area under the precision–recall curve [25] (AUCPR, Additional file 1: appendix F.3). We assessed 95% confidence intervals (CIs) to calculate uncertainties around the different metrics using bootstrap percentile confidence intervals [26] (with 1000 bootstrap replications stratified for positive and negative samples) and tested the statistical significance of the improvements in discriminative performance between models as described in Additional file 1: appendix F.4.

We performed a decision curve analysis (DCA) [27] to quantify the clinical utility of the models in terms of net benefit (NB, Additional file 1: appendix F.5). We considered early detection of a deteriorating patient as at least four times more important than preventing an unnecessary response (false alarm), and therefore we plotted the DCA results up to 0.2 deterioration probability. The NB is normalized as the fraction of the maximum NB.

Following the calibration hierarchy defined by Van Calster and colleagues [28], we evaluated model calibration in the ‘weak’ sense by calculating calibration intercepts and slopes [29] (Additional file 1: appendix F.6) and in the ‘moderate’ sense by plotting smoothed calibration curves [30].

Each metric was evaluated based on the complete test set (i.e., the overall performance) and the test subsets from the individual hospitals.

### Explainable predictions

To obtain interpretability for the developed models, we calculated the impact of individual predictors on risk output by SHapley Additive exPlanations (SHAP) values. A SHAP value is a model-agnostic representation of predictor importance, where the impact of each predictor is represented using Shapley values inspired by cooperative game theory [31]. We calculated SHAP values based on RF and LR models fitted on the complete dataset.

### Comparison with existing early warning scores

To benchmark the models against existing EWSs, we calculated the MEWS [1] and the NEWS [2] for each sample. We validated both scores in the same fashion (using the same imputation) as the RF and LR models. For the DCA, we transformed the discrete scores into probabilities by fitting two calibrators based on the development data using isotonic regression, with, respectively, the MEWS or the NEWS as the only predictor.

### Additional experiments

To compare the RF model with another non-linear model, we repeated the temporal validation with a Gradient Boosting (XGBoost) model, optimizing the hyperparameters described in Additional file 1: Table S2. To examine the added value of predictive modeling compared to aggregate-weighted scores, we compared the performance of the MEWS and NEWS in the temporal validation with an extra RF and LR model fitted only with the predictors required to calculate the MEWS (i.e., heart rate, respiratory rate, systolic blood pressure, temperature, AVPU) and NEWS (which adds supplemental *O*_2_ (yes/no) and *SpO*_2_). To further examine the influence of the included predictors on the model performance, we fitted extra models using more (i.e., by allowing more missingness) and fewer (i.e., by selecting on importance) predictors. Finally, we examined the influence of the imputation strategy on the model performance by repeating the temporal validation for 50 unique imputation rounds.

## Results

### Cohort description

We included 3514 COVID-19 patient admissions in six Dutch hospitals within varying time windows ranging between February 2020 until May 2021 (Additional file 1: Fig. S3). Table 1 shows the pathway and population characteristics for all included admissions. Pathway and population characteristics separately for admissions before and after August 1 can be found in Additional file 1: Tables S5 and S6 and for the individual hospitals in Additional file 1: Tables S7–S12. ICU admission occurred in 539 (15.3%), unexpected death in two (< 0.1%), and hospital transfer in 485 (13.8%) admissions (Fig. 1b). Additional file 1: Table S3 shows the occurrence of different patient outcomes across the different hospitals. Occurrence of ICU admission over the whole study period was notably low in hospitals B and C (11.4% and 10.7%) compared to the other hospitals (ranging between 16.3% and 19.4%). We identified 47 candidate predictors of which, after exclusion due to missingness, 18 were included (Additional file 1: Table S1). To examine the role of included predictors in relation to the outcome, we plotted cumulative predictor distributions for positive and negative samples (Additional file 1: Fig. S4). Here, *O*_2_, *SpO*_2_/*O*_2_, respiratory rate and temperature show notable differences between the positive and negative sample distributions. We plotted the correlations between the predictors (before imputation) in a clustered heatmap (Additional file 1: Fig. S5). The vital signs with their dynamic counterparts (∆s) showed strong positive correlations, as well as temperature with heart rate. *O*_2_ and respiratory rate showed strong negative correlations with *SpO*_2_/*O*_2_.

**Table 1:** Pathway and population characteristics

	DA (*N* = 2472)	ICU (*N* = 539)	Died (*N* = 2)	Transfer (*N* = 485)	SA (*N* = 16)	Total (*N* = 3514)
Sex male, %	55.3	64.7	100.0	56.9	56.2	57.0
Female, %	43.1	34.9	0.0	39.8	43.8	41.3
Unknown, %	1.6	0.4	0.0	3.3	0.0	1.6
Age, years med (IQR)	61.0 (51.0–70.0)	63.0 (55.0–70.0)	76.0 (74.5–77.5)	60.0 (53.2–69.0)	66.5 (55.0–75.5)	61.0 (52.0–70.0)
Mean (SD)	59.6 (14.2)	61.5 (11.7)	76.0 (3.0)	59.8 (11.9)	64.5 (11.6)	60.0 (13.5)
Ward LOS, days med (IQR)	3.7 (1.9–6.4)	2.3 (1.1–3.9)	7.6 (7.2–7.9)	1.1 (0.8–2.0)	4.7 (1.2–14.5)	2.9 (1.5–5.5)
Mean (SD)	5.3 (6.8)	3.4 (4.4)	7.6 (0.7)	1.8 (2.3)	8.6 (8.9)	4.5 (6.2)
RR, breaths/min med (IQR)	18.0 (16.0–22.0)	22.0 (19.0–26.0)	20.0 (20.0–20.0)	20.0 (16.8–24.0)	18.0 (16.0–23.5)	20.0 (16.0–24.0)
Mean (SD)	19.3 (5.0)	22.9 (6.0)	20.0 (0.0)	20.8 (5.1)	20.7 (6.2)	20.1 (5.4)
SpO_2_, % med (IQR)	96.0 (95.0–98.0)	95.0 (94.0–97.0)	95.5 (95.2–95.8)	95.0 (94.0–97.0)	95.0 (93.5–97.0)	96.0 (94.2–97.0)
Mean (SD)	96.0 (3.5)	95.1 (4.8)	95.5 (0.5)	95.5 (2.2)	94.5 (3.4)	95.8 (3.6)
SBP, mmHg med (IQR)	125.0 (113.0–137.0)	125.0 (114.0–137.0)	137.5 (136.8–138.2)	123.0 (113.0–133.5)	119.0 (107.2–126.8)	124.0 (113.0–136.0)
Mean (SD)	126.4 (18.8)	127.4 (19.3)	137.5 (1.5)	124.1 (16.5)	120.0 (17.3)	126.2 (18.6)
T, °C med (IQR)	37.1 (36.6–37.8)	37.3 (36.7–38.0)	37.0 (37.0–37.1)	37.0 (36.6–37.7)	36.8 (36.2–37.0)	37.1 (36.6–37.8)
Mean (SD)	37.2 (0.9)	37.4 (1.0)	37.0 (0.1)	37.2 (0.9)	36.8 (0.7)	37.2 (0.9)
HR, bpm med (IQR)	81.0 (71.0–91.0)	83.0 (73.0–92.0)	91.0 (84.0–98.0)	81.0 (72.0–90.0)	80.0 (73.8–84.8)	81.0 (72.0–91.0)
Mean (SD)	82.0 (15.2)	83.5 (15.2)	91.0 (14.0)	81.6 (13.7)	81.2 (13.6)	82.2 (15.0)
O_2_, yes/no, %	57.4	76.4	0.0	82.5	68.8	63.8
O_2_, L/min med (IQR)	3.0 (2.0–4.0)	6.0 (3.0–12.0)	–	4.0 (2.0–5.0)	3.0 (2.0–7.5)	3.0 (2.0–5.0)
Mean (SD)	3.6 (3.0)	7.5 (5.4)	–	4.4 (3.0)	5.5 (5.1)	4.5 (3.9)
SpO_2_/O_2_, 1/(L/min) med (IQR)	32.7 (23.5–48.5)	15.8 (8.1–31.0)	–	24.2 (18.6–47.0)	30.7 (14.1–48.5)	31.7 (18.6–48.0)
Mean (SD)	41.4 (26.5)	23.0 (21.3)	–	32.4 (21.1)	35.0 (24.1)	36.4 (25.7)

*DA* discharged alive, *ICU* intensive care unit, *SA* still admitted, *IQR* interquartile range, *SD* standard deviation, *LOS* length-of-stay, *RR* respiratory rate, *SBP* systolic blood pressure, *T* temperature, *HR* heart rate

### Model discrimination

**Fig. 2:**
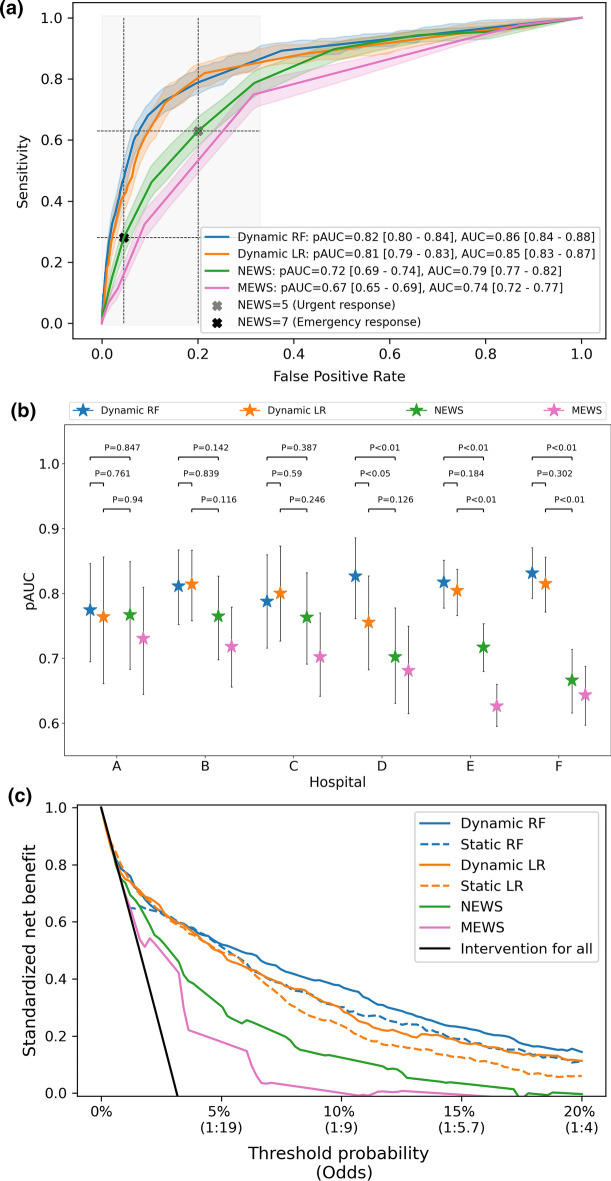
Model discrimination and decision curve analysis. **a** Overall ROC curves for the RF and LR models and the NEWS. We placed two landmarks for a NEW score of 5 and 7, i.e., the recommended trigger thresholds for an urgent and emergency response. We calculated both the pAUC between a false positive rate of 0 and 0.33 (grey area) and the complete AUC. Shaded areas around each point in the ROC curves represent the 95% bootstrap percentile CIs^25^ (with 1000 bootstrap replications stratified for positive and negative samples). **b** Hospital-specific pAUCs. The error bars represent the 95% bootstrap percentile CIs^25^ (with 1000 bootstrap replications stratified for positive and negative samples). *P*-values, calculated as described in Additional file 1: appendix F.4, are shown for the difference in pAUC between the RF models and NEWS (upper bar), between the RF and LR models (middle bar) and between the LR models and NEWS (lower bar). **c** Overall decision curve analysis results. The standardized net benefit is plotted over a range of clinically relevant probability thresholds with corresponding odds. The ‘Intervention for all’ line indicates the NB if a (urgent or emergency) response would always be triggered

We simulated the situation in which models would have been developed after the first wave, implemented, and updated each month during the second wave, i.e., the dynamic models. The overall receiver operating characteristic (ROC) curves (and corresponding pAUCs and AUCs) yielded by these models and the existing EWSs are depicted in Fig. 2a. The dynamic RF model outperformed the NEWS and the MEWS in terms of pAUC with, respectively, 10 and 15 percentage points. We placed landmarks in the ROC curve of the NEWS that correspond with the recommended triggers for an urgent and emergency response [3]. An emergency response triggers a critical care outreach team to respond quickly. Vertical differences between the ROC curves represent the potential improvement in the early detection of deteriorating COVID-19 patients. The horizontal differences represent the potential reduction in false alarms. Also in terms of AUC and AUCPR, the dynamic models outperformed the existing EWSs (Additional file 1: Table S4). We also simulated the situation in which models would have been implemented after the first wave without any updating, i.e., the static models. These yielded very similar discriminative performance compared to the dynamic models, with a pAUC of 0.81 [0.79–0.83] and 0.80 [0.78–0.82], respectively, for the RF and LR model. Finally, we validated the model retrospectively, for which the results are summarized in Additional file 1: appendix H.

Figure 2b shows the pAUCs yielded by the dynamic models and the existing EWSs in the individual hospitals. The dynamic models outperformed the existing EWSs in most of the hospitals. The static models yielded similar results (Additional file 1: Fig. S6). Hospital-specific results in terms of AUCPR and AUC are depicted in Additional file 1: Fig. S7.

### Decision curve analysis

Figure 2c shows the results of the decision curve analysis (DCA). Both static and dynamic models show a clear improvement in net benefit (NB) compared to the existing EWSs. Both dynamic models yielded higher NBs compared to the static models and the RF models yielded higher NBs compared to the LR models. Also in most of the individual hospitals, the dynamic models show improved NBs compared to the existing EWSs (Additional file 1: Fig. S8).

### Model calibration

**Fig. 3:**
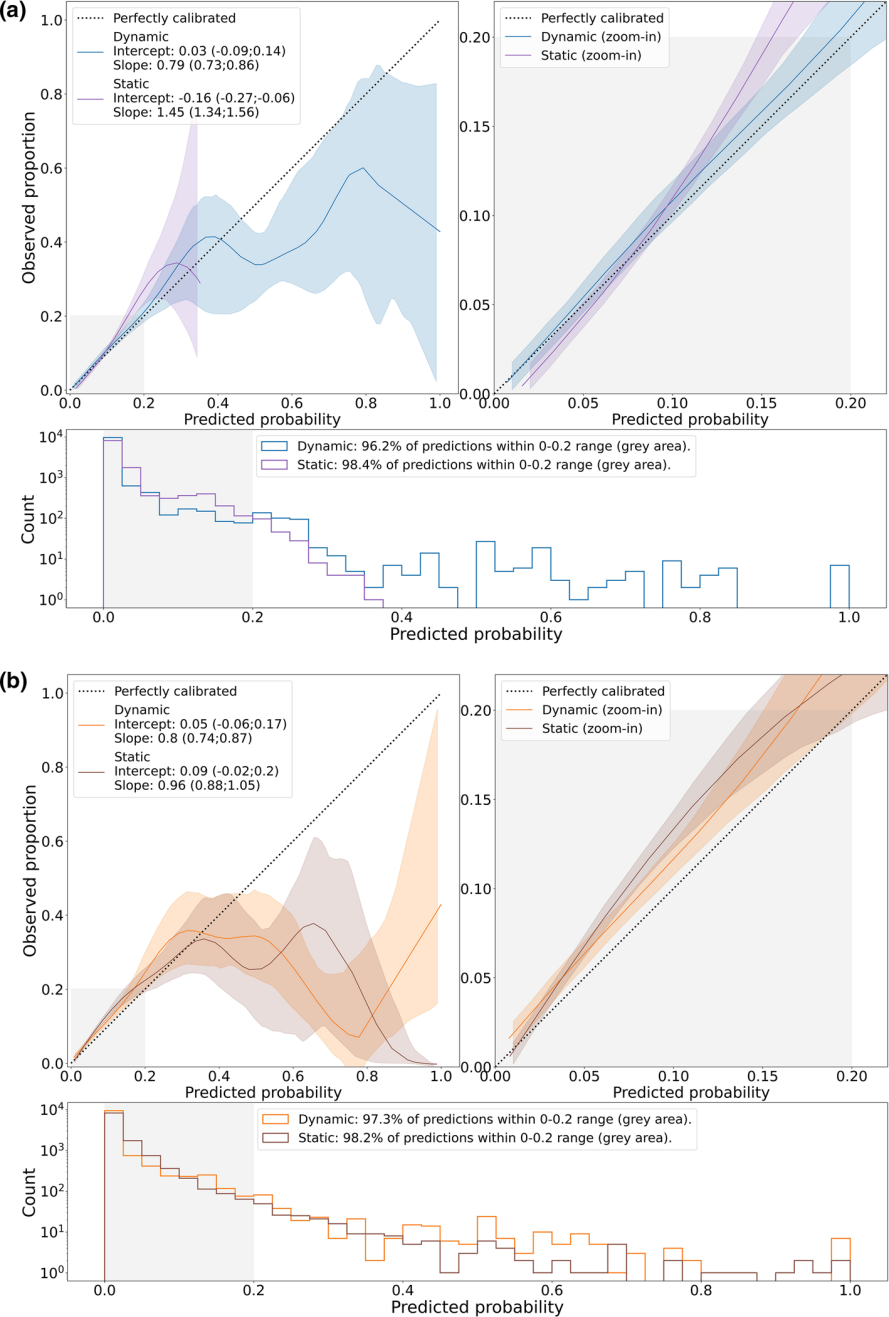
Overall model calibration of the static and dynamic RF models (**a**) and LR models (**b**). Top left: smoothed flexible calibration curves. Top right: zoom-in of the calibration curve in the 0–0.2 probability range (grey area). Shaded areas around the curves represent the 95% CIs. Bottom: histogram of the predictions (logscale). Shaded areas around each point in the calibration curves (before smoothing) represent the 95% bootstrap percentile CIs^25^ (with 1000 bootstrap replications stratified for positive and negative samples). The smooth curves including CIs were estimated by locally weighted scatterplot smoothing (see https://github.com/jimmsmit/COVID-19_EWS for the implementation). **a** Overall model calibration of the static and dynamic RF models. **b** Overall model calibration of the static and dynamic LR models

The overall calibration curves for the RF and LR models are shown in Fig. 3a and b, respectively, including the corresponding calibration intercepts and slopes. The dynamic models show improved calibration curves compared to the static models. The dynamic RF model yielded a slightly better calibration curve than the dynamic LR model. The vast majority of the static and dynamic LR and RF predictions occur in the lower probability range (i.e., 0–0.2), and therefore a good model calibration is most important in this region. The relatively small number of predictions in the higher probability range (i.e., 0.2–1) causes high uncertainty, making calibration in this region hard to judge. Hospital-specific calibration curves can be found in Additional file 1: Fig. S9, with calibration intercepts ranging from −0.37 to 0.49 and − 0.24 to 0.38 and calibration slopes ranging from 0.55 to 1.11 and 0.69 to 1.42, respectively, for the dynamic RF and LR models.

### Predictor importance

**Fig. 4:**
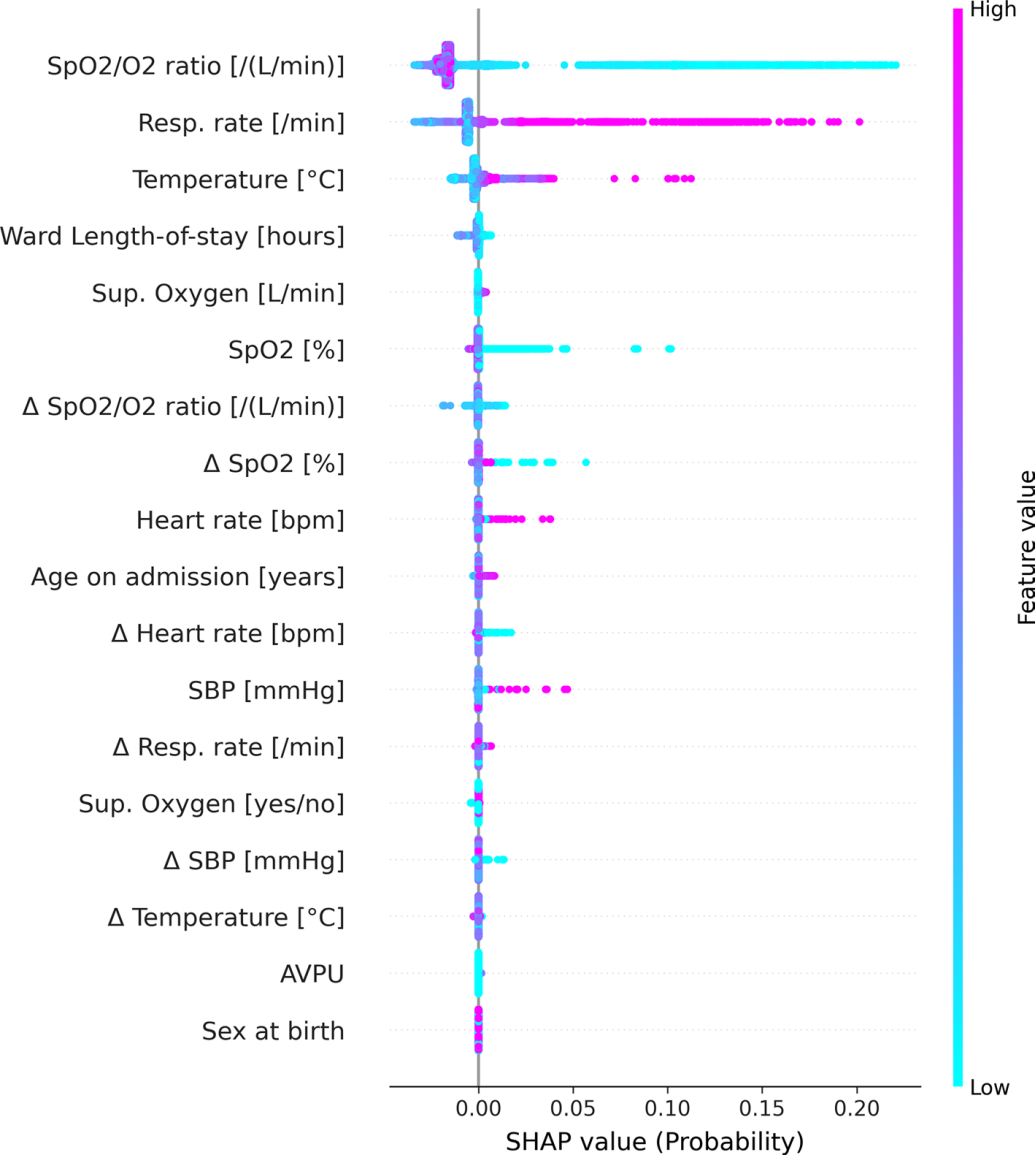
Distribution of SHapley Additive exPlanations (SHAP) values of the included predictors (based on mean SHAP magnitude) for the random forest model. For each predictor, each dot represents the impact of that predictor for a single prediction. The colors of the dots correspond with the value for the specific predictor. Thus, pink dots with positive SHAP values indicate that high values of the predictor are associated with a high risk of clinical deterioration. Conversely, blue dots with positive SHAP values indicate that low values of the predictor are associated with a high risk of clinical deterioration

The distribution of SHAP values of the included predictors is shown in Fig. 4. The top five ranked predictors were *SpO*_2_/*O*_2_, respiratory rate, temperature, ward length-of-stay and *O*_2_ (L/min). The distribution of SHAP values of the LR model shows similar ranking of importance and is shown, together with fitted model parameters, in Additional file 1: Fig. S10.

### Additional experiments

The static XGBoost model showed similar discriminative performance and similar calibration curves compared to the static RF model, but the dynamic XGBoost model showed lower pAUC and a worse calibration curve compared to the dynamic RF model (see Additional file 1: Table S4 and Fig. S11). The LR and RF models fitted with the MEWS predictors outperformed the MEWS, whereas the models fitted with the NEWS predictors yielded similar discriminative performance as the NEWS (Additional file 1: appendix I.1). The models fitted with only the top five most important predictors, as well as model fitted with the predictors required to calculate the NEWS supplemented with *SpO*_2_/*O*_2_, yielded similar performance in discrimination and calibration compared to the models fitted with the 18 originally included predictors. Models fitted with more predictors (allowing predictors with more missingness) resulted in similar discrimination, but slightly worse calibration (Additional file 1: appendix I.2). Finally, model discrimination and calibration showed little variation over 50 imputation rounds (Additional file 1: appendix I.3).

## Discussion

### Principal findings

We introduced COVID-19-specific early warning models which showed improved performance compared to the existing Early warning scores. The implementation of dynamic model updating showed to be effective to retain good model calibration over time. When implemented, this model has the potential to improve early detection of deteriorating COVID-19 patients and save workload for healthcare workers by reducing the number of false alarms, although an interventional study is required to prove this.

### Clinical implementation

The probability for clinical deterioration within 24 h predicted by the model enables clinicians to assess deterioration risk in COVID-19 patients and respond appropriately. As we present a machine learning (ML) model based on 18 predictors, pragmatically, it cannot be calculated by hand (in contrast to aggregate-weighted EWSs), but requires automated calculation using data stored in the EMR. Hence, the use of the presented model is limited to hospitals that use an EMR, which underwrites the importance of EMRs to bring machine learning models to the bedside.

### Related studies

The choice for clinical outcomes varies significantly in early warning literature [24]. The NEWS was originally intended to identify patients at risk of early cardiac arrest, unplanned ICU admission, and death [2]. As cardiac arrest unavoidably leads to either death or ICU admission, we did not handle cardiac arrest as a separate outcome but recognized these as either unplanned ICU admissions or unexpected deaths. Baker and colleagues [6], who validated NEWS2 longitudinally in COVID-19 patients, included the initiation of different non-invasive respiratory support methods (CPAP, BiPAP and HFNC) as an outcome too. In the Netherlands, these support methods are also offered to COVID-19 patients outside the ICU and therefore, we did not include these as outcomes. Also, Baker and colleagues included the initiation of EoLC as an outcome. In the Netherlands, EoLC may be initiated before a patient actually deteriorates based on, for instance, high age or significant comorbidities. Hence, a patient for whom EoLC is initiated may have a very different clinical presentation compared to a patient with full care just before ICU admission or unexpected death. Therefore, including the initiation of EoLC as an outcome may decrease the model’s predictive performance for ICU admission or unexpected death. We excluded patients where EoLC was initiated and those with a ‘do not admit to ICU’ order. In a recent NEWS validation study [32], Haegdorens and colleagues also excluded patients with a ‘do not perform cardiopulmonary resuscitation’ order. However, because these patients may still be admitted to the ICU, we did not exclude them. The ISARIC-4C group published prediction models for mortality [33] and deterioration [34] among COVID-19 patients. The key difference with the model we present is that the ISARIC-4C models provide a single prediction of adverse outcomes (based on data from hospitalization day 1), instead of longitudinal patient monitoring. We were unable to evaluate their predictive performance as they are based on predictors which were frequently missing or completely unavailable in our datasets (i.e., computed tomography findings, uncommonly measured biomarkers, or information on comorbidities).

### Model generalizability

Different factors could have influenced the generalizability of the model we presented. For instance, improved patient outcomes after the introduction of dexamethasone after the RECOVERY trial [35] halfway July, 2020, could have caused the models developed before August 2020 to overestimate deterioration when implemented afterwards. Furthermore, given the low prevalence of unexpected death (< 0.1%), the model we presented is predominantly predicting ICU admission, for which there is no universal guideline. In fact, whether a patient is admitted to the ICU depends on many factors, such as the clinician who judges the patient, the availability of beds, or the effectiveness of urgent or emergency responses triggered by EWS systems already in place. Therefore, our model is fitted for a context in which a certain policy around ICU admission exists. Hence, if these policies vary among hospitals or over time, the model may fail to generalize. This could be an explanation for the overestimation observed in hospital C, which follows a relatively conservative ICU admission policy due to the absence of medium or high care units. Also, the static RF model shows a typical sigmoid shape. Due to ‘bagging’, RF models have difficulty making predictions near 0 and 1 [19]. This could explain the overestimation in the 0–0.1 probability range and underestimation in higher ranges (i.e., 0.2–0.4). All in all, our empirical results (Fig. 3a) show that the proposed model updating strategy could correct for these factors effectively as the dynamic RF model shows relatively good calibration in future patients and among different hospitals.

We split the data for patients admitted before and after August, 2020, roughly representing hospitalized patients during the first and second COVID-19 ‘waves’ in the Netherlands. The first and second waves in the Netherlands were predominantly caused by the original (B.1.1) and the ‘alpha’ variant (B.1.1.7), respectively [36]. Thus, our results suggest that the presented (dynamic) model would generalize well for some COVID-19 variants. However, we do not know if the model would generalize well for newer variants.

### Model threshold

A frequent misunderstanding is that one should use a DCA to choose the optimal model threshold. Instead, it is more sensible to choose a clinically reasonable range of threshold probabilities, and use the DCA to compare net benefit in this range with alternative models [27]. In the context of this study, we prefer to weigh the relative harms of avoiding an urgent or emergency response for a deteriorating patient (i.e., false negative) versus unnecessary responses to non-deteriorating patients (i.e., false alarms). We chose to weigh the relative harm of a false negative at least four times higher compared to a false alarm, and therefore evaluated model thresholds up to 20%. This reasoning requires a well calibrated model (see Additional file 1: appendix F.5).

### Strengths and limitations

Our study is one of the first studies [6] to validate the NEWS for the purpose for which it was originally designed (i.e., longitudinal monitoring to identify clinical deterioration). Moreover, compared to other developed COVID-19 prediction models [37, 38] with similar endpoints (i.e., deterioration within 24 h), our dataset is relatively large, including over 3500 patients among six different hospitals. We examined the generalizability of the proposed COVID-19 early warning model thoroughly through external validation in multiple hospitals and temporally during multiple phases of the COVID-19 pandemic. We demonstrate the importance of model updating when implementing a machine learning model in practice, especially during rapidly changing situations such as the COVID-19 pandemic. However, the optimal strategy for dynamic model updating remains an open problem. The different strategies we examined (Additional file 1: appendix G) do offer some insight into the effectiveness of different strategies. Moreover, the improved performance relative to the NEWS is explained by the introduction of *SpO*_2_/*O*_2_ as a predictor (Additional file 1: appendix I.2), which models the need for oxygen quantitatively instead of only qualitatively (like in the NEWS). Hence, we expect supplementing existing EWSs (e.g., NEWS-2) with this variable would improve predictive performance in COVID-19 patients, and potentially for patients with other respiratory diseases as well.

This study has limitations. Some patients may not have experienced a serious event due to a prompt medical review followed by an intervention. Hence, we may have labeled some samples as negative, whereas a patient actually showed signs of deterioration. Furthermore, patients who are transferred to another hospital are (at the moment of transfer) typically less likely to deteriorate. Therefore, informative censoring may have introduced a bias. We treated repeated observations from individual patients as independent samples, which may have led to underestimation of the uncertainties around the model performance metrics. We neither validated the NEWS2 [4] nor the alternative version of the MEWS [39] that is mostly used in the Netherlands, as we did not have the necessary data. Data were missing for some of the predictors, which we addressed using data imputation. We used an iterative imputation method based on Bayesian ridge regression. This method assumes normally distributed predictors and may have introduced bias when missingness not at random was present [40]. While our study contains a large set of demographical and physiological measurements, several potentially relevant predictors (such as multimorbidity, frailty or pre-pandemic cognitive function [41–43]) were not available in our dataset. Such predictors could potentially improve the performance of predictive models. On the other hand, the use of only commonly available predictors (i.e., demographics and vital signs) in our model increases its clinical applicability. Finally, validation is ideally performed by independent researchers. We have therefore made the model available online and we strongly encourage others to perform further external and temporal validation.

## Conclusions

In conclusion, we have shown that a COVID-19-specific early warning model for longitudinal monitoring to identify clinical deterioration shows improved discrimination and net benefit compared to existing EWSs. We advocate further study and development of such patient group-specific EWSs as well as their evaluation in clinical practice.

## Supplementary Information

**Additional file 1.** Appendices.

## Data Availability

The data that support the findings of this study of each hospital were available for the authors affiliated to the corresponding hospital. All data that support the findings of this study were available for the first author in a pseudonymized form. The random forest model fitted on the complete dataset, as well as the code and other dependencies (i.e., for data normalization and imputation) required to perform further independent validation is available online at: https://github.com/jimmsmit/COVID-19_EWS.

## Abbreviations

EWS: Early warning score
NEWS: National early warning score
MEWS: Modified early warning score
SHAP: SHapley Additive exPlanations
TRIPOD: Transparent reporting of a multivariable prediction model for individual prognosis or diagnosis
EMR: Electronic medical record
RT-PCR: Real-time polymerase chain reaction
CO-RADS: COVID-19 reporting and data system
ICU: Intensive care unit
EoLC: End-of-life care
AVPU: Alert verbal pain unresponsive
RF: Random forest
LR: Logistic regression
AUC: Area under the receiver operating characteristic curve
DCA: Decision curve analysis
NB: Net benefit
AUCPR: Area under the precision–recall curve
