# Supplementary material for: Development and validation of an early warning model for hospitalized COVID-19 patients: a multi-center retrospective cohort study
Source: Intensive Care Med Exp. 2022 Sep 19;10:38. doi: 10.1186/s40635-022-00465-4 (PMC9482891; doi:10.1186/s40635-022-00465-4)
Supplement: Supplementary file 1 — Additional file 1. Appendices. [file 40635_2022_465_MOESM1_ESM.pdf]

Supplementary material

Development and validation of an early warning model for hospitalized COVID-19 patients: A multi-center retrospective cohort study

Appendix A: Predictor selection

We started with a set of candidate predictors identified by Knight and colleagues[1] as clinically important in COVID-19 cohorts (table 1). To effectively model the degree of supplemental oxygen (O_2) a patient required, we added the need for O_2 as a dichotomous predictor (assuming 'yes' if any O_2 measurement was observed in the preceding eight hours of the moment of sampling) and as a continuous predictor (L/min) to this set. Additionally, we added the SpO_2 to O_2 ratio (SpO_2/O_2), constructed from the most recent pair of simultaneously measured values for SpO_2 and O_2. Based on other findings in recent COVID-19 literature, we added 8 extra laboratory measures as candidate predictors. To correct for time dependency of some included predictors and model the effect of duration of the hospitalization on the prior deterioration risk, we added the current length of stay on the ward as a predictor. Finally, to model the dynamics in frequently measured vital signs, we added the signed difference (before normalization) between the first and second most recently measured value within the last 24 hours for frequently measured predictors (SpO_2, heart rate, systolic blood pressure, respiratory rate, temperature and SpO_2/O_2). If fewer than two values were available in the last 24 hours, we used the imputation strategy described in the next section. Table 1 shows the complete list of candidate predictors.

Appendix B: Missing data

We imputed the two categorical predictors, i.e. sex and AVPU (Alert, Verbal, Pain, Unresponsive),[2] by fitting logistic regression models (L2 regularization, $\lambda = 1$) with sex or AVPU as outcomes and using the remaining data as predictors (using median imputation as an initial imputation strategy). To impute the missing values among the remaining (continuous) predictors, we used the 'IterativeImputer' function offered by scikit-learn in Python.[3] This algorithm is inspired by Multivariate Imputation by Chained Equations (MICE),[4] a widely used multiple imputation strategy, but returns a single imputation instead of multiple imputations. Just as in MICE, the IterativeImputer imputes each predictor with missing values in an iterated round-robin fashion (from the predictor with the fewest missing values to most). That is, at each step, a Bayesian ridge regressor is fitted to estimate a posterior distribution for one of the predictors, based on the remaining predictors (in which missing values are imputed with median imputation as the initial imputation strategy). The missing values are then imputed by the value with the highest probability density in the posterior distribution. This is done for each predictor in an iterative fashion, and the whole process of imputing each predictor is repeated for ten rounds.

Appendix C: Isotonic regression

A commonly used method to fit a calibrator is isotonic regression (IR).[5] In IR, it is assumed that the originally fitted prediction model ranks observations correctly, leading to a non-decreasing mapping of the original predictions to the observed probabilities. The relationship between the initial model predictions (p_i) the true labels (y_i) is described as follows:

$$y_i = m(p_i) + \epsilon_i \tag{1}$$

This mapping can be fitted using IR. Given a dataset (p_i, y_i), one should find the isotonic function m such that:

$$m = argmin_z \sum (y_i - z(p_i))^2 \tag{2}$$

A stepwise constant function for z can be found by applying the pair-adjacent violators (PAV) algorithm.[5] To implement IR, we used the 'CalibratedClassifierCV' (with the cv='prefit' option) function offered by scikit-learn on Python.[3] When implementing IR, one needs to assign a part of the training set to the 'calibration set' (i.e. the data used to fit the calibrator).

Appendix D: Supplementary figures

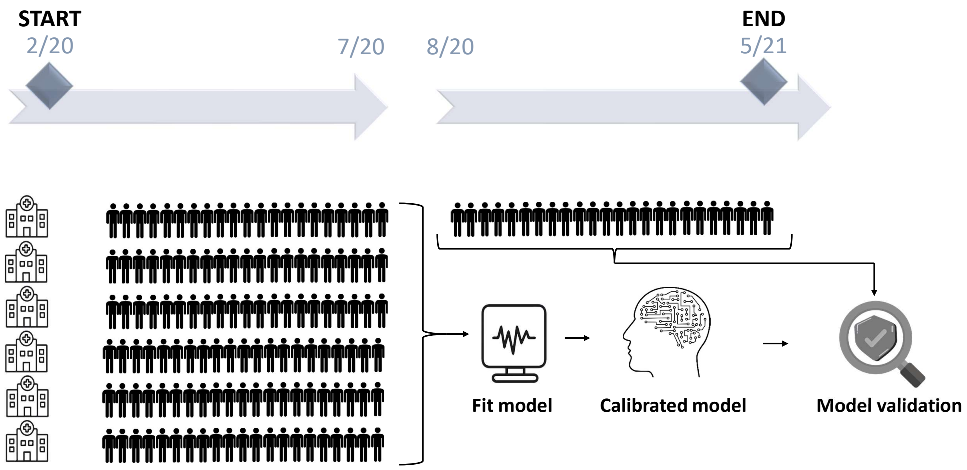

Figure 1: Schematic representation of the static models. The model is fitted on data of patients admitted before August 1, 2020, and validated for all patients admitted after this date.

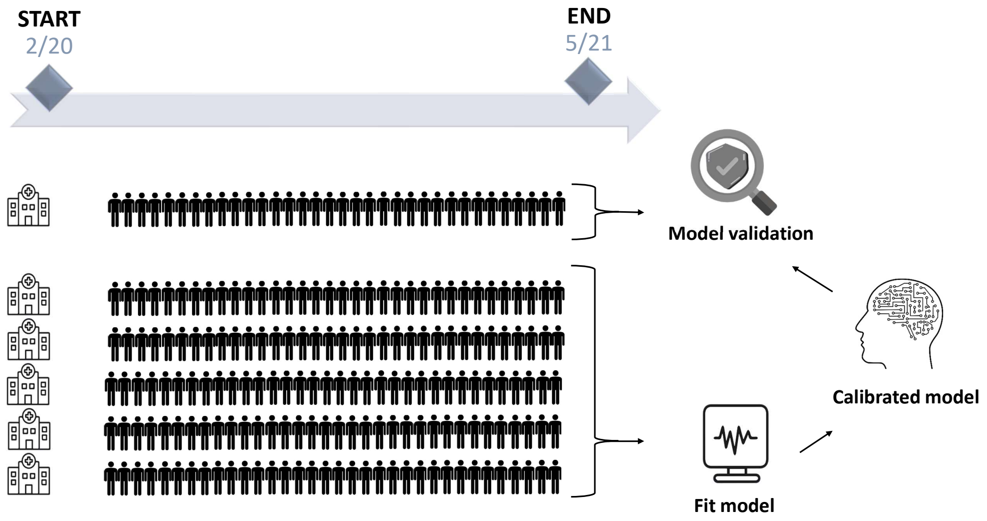

Figure 2: Schematic representation of the retrospective 'leave-one-hospital-out' validation procedure.

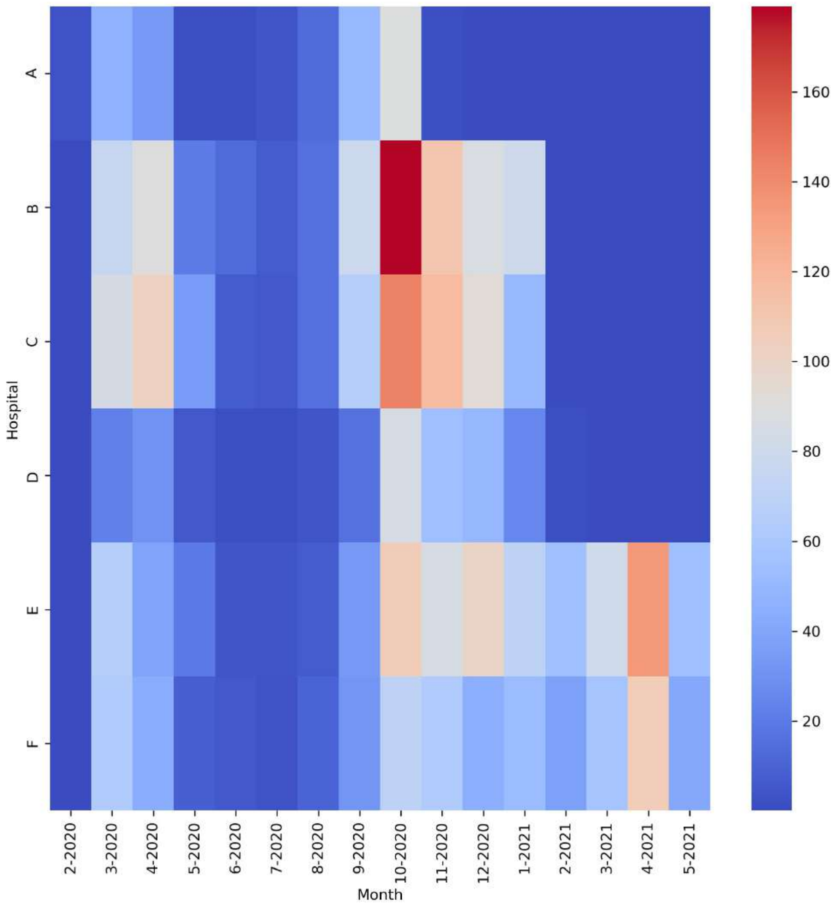

Figure 3: Heatmap describing the number of admissions per month for the different hospitals.

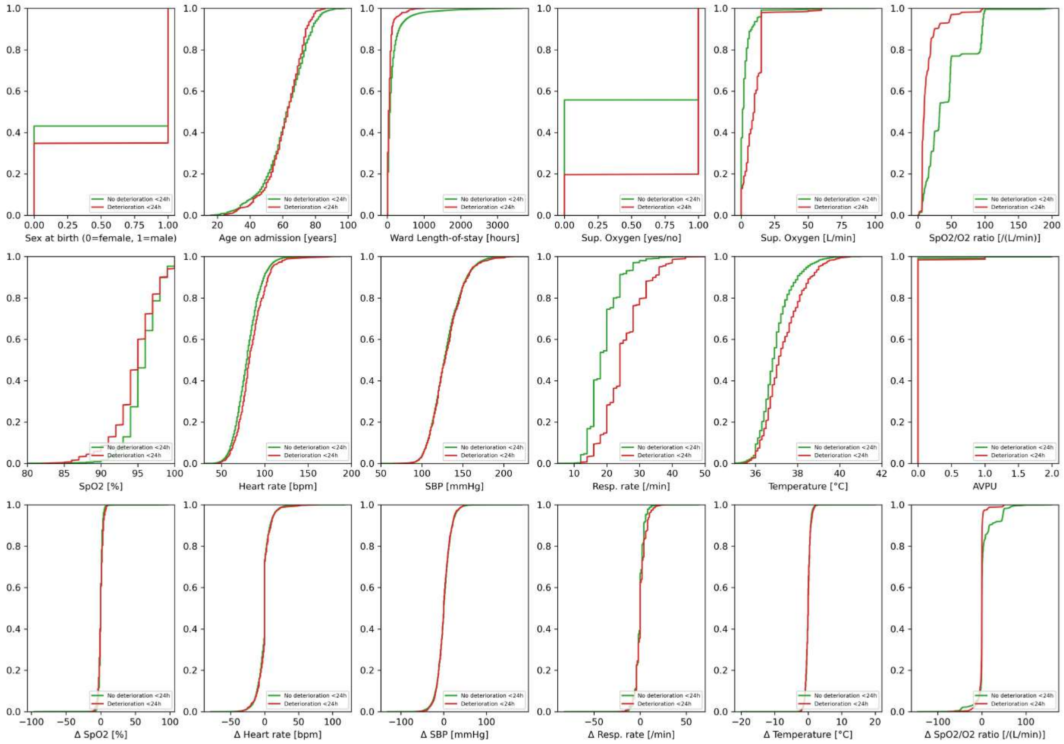

Figure 4: Cumulative distributions for the included predictors of positive (Deterioration <24 hours) and negative (No deterioration <24 hours) samples.

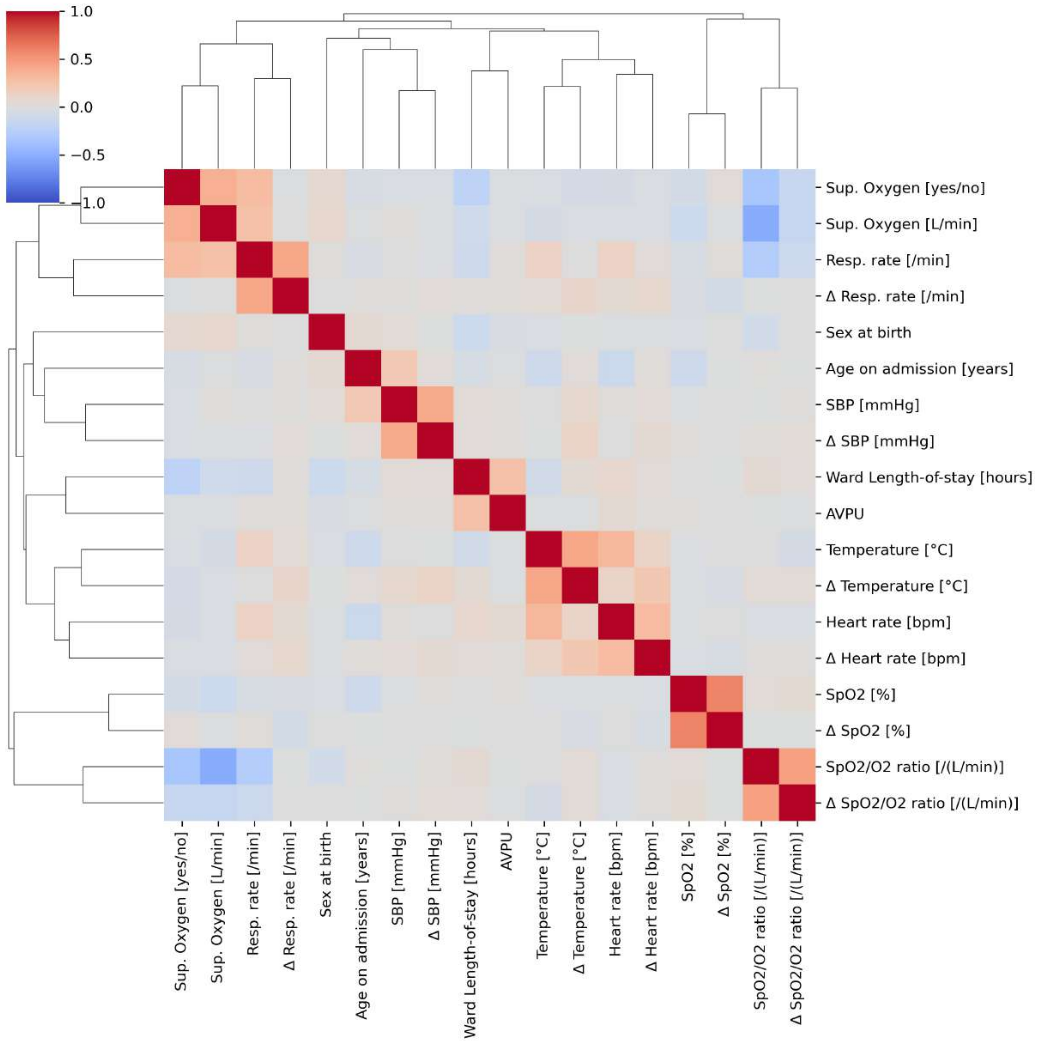

Figure 5: Clustered heatmap of the correlation matrix of all included model predictors.

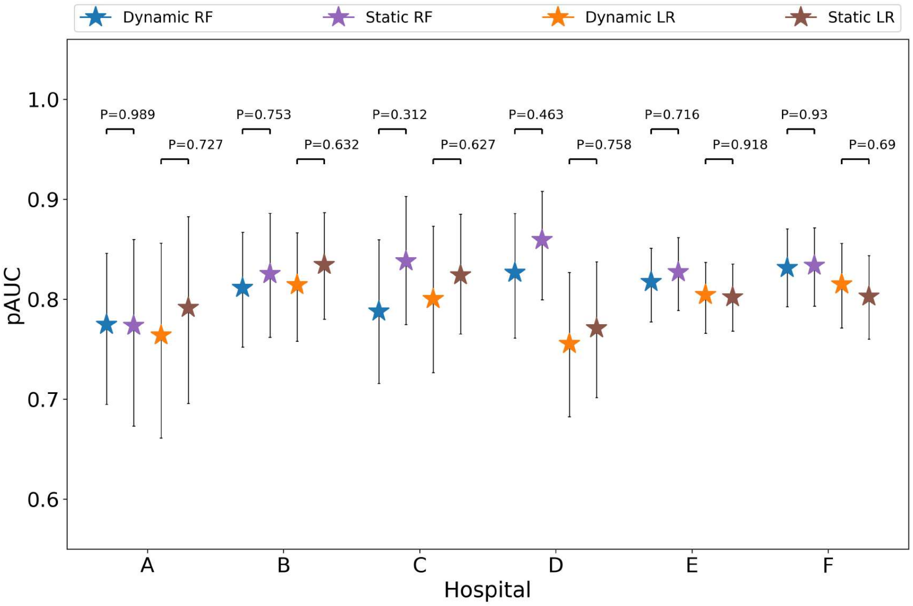

Figure 6: Discriminative performance of the static and dynamic random forest (RF) and logistic regression (LR) models in the temporal validation procedure in terms of partial area under the receiver operating characteristic curve (pAUC).

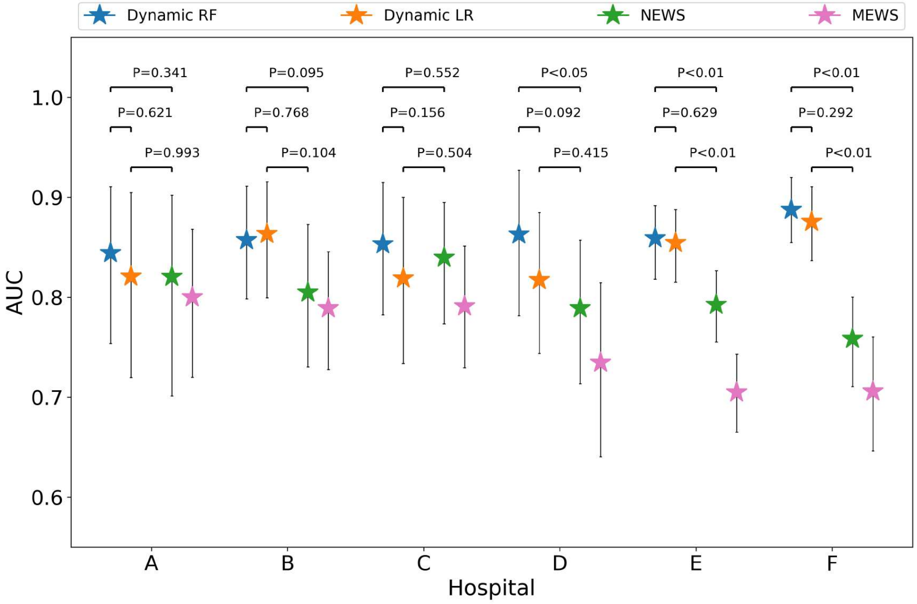

(a) AUC

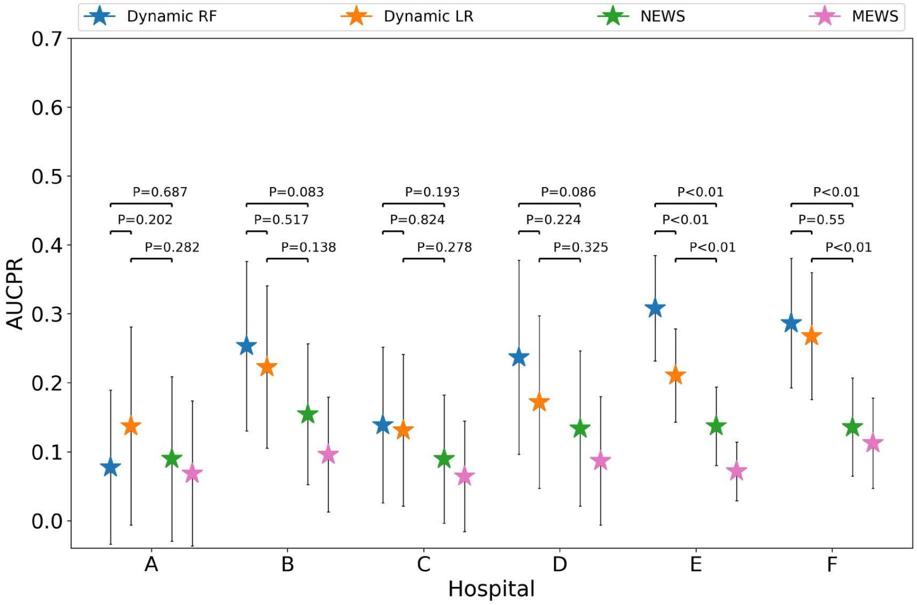

(b) AUCPR

Figure 7: Discriminative performance of the random forest (RF) and logistic regression (LR) models in the temporal validation procedure in terms of area under the receiver operating characteristic curve (AUC) and area under the precision-recall curve (AUCPR).

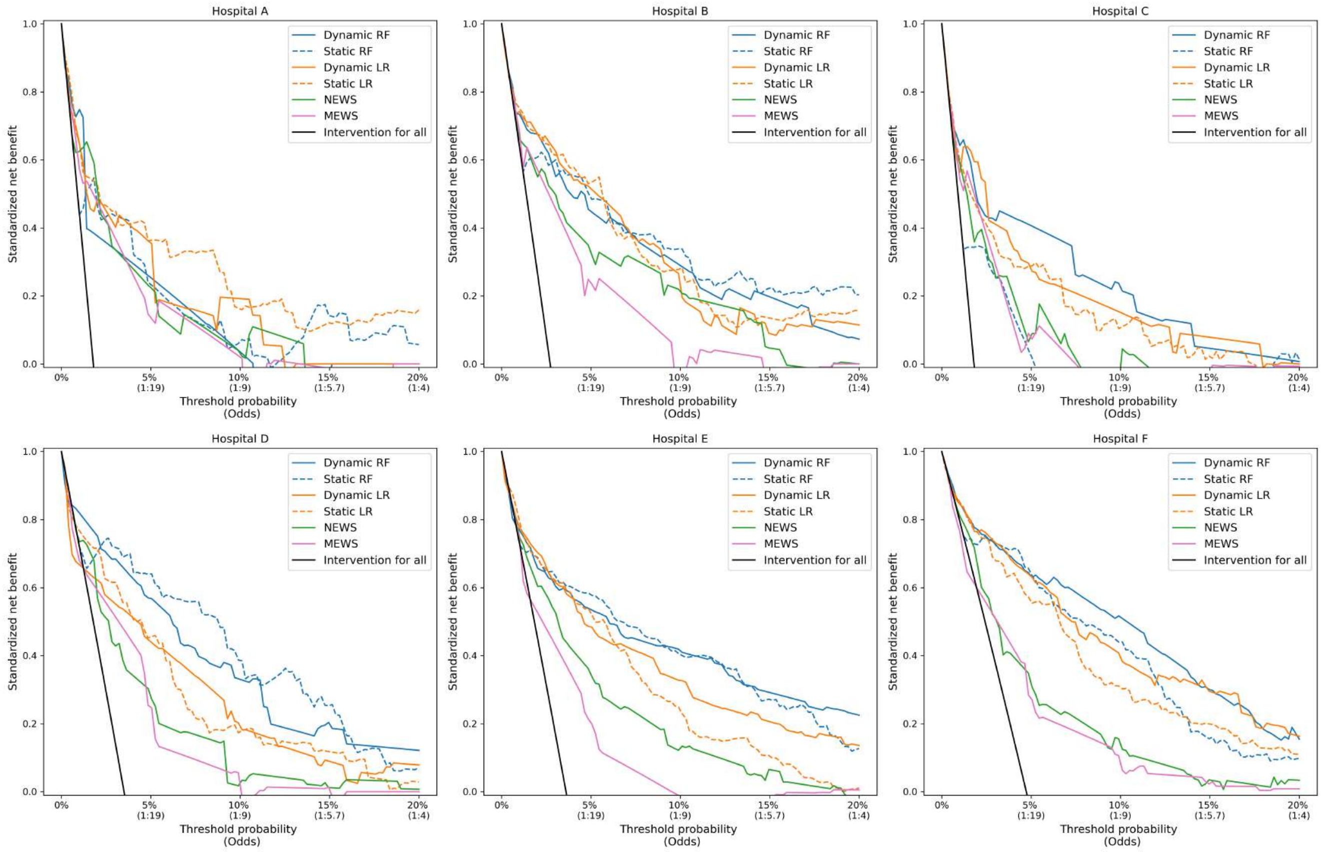

Figure 8: Hospital-specific decision curve analysis results in the temporal validation procedure. The standardized net benefits are plotted over a range of clinically relevant probability thresholds. The 'Intervention for all' lines indicate the net benefit (NB) if a (urgent or emergency) response would always be triggered.
RF=Random Forest, LR=Logistic Regression, MEWS=Modified Early Warning Score, NEWS=National Early Warning Score

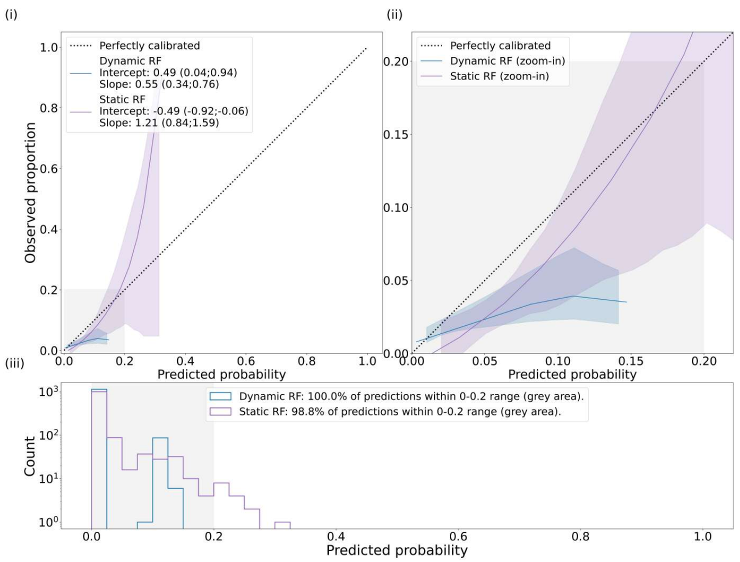

(a) RF hospital A.

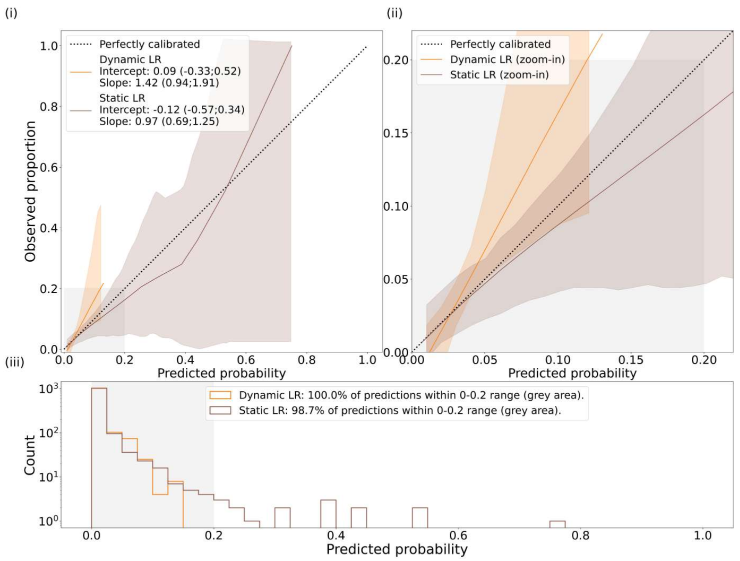

(b) LR hospital A.

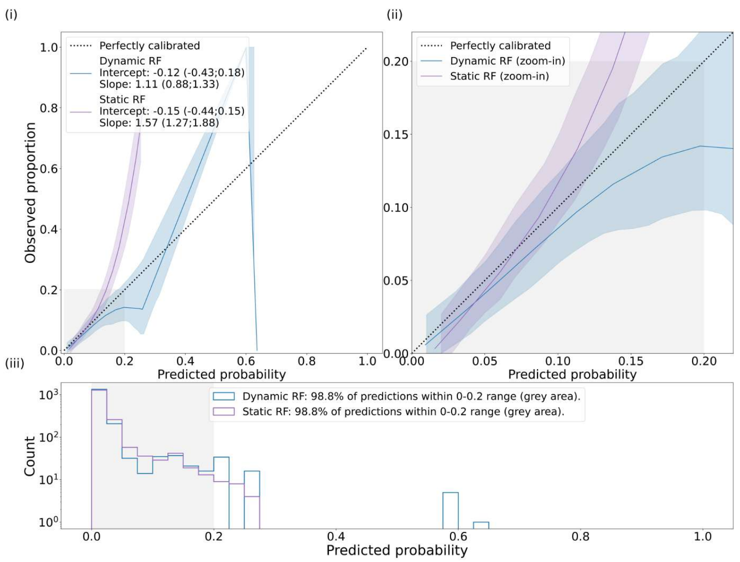

(c) RF hospital B.

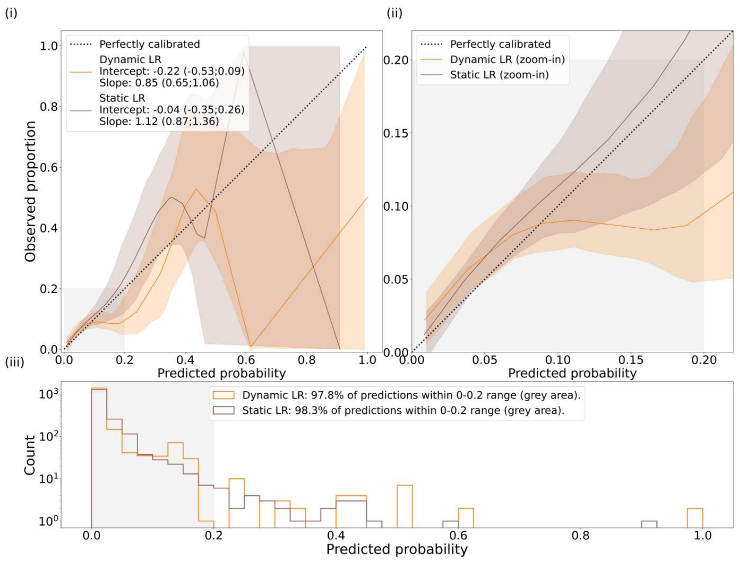

(d) LR hospital B.

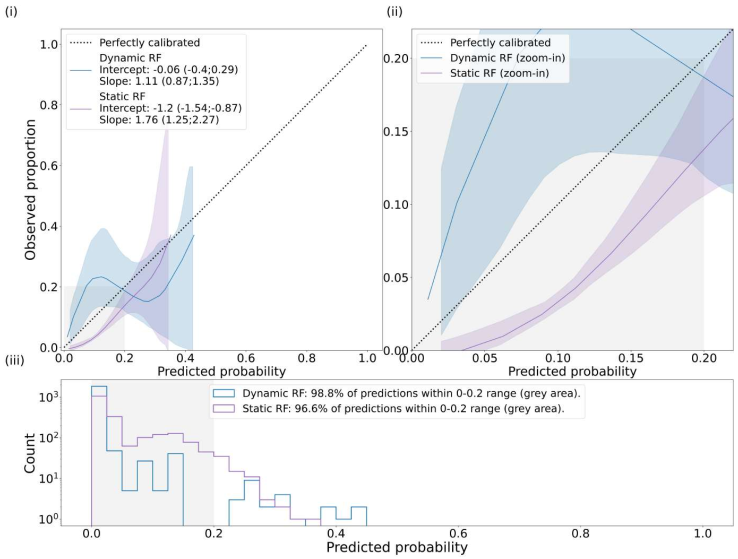

(e) RF hospital C.

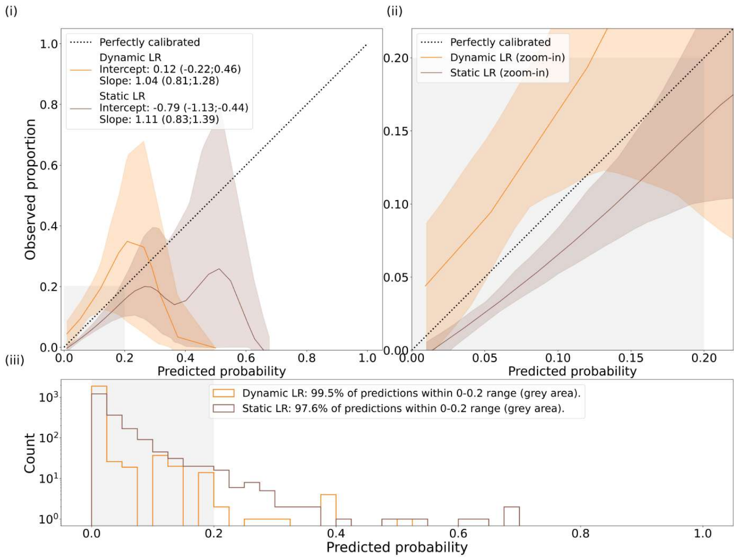

(f) LR hospital C.

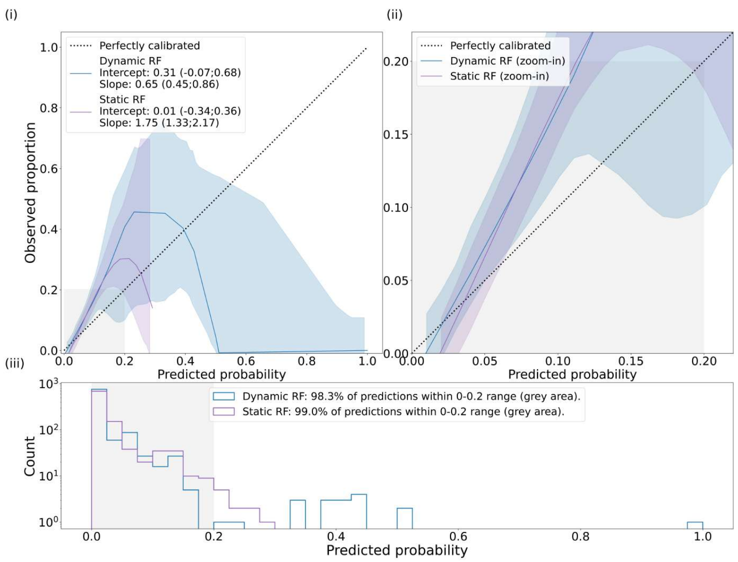

(g) RF hospital D.

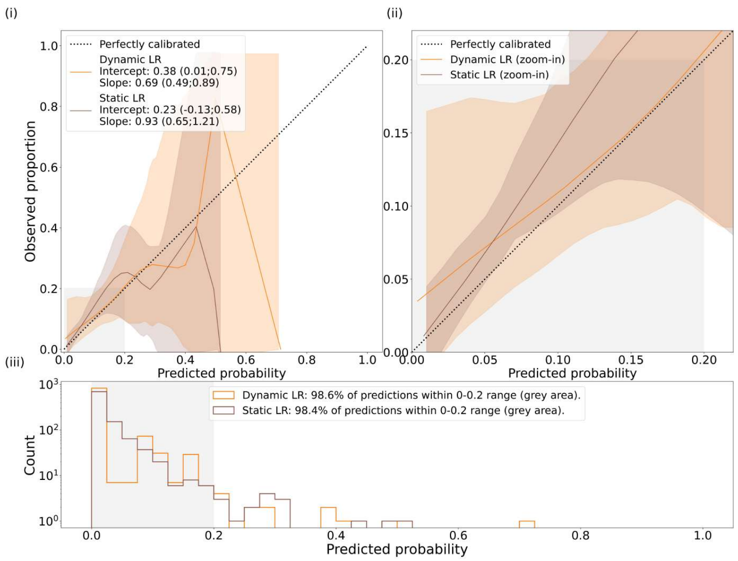

(h) LR hospital D.

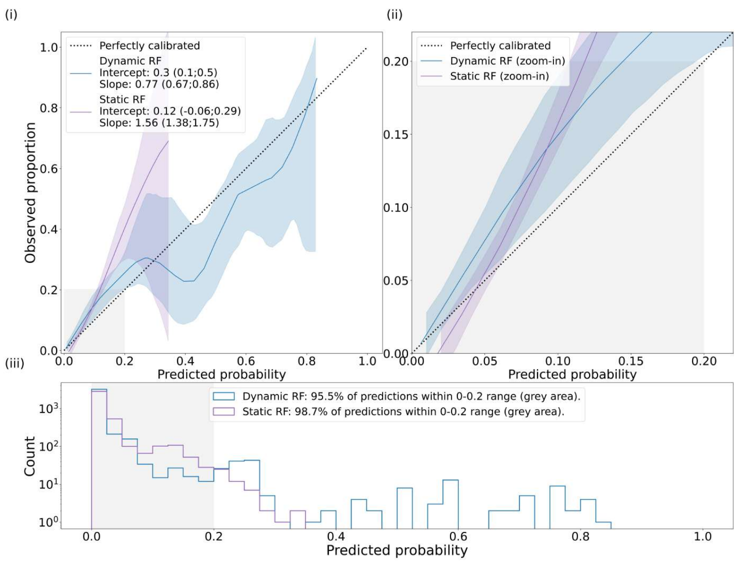

(i) RF hospital E.

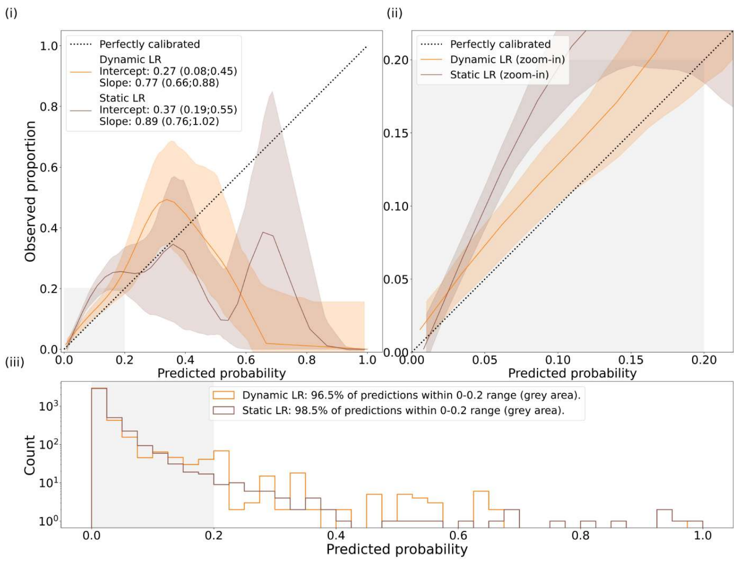

(j) LR hospital E.

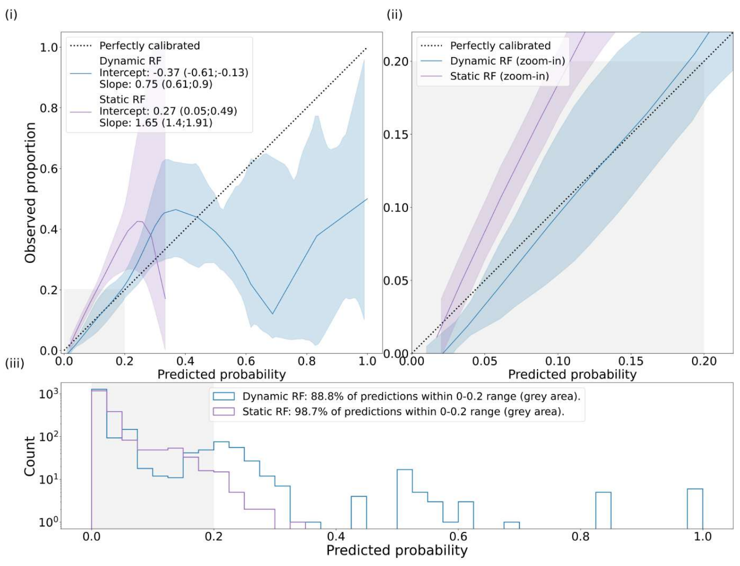

(k) RF hospital F.

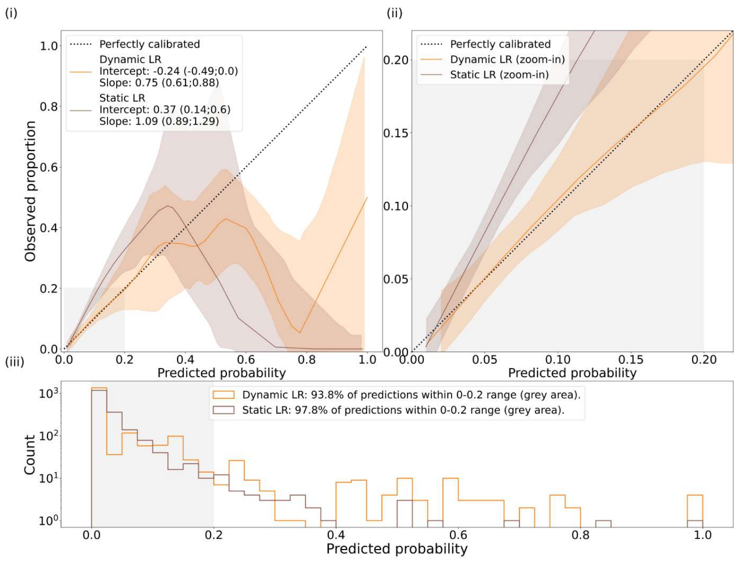

(l) LR hospital F.

Figure 9: Hospital-specific calibration curves of the static and dynamic random forest (RF) models and logistic regression (LR) models. (i) Smoothed flexible calibration curves. (ii) Zoom-in of the calibration curve in the 0-0.2 probability range (grey area). (iii) Histogram of the predictions (log scale). Shaded areas around each point in the calibration curves (before smoothing) represent the 95% bootstrap percentile CIs (with 1000 bootstrap replications stratified for positive and negative samples). The smooth curves including CIs were estimated by locally weighted scatterplot smoothing (see https://github.com/jimmsmit/COVID-19_EWS for the implementation).

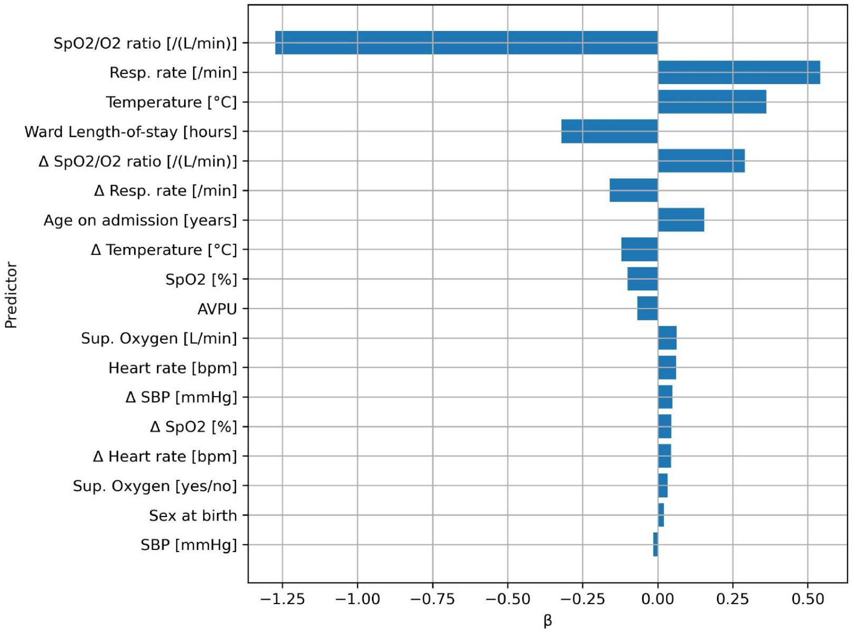

(a) Bar chart of model coefficients (βs). Model intercept = -4.01151618

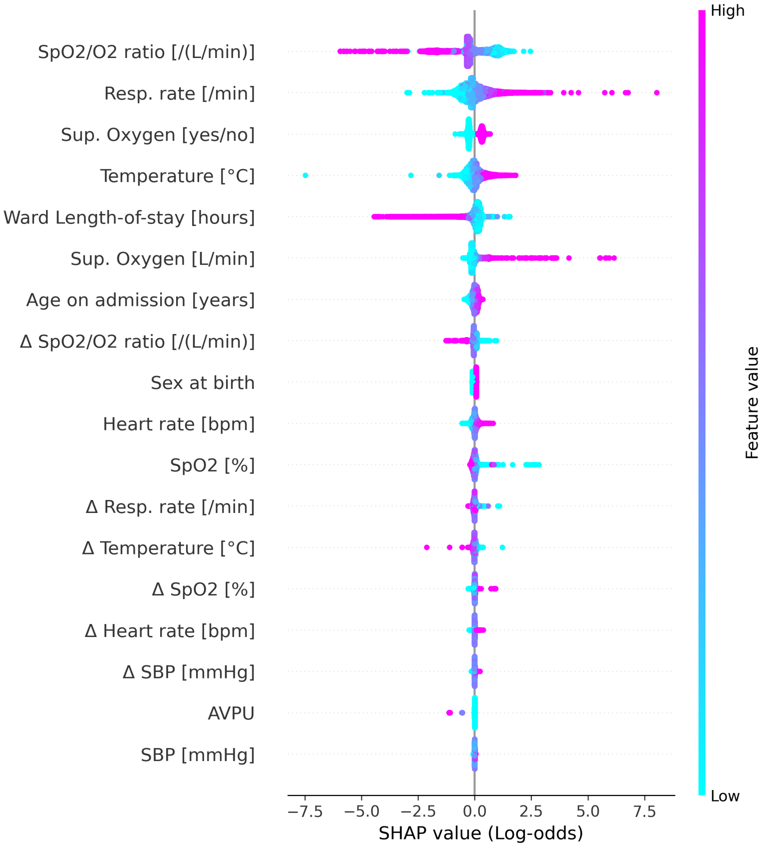

(b) Distribution of SHapley Additive exPlanations (SHAP) values of the included predictors (based on mean SHAP magnitude) for the logistic regression model. For each predictor, each dot represents the impact of that predictor for a single prediction. The colors of the dots correspond with the value for the specific predictor. Thus, pink dots with positive SHAP values indicate that high values of the predictor are associated with a high risk of clinical deterioration. Conversely, blue dots with positive SHAP values indicate that low values of the predictor are associated with a high risk of clinical deterioration.

Figure 10: Model interpretability for the logistic regression model fitted using the complete patient cohort.

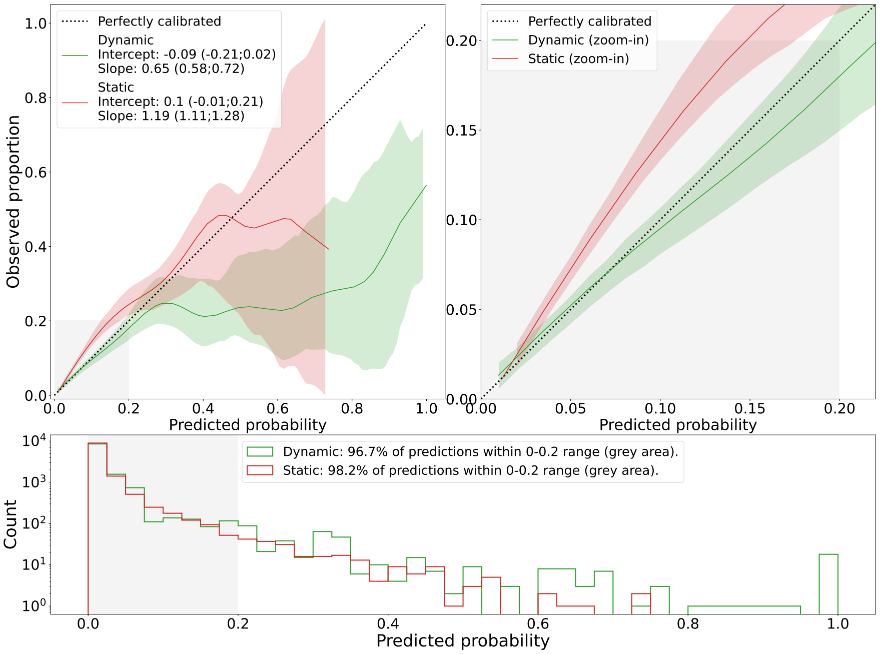

Figure 11: Calibration curves of the static and dynamic Gradient Boosting (XGB) models. (top left) Smoothed flexible calibration curves. (top right) Zoomin of the calibration curve in the 0-0.2 probability range (grey area). Shaded areas around the curves represent the 95% CIs. (bottom) Histogram of the predictions (log scale). Shaded areas around each point in the calibration curves (before smoothing) represent the 95% bootstrap percentile CIs (with 1000 bootstrap replications stratified for positive and negative samples). The smooth curves including CIs were estimated by locally weighted scatterplot smoothing (see https://github.com/jimmsmit/COVID-19_EWS for the implementation).

Appendix E: Supplementary tables

	Unit	Evidence	Included	Reason for Exclusion	Entry density
**Patient Demographics:**					
Age on admission	years	Knight et al.[6]	✓	-	-
Sex at Birth	-	Knight et al.[6]	✓	-	-
**Clinical Signs:**					
Respiratory Rate	breaths/min	Knight et al.[6]	✓	-	0.90
Peripheral oxygen saturation (SpO_2)	%	Knight et al.[6]	✓	-	0.92
Systolic blood pressure	mmHg	Knight et al.[6]	✓	-	0.92
Temperature	°C	Knight et al.[6]	✓	-	0.88
Heart Rate	bpm	Knight et al.[6]	✓	-	0.84
Glasgow Coma Score	-	Knight et al.[6]		Not available	-
AVPU	-	National Early Warning Score[7]	✓	-	0.50
Supplemental Oxygen	yes/no	National Early Warning Score[7]	✓	-	-
O_2	L/min	-	✓	-	-
SpO_2/O_2	$\frac{1}{L/min}$	-	✓	-	0.63
**Bedside investigations:**					
pH	-	Knight et al.[6]		Entry density <0.50	0.17
Glucose	mmol/L	Knight et al.[6]		Entry density <0.50	0.33
Infiltrates on chest radiograph	-	Knight et al.[6]		Not available	-
**Laboratory measures:**					
Haemoglobin	mmol/L	Knight et al.[6]		Entry density <0.50	0.46
Haematocrit	L/L	Knight et al.[6]		Entry density <0.50	0.38
White cell count	10^9/L	Knight et al.[6]		Entry density <0.50	0.41
Neutrophil count	10^9/L	Knight et al.[6]		Entry density <0.50	0.14
Lymphocyte count	10^9/L	Knight et al.[6]		Entry density <0.50	0.23
Eosinophil count	10^9/L	Xie et al.[8]		Entry density <0.50	0.22
Monocyte count	10^9/L	Linssen et al.[9]		Entry density <0.50	0.17
Neutrophil-to-lymphocyte ratio	-	Liu et al.[10]		Entry density <0.50	0.20
Platelet count	10^9/L	Knight et al.[6]		Entry density <0.50	0.39
Prothrombin	seconds	Knight et al.[6]		Not available	-
APTT	seconds	Knight et al.[6]		Entry density <0.50	0.05
Sodium	mmol/L	Knight et al.[6]		Entry density <0.50	0.45
Potassium	mmol/L	Knight et al.[6]		Entry density <0.50	0.45
Total Bilirubin	mg/dL	Knight et al.[6]		Entry density <0.50	0.28
ALAT	IU/L	Knight et al.[6]		Entry density <0.50	0.32
ASAT	IU/L	Knight et al.[6]		Entry density <0.50	0.32
Albumin	g/L	Knight et al.[6]		Entry density <0.50	0.17
Lactate dehydrogenase	IU/L	Knight et al.[6]		Entry density <0.50	0.31
Urea	mmol/L	Knight et al.[6]		Entry density <0.50	0.38
Creatinine	$\mu mol/L$	Knight et al.[6]		Entry density <0.50	0.46
C-reactive protein	mg/L	Knight et al.[6]		Entry density <0.50	0.34
RDW	%	Foy et al.[11]		Entry density <0.50	0.30
D-dimer	mg/L	Yu et al.[12]		Entry density <0.50	0.17
IL-6	pg/mL	Coomes et al.[13]		Entry density <0.50	0.002
Ferritin	$\mu g/L$	Dahan et al.[14]		Entry density <0.50	0.17
**Dynamics of clinical signs**					
Δ Respiratory Rate	breaths/min	-	✓	-	0.84
Δ Peripheral oxygen saturation	%	-	✓	-	0.87
Δ Systolic blood pressure	mmHg	-	✓	-	0.86
Δ Temperature	°C	-	✓	-	0.79
Δ Heart Rate	bpm	-	✓	-	0.81
Δ SpO2/O2	$\frac{1}{L/min}$	-	✓	-	0.54
**Other**					
Length of stay on ward	hours	-	✓	-	-

Table 1: Candidate predictors evaluated for potential inclusion in the prediction model, based on evidence in literature and availability. APPT = Activated Partial Thromboplastin Time. Δ = signed difference between first and second most recent measurement.

Model	Hyperparameter	Search Space
Logistic Regression	λ	[10^{-4}, …, 10^{4}] evenly spaced on log scale with 20 steps
Random Forest	number of trees	[500]
Random Forest	max features	[p, $\sqrt{p}$, $\log_2 p$] where p is the total number of predictors.
Random Forest	max three depth	[2,3]
Gradient boosting	number of trees	[500]
Gradient boosting	max depth	[3,5,6,10,15,20]
Gradient boosting	learning rate	[0.01, 0.1, 0.2, 0.3,3]
Gradient boosting	subsample	[0.5,0.6,0.7,0.8,0.9]
Gradient boosting	colsample by tree	[0.4,0.5,0.6,0.7,0.8,0.9]
Gradient boosting	colsample by level	[0.4,0.5,0.6,0.7,0.8,0.9]

Table 2: Search spaces used in the grid-search for model hyperparameters optimization.

Hospital	Period	N patients	Discharged alive (N, %)	Unplanned ICU admission (N,%)	Unexpected death (N,%)	Hospital transfer (N,%)	Still admitted (N,%)
A	Admission before August 1, 2020	91	71 (78.0)	18, (19.8)	0, (0.0)	2, (2.2)	0, (0.0)
	Admission after August 1, 2020	155	130 (83.9)	22, (14.2)	0, (0.0)	3, (1.9)	0, (0.0)
	Complete study period	246	201 (81.7)	40, (16.3)	0, (0.0)	5, (2.0)	0, (0.0)
B	Admission before August 1, 2020	205	167 (81.5)	38, (18.5)	0, (0.0)	0, (0.0)	0, (0.0)
	Admission after August 1, 2020	550	389 (70.7)	48, (8.7)	0, (0.0)	113, (20.5)	0, (0.0)
	Complete study period	755	556 (73.6)	86, (11.4)	0, (0.0)	113, (15.0)	0, (0.0)
C	Admission before August 1, 2020	233	190 (81.5)	41, (17.6)	0, (0.0)	2, (0.9)	0, (0.0)
	Admission after August 1, 2020	486	329 (67.7)	36, (7.4)	0, (0.0)	121, (24.9)	0, (0.0)
	Complete study period	719	519 (72.2)	77, (10.7)	0, (0.0)	123, (17.1)	0, (0.0)
D	Admission before August 1, 2020	61	42 (68.9)	19, (31.1)	0, (0.0)	0, (0.0)	0, (0.0)
	Admission after August 1, 2020	236	174 (73.7)	35, (14.8)	0, (0.0)	27, (11.4)	0, (0.0)
	Complete study period	297	216 (72.7)	54, (18.2)	0, (0.0)	27, (9.1)	0, (0.0)
E	Admission before August 1, 2020	132	109 (82.6)	21, (15.9)	0, (0.0)	2, (1.5)	0, (0.0)
	Admission after August 1, 2020	714	449 (62.9)	138, (19.3)	2, (0.3)	111, (15.5)	14, (2.0)
	Complete study period	846	558 (66.0)	159, (18.8)	2, (0.2)	113, (13.4)	14, (1.7)
F	Admission before August 1, 2020	123	88 (71.5)	34, (27.6)	0, (0.0)	1, (0.8)	0, (0.0)
	Admission after August 1, 2020	512	318 (62.1)	89, (17.4)	0, (0.0)	103, (20.1)	2, (0.4)
	Complete study period	635	406 (63.9)	123, (19.4)	0, (0.0)	104, (16.4)	2, (0.3)

Table 3: Occurence of different patient outcomes across the different hospitals over the whole study period and for patients admitted before and after August, 2020, separately.

Model	pAUC	AUC	AUCPR
MEWS	0.67 [0.65 to 0.69]	0.74 [0.72 to 0.77]	0.08 [0.05 to 0.11]
NEWS	0.72 [0.69 to 0.74]	0.72 [0.69 to 0.74]	0.12 [0.08 to 0.15]
Static RF	0.81 [0.79 to 0.83]	0.86 [0.84 to 0.88]	0.23 [0.19 to 0.27]
Static LR	0.80 [0.78 to 0.82]	0.86 [0.84 to 0.88]	0.18 [0.14 to 0.22]
Static XGB	0.81 [0.78 to 0.83]	0.87 [0.84 to 0.88]	0.22 [0.18 to 0.26]
Dynamic RF	0.82 [0.80 to 0.84]	0.86 [0.84 to 0.88]	0.26 [0.21 to 0.30]
Dynamic LR	0.81 [0.79 to 0.83]	0.85 [0.83 to 0.87]	0.21 [0.17 to 0.25]
Dynamic XGB	0.75 [0.72 to 0.77]	0.81 [0.78 to 0.83]	0.18 [0.14 to 0.22]

Table 4: Overall discriminative performance (based on the full test set) in terms of pAUC, AUC and AUCPR yielded by the different models. pAUC=partial area under the receiver operating characteristic curve, AUC=area under the receiver operating characteristic curve, AUCPR=area under the precision-recall curve.

	DA (N=667)	ICU (N=171)	Died (N=0)	Transfer (N=7)	SA (N=0)	Total (N=845)
Sex						
male , %	54.9	62.6	-	57.1	-	56.4
female , %	43.3	37.4	-	42.9	-	42.1
unknown , %	1.8	0.0	-	0.0	-	1.4
Age, years						
med (IQR)	59.0 (51.0-68.2)	62.0 (54.0-70.0)	-	58.0 (45.5-63.5)	-	59.0 (51.0-69.0)
mean (SD)	58.6 (13.7)	61.1 (12.0)	-	54.4 (14.2)	-	59.1 (13.5)
Ward LOS, days						
med (IQR)	4.2 (2.7-7.4)	2.2 (1.3-3.9)	-	3.3 (2.7-6.6)	-	3.8 (2.1-6.8)
mean (SD)	6.0 (8.9)	3.3 (4.1)	-	5.1 (3.8)	-	5.5 (8.2)
RR, breaths/min						
med (IQR)	18.0 (16.0-22.0)	20.0 (18.0-24.0)	-	22.0 (19.0-25.0)	-	20.0 (16.0-24.0)
mean (SD)	19.3 (5.1)	22.2 (5.9)	-	22.0 (4.5)	-	19.9 (5.4)
SpO2, %						
med (IQR)	96.0 (95.0-98.0)	95.0 (94.0-97.0)	-	97.0 (95.0-98.0)	-	96.0 (95.0-97.0)
mean (SD)	96.0 (4.2)	95.4 (2.4)	-	96.6 (1.4)	-	95.9 (3.9)
SBP, mmHg						
med (IQR)	125.0 (113.0-137.0)	125.0 (112.8-137.0)	-	126.0 (105.0-132.0)	-	125.0 (113.0-137.0)
mean (SD)	126.8 (18.0)	125.9 (17.0)	-	123.8 (20.7)	-	126.6 (17.8)
T, °C						
med (IQR)	37.5 (36.9-38.3)	37.9 (37.2-38.5)	-	37.2 (37.0-37.6)	-	37.6 (36.9-38.3)
mean (SD)	37.6 (0.9)	37.9 (0.9)	-	37.2 (0.5)	-	37.6 (0.9)
HR, bpm						
med (IQR)	83.0 (74.0-93.0)	87.0 (79.0-95.0)	-	81.0 (81.0-85.0)	-	84.0 (75.0-94.0)
mean (SD)	84.0 (13.8)	87.1 (13.9)	-	82.8 (4.4)	-	84.6 (13.8)
O2, yes/no, %	52.3	72.5	-	42.9	-	56.3
O2, L/min						
med (IQR)	3.0 (2.0-4.0)	5.0 (3.0-10.0)	-	3.0 (2.5-3.5)	-	3.0 (2.0-5.0)
mean (SD)	3.5 (2.9)	6.6 (4.6)	-	3.0 (0.8)	-	4.3 (3.7)
SpO2/O2, %/(L/min)						
med (IQR)	33.0 (23.5-49.0)	18.4 (9.6-31.7)	-	33.0 (28.4-41.0)	-	31.7 (18.8-48.5)
mean (SD)	42.7 (27.5)	26.3 (23.8)	-	35.2 (10.4)	-	38.3 (27.4)

Table 5: Pathway and population characteristics of the patients admitted before August 1, 2020, measured within the first 24 hours of hospital admission. med = median, IQR = inter-quartile range, SD = standard deviation, LOS = length-of-stay, RR = respiratory rate, SpO2 = Peripheral oxygen saturation, SBP = Systolic blood pressure, T = Temperature, HR = Heart rate, O2 = Supplemental oxygen, SpO2/O2 = SpO2-to-O2 ratio..

	DA (N=1805)	ICU (N=368)	Died (N=2)	Transfer (N=478)	SA (N=16)	Total (N=2669)
Sex						
male , %	55.5	65.8	100.0	56.9	56.2	57.2
female , %	43.0	33.7	0.0	39.7	43.8	41.1
unknown , %	1.5	0.5	0.0	3.3	0.0	1.7
Age, years						
med (IQR)	61.0 (51.0-71.0)	63.0 (56.0-70.0)	76.0 (74.5-77.5)	60.0 (53.5-69.0)	66.5 (55.0-75.5)	61.0 (52.0-70.0)
mean (SD)	60.0 (14.3)	61.8 (11.5)	76.0 (3.0)	59.9 (11.8)	64.5 (11.6)	60.3 (13.5)
Ward LOS, days						
med (IQR)	3.5 (1.8-5.9)	2.3 (1.0-3.9)	7.6 (7.2-7.9)	1.1 (0.8-2.0)	4.7 (1.2-14.5)	2.7 (1.3-5.0)
mean (SD)	5.0 (5.8)	3.4 (4.6)	7.6 (0.7)	1.8 (2.2)	8.6 (8.9)	4.2 (5.3)
RR, breaths/min						
med (IQR)	18.0 (16.0-22.0)	24.0 (20.0-26.0)	20.0 (20.0-20.0)	20.0 (16.8-24.0)	18.0 (16.0-23.5)	20.0 (16.0-24.0)
mean (SD)	19.3 (5.0)	23.3 (6.0)	20.0 (0.0)	20.8 (5.1)	20.7 (6.2)	20.2 (5.3)
SpO2, %						
med (IQR)	96.0 (95.0-98.0)	95.0 (94.0-97.0)	95.5 (95.2-95.8)	95.0 (94.0-97.0)	95.0 (93.5-97.0)	96.0 (94.0-97.0)
mean (SD)	96.0 (3.1)	95.0 (5.5)	95.5 (0.5)	95.5 (2.2)	94.5 (3.4)	95.8 (3.4)
SBP, mmHg						
med (IQR)	125.0 (113.0-137.0)	125.0 (114.0-137.0)	137.5 (136.8-138.2)	122.5 (113.0-133.8)	119.0 (107.2-126.8)	124.0 (113.0-136.0)
mean (SD)	126.2 (19.1)	128.1 (20.3)	137.5 (1.5)	124.1 (16.5)	120.0 (17.3)	126.1 (18.8)
T, °C						
med (IQR)	37.0 (36.5-37.5)	37.0 (36.6-37.6)	37.0 (37.0-37.1)	37.0 (36.6-37.7)	36.8 (36.2-37.0)	37.0 (36.5-37.6)
mean (SD)	37.1 (0.9)	37.2 (0.9)	37.0 (0.1)	37.2 (0.9)	36.8 (0.7)	37.1 (0.9)
HR, bpm						
med (IQR)	80.0 (71.0-90.0)	81.0 (72.0-90.0)	91.0 (84.0-98.0)	81.0 (72.0-90.0)	80.0 (73.8-84.8)	80.0 (71.0-90.0)
mean (SD)	81.2 (15.6)	81.8 (15.5)	91.0 (14.0)	81.6 (13.7)	81.2 (13.6)	81.4 (15.2)
O2, yes/no, %	59.3	78.3	0.0	83.1	68.8	66.2
O2, L/min						
med (IQR)	3.0 (2.0-4.0)	6.0 (4.0-12.0)	-	4.0 (2.0-5.0)	3.0 (2.0-7.5)	3.0 (2.0-5.0)
mean (SD)	3.7 (3.1)	8.0 (5.7)	-	4.4 (3.0)	5.5 (5.1)	4.5 (3.9)
SpO2/O2, 1/(L/min)						
med (IQR)	32.7 (23.5-48.5)	15.5 (8.0-23.8)	-	24.2 (18.6-47.0)	30.7 (14.1-48.5)	31.3 (18.4-48.0)
mean (SD)	41.1 (26.1)	21.5 (20.0)	-	32.4 (21.2)	35.0 (24.1)	35.9 (25.2)

Table 6: Pathway and population characteristics of the patients admitted after August 1, 2020, measured within the first 24 hours of hospital admission. med = median, IQR = inter-quartile range, SD = standard deviation, LOS = length-of-stay, RR = respiratory rate, SpO2 = Peripheral oxygen saturation, SBP = Systolic blood pressure, T = Temperature, HR = Heart rate, O2 = Supplemental oxygen, SpO2/O2 = SpO2-to-O2 ratio.

	DA (N=201)	ICU (N=40)	Died (N=0)	Transfer (N=5)	SA (N=0)	Total (N=246)
Sex						
male , %	51.2	50.0	-	40.0	-	50.8
female , %	48.8	50.0	-	60.0	-	49.2
unknown , %	0.0	0.0	-	0.0	-	0.0
Age, years						
med (IQR)	60.0 (48.0-69.0)	66.5 (50.8-73.0)	-	59.5 (56.2-63.8)	-	60.0 (50.0-70.0)
mean (SD)	56.3 (16.4)	61.6 (14.5)	-	60.5 (5.7)	-	57.3 (16.1)
Ward LOS, days						
med (IQR)	6.8 (3.8-10.9)	2.2 (1.1-4.1)	-	5.2 (5.1-7.2)	-	5.7 (3.0-10.3)
mean (SD)	9.9 (14.9)	4.3 (7.0)	-	5.9 (1.5)	-	8.9 (14.0)
RR, breaths/min						
med (IQR)	16.0 (14.0-20.0)	20.0 (17.0-25.0)	-	15.0 (13.5-16.5)	-	16.5 (14.0-20.0)
mean (SD)	18.2 (5.5)	21.8 (7.2)	-	15.0 (3.0)	-	18.8 (6.0)
SpO2, %						
med (IQR)	96.0 (94.5-97.0)	95.0 (93.0-96.0)	-	96.0 (96.0-96.0)	-	96.0 (94.0-97.0)
mean (SD)	95.9 (2.1)	94.5 (2.8)	-	96.0 (0.0)	-	95.6 (2.3)
SBP, mmHg						
med (IQR)	124.0 (115.0-135.0)	132.0 (116.0-138.5)	-	124.5 (119.8-129.2)	-	125.0 (115.0-136.0)
mean (SD)	125.7 (17.9)	131.6 (23.4)	-	124.5 (9.5)	-	126.8 (19.1)
T, °C						
med (IQR)	38.0 (37.0-38.0)	38.0 (37.2-38.8)	-	-	-	38.0 (37.0-38.0)
mean (SD)	37.6 (1.0)	38.2 (1.1)	-	-	-	37.7 (1.0)
HR, bpm						
med (IQR)	85.5 (73.8-97.2)	84.5 (75.0-88.8)	-	88.0 (79.0-97.0)	-	85.0 (73.2-97.0)
mean (SD)	86.3 (16.6)	83.0 (15.3)	-	88.0 (18.0)	-	85.7 (16.5)
O2, yes/no, %	23.9	35.0	-	40.0	-	26.0
O2, L/min						
med (IQR)	3.0 (2.0-4.0)	5.0 (3.0-15.0)	-	10.0 (7.5-12.5)	-	4.0 (2.0-5.0)
mean (SD)	4.3 (3.7)	7.5 (5.4)	-	10.0 (5.0)	-	5.1 (4.4)
SpO2/O2, 1/(L/min)						
med (IQR)	31.3 (23.3-48.0)	18.2 (6.6-31.0)	-	12.8 (9.6-16.0)	-	24.0 (18.2-47.5)
mean (SD)	36.2 (24.9)	25.6 (24.7)	-	12.8 (6.4)	-	33.2 (25.2)

Table 7: Pathway and population characteristics of the patients in hospital A measured within the first 24 hours of hospital admission. med = median, IQR = inter-quartile range, SD = standard deviation, LOS = length-of-stay, RR = respiratory rate, SpO2 = Peripheral oxygen saturation, SBP = Systolic blood pressure, T = Temperature, HR = Heart rate, O2 = Supplemental oxygen, SpO2/O2 = SpO2-to-O2 ratio.

	DA (N=556)	ICU (N=68)	Died (N=0)	Transfer (N=113)	SA (N=0)	Total (N=755)
Sex						
male , %	52.0	73.3	-	54.9	-	54.8
female , %	43.0	26.7	-	34.5	-	39.9
unknown , %	5.0	0.0	-	10.6	-	5.3
Age, years						
med (IQR)	59.0 (49.0-68.0)	58.0 (50.0-66.5)	-	59.0 (53.0-68.0)	-	59.0 (50.0-68.0)
mean (SD)	58.0 (13.6)	57.2 (13.2)	-	58.6 (11.7)	-	58.0 (13.3)
Ward LOS, days						
med (IQR)	2.9 (1.6-4.9)	1.6 (1.0-2.8)	-	1.1 (0.9-1.7)	-	2.2 (1.1-4.3)
mean (SD)	4.0 (4.7)	2.3 (2.1)	-	1.7 (3.0)	-	3.5 (4.3)
RR, breaths/min						
med (IQR)	16.5 (16.0-20.0)	20.0 (18.0-24.0)	-	20.0 (16.0-24.0)	-	18.0 (16.0-20.0)
mean (SD)	18.0 (4.1)	22.5 (6.6)	-	20.1 (4.9)	-	18.8 (4.9)
SpO2, %						
med (IQR)	97.0 (95.0-98.0)	97.0 (95.0-98.0)	-	96.0 (95.0-97.0)	-	97.0 (95.0-98.0)
mean (SD)	96.6 (4.5)	96.6 (2.1)	-	95.9 (2.4)	-	96.5 (4.1)
SBP, mmHg						
med (IQR)	126.0 (113.0-138.0)	124.5 (114.0-136.0)	-	125.0 (113.0-133.0)	-	125.0 (113.0-137.0)
mean (SD)	128.2 (19.9)	126.1 (17.7)	-	124.9 (17.8)	-	127.4 (19.4)
T, °C						
med (IQR)	37.3 (36.8-38.0)	37.8 (37.2-38.6)	-	37.5 (37.1-38.0)	-	37.4 (36.8-38.1)
mean (SD)	37.4 (0.9)	37.9 (1.0)	-	37.6 (0.9)	-	37.5 (0.9)
HR, bpm						
med (IQR)	83.0 (74.0-93.0)	89.5 (79.0-100.8)	-	85.0 (76.0-96.0)	-	84.0 (75.0-94.0)
mean (SD)	83.7 (14.7)	90.0 (16.8)	-	86.2 (14.6)	-	84.8 (15.1)
O2, yes/no, %	41.9	67.4	-	61.9	-	47.8
O2, L/min						
med (IQR)	2.0 (2.0-4.0)	6.0 (4.0-10.0)	-	4.0 (2.1-5.0)	-	3.0 (2.0-5.0)
mean (SD)	3.4 (2.7)	7.9 (7.9)	-	4.6 (3.0)	-	4.4 (4.4)
SpO2/O2, 1/(L/min)						
med (IQR)	40.5 (24.0-49.0)	15.9 (9.6-23.7)	-	24.0 (19.0-47.0)	-	31.7 (19.2-48.0)
mean (SD)	41.4 (25.9)	20.5 (15.2)	-	29.6 (18.2)	-	35.6 (24.4)

Table 8: Pathway and population characteristics of the patients in hospital B measured within the first 24 hours of hospital admission. med = median, IQR = inter-quartile range, SD = standard deviation, LOS = length-of-stay, RR = respiratory rate, SpO2 = Peripheral oxygen saturation, SBP = Systolic blood pressure, T = Temperature, HR = Heart rate, O2 = Supplemental oxygen, SpO2/O2 = SpO2-to-O2 ratio.

	DA (N=519)	ICU (N=77)	Died (N=0)	Transfer (N=123)	SA (N=0)	Total (N=719)
Sex						
male , %	60.3	70.1	-	65.0	-	62.2
female , %	39.1	29.9	-	34.1	-	37.3
unknown , %	0.6	0.0	-	0.8	-	0.6
Age, years						
med (IQR)	59.0 (49.0-68.0)	63.0 (57.0-69.0)	-	60.0 (53.0-65.8)	-	60.0 (51.0-68.0)
mean (SD)	58.4 (14.7)	62.3 (9.3)	-	59.0 (11.2)	-	58.9 (13.7)
Ward LOS, days						
med (IQR)	3.0 (1.8-5.8)	3.2 (1.8-4.4)	-	1.0 (0.7-2.5)	-	2.8 (1.5-4.9)
mean (SD)	4.8 (5.1)	3.9 (3.6)	-	1.8 (1.6)	-	4.2 (4.7)
RR, breaths/min						
med (IQR)	18.0 (16.0-22.0)	23.0 (18.0-28.0)	-	20.0 (16.0-24.0)	-	20.0 (16.0-24.0)
mean (SD)	19.5 (5.1)	23.1 (6.0)	-	20.2 (4.8)	-	20.0 (5.3)
SpO2, %						
med (IQR)	96.0 (95.0-97.0)	95.0 (94.0-96.0)	-	95.0 (94.0-96.0)	-	96.0 (94.0-97.0)
mean (SD)	95.7 (4.6)	93.5 (10.7)	-	94.9 (2.2)	-	95.3 (5.4)
SBP, mmHg						
med (IQR)	125.0 (114.0-138.0)	120.0 (114.0-133.0)	-	124.0 (114.5-135.0)	-	124.0 (114.0-136.0)
mean (SD)	127.0 (17.9)	123.7 (16.5)	-	125.3 (17.7)	-	126.3 (17.7)
T, °C						
med (IQR)	37.3 (36.7-38.0)	37.4 (36.8-38.3)	-	37.0 (36.5-37.6)	-	37.2 (36.6-38.0)
mean (SD)	37.3 (1.0)	37.5 (0.9)	-	37.1 (0.9)	-	37.3 (1.0)
HR, bpm						
med (IQR)	82.0 (73.0-92.0)	84.0 (75.0-90.0)	-	82.0 (75.5-90.0)	-	82.0 (73.0-91.0)
mean (SD)	83.1 (14.3)	83.3 (12.4)	-	82.3 (11.8)	-	83.0 (13.7)
O2, yes/no, %	63.2	83.1	-	88.6	-	69.7
O2, L/min						
med (IQR)	3.0 (2.0-5.0)	9.0 (4.0-15.0)	-	4.0 (2.0-6.0)	-	3.0 (2.0-6.0)
mean (SD)	4.3 (3.9)	9.1 (5.4)	-	5.1 (3.9)	-	5.1 (4.4)
SpO2/O2, 1/(L/min)						
med (IQR)	32.0 (18.8-48.5)	10.7 (6.3-23.5)	-	23.8 (15.7-47.0)	-	31.0 (15.8-48.0)
mean (SD)	39.7 (27.7)	20.5 (23.1)	-	31.7 (24.4)	-	35.5 (27.3)

Table 9: Pathway and population characteristics of the patients in hospital C measured within the first 24 hours of hospital admission. med = median, IQR = inter-quartile range, SD = standard deviation, LOS = length-of-stay, RR = respiratory rate, SpO2 = Peripheral oxygen saturation, SBP = Systolic blood pressure, T = Temperature, HR = Heart rate, O2 = Supplemental oxygen, SpO2/O2 = SpO2-to-O2 ratio.

	DA (N=216)	ICU (N=54)	Died (N=0)	Transfer (N=27)	SA (N=0)	Total (N=297)
Sex						
male , %	60.2	57.4	-	40.7	-	57.9
female , %	39.8	42.6	-	59.3	-	42.1
unknown , %	0.0	0.0	-	0.0	-	0.0
Age, years						
med (IQR)	62.0 (54.0-71.0)	65.0 (58.5-72.0)	-	60.0 (53.5-71.5)	-	63.0 (54.0-71.0)
mean (SD)	61.3 (12.9)	64.5 (10.3)	-	61.3 (12.8)	-	61.9 (12.5)
Ward LOS, days						
med (IQR)	3.9 (2.6-6.0)	2.3 (1.4-4.0)	-	1.3 (1.0-2.0)	-	3.4 (1.9-5.2)
mean (SD)	4.8 (4.3)	3.7 (4.9)	-	1.8 (1.2)	-	4.4 (4.3)
RR, breaths/min						
med (IQR)	20.0 (16.0-22.0)	24.0 (20.0-28.0)	-	23.0 (20.0-25.8)	-	20.0 (16.0-24.0)
mean (SD)	19.6 (4.9)	24.4 (7.5)	-	22.5 (5.5)	-	20.8 (5.9)
SpO2, %						
med (IQR)	97.0 (96.0-99.0)	96.0 (95.0-97.0)	-	97.0 (96.0-98.0)	-	97.0 (96.0-98.0)
mean (SD)	97.2 (2.2)	96.1 (2.2)	-	96.7 (1.7)	-	96.9 (2.2)
SBP, mmHg						
med (IQR)	130.0 (118.0-141.0)	124.5 (115.2-138.2)	-	121.5 (117.5-133.8)	-	128.0 (117.0-140.0)
mean (SD)	130.7 (19.4)	129.3 (22.3)	-	125.3 (12.8)	-	130.0 (19.5)
T, °C						
med (IQR)	37.0 (36.6-37.7)	37.3 (36.6-37.9)	-	37.1 (36.7-37.7)	-	37.1 (36.7-37.7)
mean (SD)	37.2 (0.9)	37.4 (0.9)	-	37.4 (0.9)	-	37.2 (0.9)
HR, bpm						
med (IQR)	88.0 (87.0-89.0)	103.0 (103.0-103.0)	-	97.0 (97.0-97.0)	-	93.5 (89.0-98.5)
mean (SD)	88.0 (2.0)	103.0 (0.0)	-	97.0 (0.0)	-	94.0 (6.5)
O2, yes/no, %	76.4	92.6	-	85.2	-	80.1
O2, L/min						
med (IQR)	3.0 (2.0-4.0)	6.0 (4.0-10.0)	-	5.0 (3.0-6.5)	-	3.0 (2.0-5.4)
mean (SD)	3.5 (2.3)	7.4 (4.5)	-	5.4 (3.3)	-	4.5 (3.4)
SpO2/O2, 1/(L/min)						
med (IQR)	32.7 (24.2-48.5)	15.9 (9.4-23.8)	-	19.4 (15.1-32.3)	-	31.7 (18.3-48.0)
mean (SD)	39.6 (23.6)	22.1 (21.5)	-	24.4 (12.5)	-	34.4 (23.6)

Table 10: Pathway and population characteristics of the patients in hospital D measured within the first 24 hours of hospital admission. med = median, IQR = inter-quartile range, SD = standard deviation, LOS = length-of-stay, RR = respiratory rate, SpO2 = Peripheral oxygen saturation, SBP = Systolic blood pressure, T = Temperature, HR = Heart rate, O2 = Supplemental oxygen, SpO2/O2 = SpO2-to-O2 ratio.

	DA (N=572)	ICU (N=159)	Died (N=2)	Transfer (N=113)	SA (N=14)	Total (N=860)
Sex						
male , %	57.7	62.9	100.0	59.3	64.3	59.1
female , %	42.3	37.1	0.0	40.7	35.7	40.9
unknown , %	0.0	0.0	0.0	0.0	0.0	0.0
Age, years						
med (IQR)	64.0 (54.0-73.0)	63.0 (55.2-69.0)	76.0 (74.5-77.5)	61.0 (55.0-71.5)	67.0 (55.0-76.5)	64.0 (55.0-72.0)
mean (SD)	62.6 (13.6)	62.1 (10.4)	76.0 (3.0)	61.4 (11.6)	64.9 (12.3)	62.4 (12.8)
Ward LOS, days						
med (IQR)	4.5 (2.2-7.8)	2.5 (1.1-4.2)	7.6 (7.2-7.9)	1.4 (0.9-2.7)	5.8 (1.2-15.4)	3.4 (1.7-6.6)
mean (SD)	6.1 (6.7)	3.9 (5.3)	7.6 (0.7)	2.3 (2.7)	9.3 (9.2)	5.2 (6.3)
RR, breaths/min						
med (IQR)	20.0 (16.0-22.0)	22.0 (18.0-24.0)	20.0 (20.0-20.0)	20.0 (18.0-24.0)	18.0 (16.0-23.0)	20.0 (17.0-24.0)
mean (SD)	20.2 (5.9)	22.6 (5.4)	20.0 (0.0)	20.9 (4.7)	19.9 (6.0)	20.7 (5.7)
SpO2, %						
med (IQR)	96.0 (94.0-97.0)	95.0 (94.0-96.0)	95.5 (95.2-95.8)	95.0 (94.0-97.0)	95.0 (92.5-96.8)	95.0 (94.0-97.0)
mean (SD)	95.6 (2.1)	94.9 (2.2)	95.5 (0.5)	95.3 (2.1)	94.4 (3.5)	95.4 (2.1)
SBP, mmHg						
med (IQR)	123.0 (112.0-135.0)	128.0 (116.0-143.5)	137.5 (136.8-138.2)	124.0 (114.0-132.0)	119.0 (109.0-124.0)	124.0 (113.0-136.0)
mean (SD)	124.6 (18.6)	130.6 (21.2)	137.5 (1.5)	124.5 (15.2)	119.1 (14.7)	125.6 (18.8)
T, °C						
med (IQR)	36.8 (36.3-37.4)	36.9 (36.5-37.5)	37.0 (37.0-37.1)	36.8 (36.3-37.4)	36.8 (36.3-37.0)	36.8 (36.3-37.4)
mean (SD)	36.9 (0.9)	37.0 (0.8)	37.0 (0.1)	36.9 (0.9)	36.8 (0.8)	36.9 (0.8)
HR, bpm						
med (IQR)	79.0 (70.0-90.0)	79.0 (70.5-88.0)	91.0 (84.0-98.0)	78.0 (70.0-87.0)	80.0 (76.8-83.5)	79.0 (70.0-89.8)
mean (SD)	80.9 (16.1)	80.0 (13.5)	91.0 (14.0)	79.1 (14.5)	81.1 (13.4)	80.5 (15.4)
O2, yes/no, %	64.9	81.8	0.0	92.9	64.3	71.5
O2, L/min						
med (IQR)	3.0 (2.0-4.0)	5.0 (3.0-12.0)	-	3.0 (2.0-5.0)	2.0 (2.0-3.0)	3.0 (2.0-5.0)
mean (SD)	3.5 (2.6)	7.2 (4.9)	-	3.8 (2.4)	3.9 (4.1)	4.3 (3.6)
SpO2/O2, 1/(L/min)						
med (IQR)	32.3 (23.5-48.0)	18.2 (8.1-31.4)	-	31.0 (19.0-47.5)	47.5 (30.0-49.0)	31.7 (18.8-48.0)
mean (SD)	41.1 (25.6)	23.4 (19.8)	-	35.5 (22.6)	41.1 (22.5)	36.5 (24.9)

Table 11: Pathway and population characteristics of the patients in hospital E measured within the first 24 hours of hospital admission. med = median, IQR = inter-quartile range, SD = standard deviation, LOS = length-of-stay, RR = respiratory rate, SpO2 = Peripheral oxygen saturation, SBP = Systolic blood pressure, T = Temperature, HR = Heart rate, O2 = Supplemental oxygen, SpO2/O2 = SpO2-to-O2 ratio.

	DA (N=408)	ICU (N=123)	Died (N=0)	Transfer (N=104)	SA (N=2)	Total (N=637)
Sex						
male , %	49.8	65.9	-	51.9	0.0	53.1
female , %	48.3	32.5	-	45.2	100.0	44.9
unknown , %	2.0	1.6	-	2.9	0.0	2.0
Age, years						
med (IQR)	61.0 (52.0-70.0)	64.0 (55.0-71.0)	-	61.0 (52.0-69.0)	62.0 (60.0-64.0)	61.0 (52.0-70.0)
mean (SD)	59.9 (13.4)	62.0 (12.1)	-	59.7 (12.9)	62.0 (4.0)	60.3 (13.1)
Ward LOS, days						
med (IQR)	3.4 (1.9-5.5)	2.1 (1.0-3.7)	-	0.9 (0.7-1.4)	3.8 (2.5-5.1)	2.7 (1.3-4.6)
mean (SD)	4.4 (3.8)	2.8 (3.2)	-	1.4 (1.5)	3.8 (2.5)	3.6 (3.6)
RR, breaths/min						
med (IQR)	20.0 (16.0-24.0)	24.0 (20.0-26.0)	-	20.0 (20.0-24.0)	26.0 (24.0-28.0)	20.0 (18.0-24.0)
mean (SD)	20.0 (4.2)	23.0 (4.8)	-	21.8 (5.4)	26.0 (4.0)	20.9 (4.7)
SpO2, %						
med (IQR)	96.0 (94.0-97.0)	95.0 (94.0-97.0)	-	95.5 (95.0-97.0)	95.5 (94.8-96.2)	96.0 (94.0-97.0)
mean (SD)	95.7 (2.0)	95.2 (2.5)	-	95.8 (2.0)	95.5 (1.5)	95.6 (2.1)
SBP, mmHg						
med (IQR)	123.0 (110.0-134.0)	124.0 (113.0-135.0)	-	119.0 (109.8-132.2)	126.0 (111.5-140.5)	122.0 (110.0-134.0)
mean (SD)	123.8 (17.9)	125.0 (16.1)	-	121.3 (15.9)	126.0 (29.0)	123.6 (17.4)
T, °C						
med (IQR)	37.0 (36.6-37.7)	37.2 (36.7-38.0)	-	36.9 (36.6-37.3)	36.7 (36.4-36.9)	37.0 (36.6-37.7)
mean (SD)	37.2 (0.8)	37.3 (0.9)	-	37.0 (0.7)	36.7 (0.4)	37.2 (0.8)
HR, bpm						
med (IQR)	78.0 (69.0-88.0)	82.0 (73.0-92.0)	-	77.0 (69.8-87.2)	81.5 (74.2-88.8)	78.0 (70.0-89.0)
mean (SD)	78.9 (14.3)	83.2 (16.0)	-	78.5 (12.0)	81.5 (14.5)	79.6 (14.4)
O2, yes/no, %	67.4	78.0	-	87.5	100.0	72.8
O2, L/min						
med (IQR)	2.0 (2.0-4.0)	6.0 (3.0-10.0)	-	3.0 (2.0-4.0)	12.5 (11.2-13.8)	3.0 (2.0-5.0)
mean (SD)	3.2 (2.6)	6.7 (4.4)	-	3.4 (1.5)	12.5 (2.5)	4.0 (3.3)
SpO2/O2, 1/(L/min)						
med (IQR)	47.0 (23.8-49.0)	15.8 (9.4-32.0)	-	31.7 (23.5-47.0)	7.9 (7.2-8.7)	32.0 (19.0-48.5)
mean (SD)	46.2 (27.8)	25.8 (24.0)	-	34.1 (17.6)	7.9 (1.5)	39.5 (26.7)

Table 12: Pathway and population characteristics of the patients in hospital F measured within the first 24 hours of hospital admission. med = median, IQR = inter-quartile range, SD = standard deviation, LOS = length-of-stay, RR = respiratory rate, SpO2 = Peripheral oxygen saturation, SBP = Systolic blood pressure, T = Temperature, HR = Heart rate, O2 = Supplemental oxygen, SpO2/O2 = SpO2-to-O2 ratio.

Appendix F: Evaluation metrics

Appendix F.1: Model discrimination

To quantify the discriminative performance of the different models, we use three different metrics:

- The partial area under the receiver operating characteristic curve (pAUC) between a false positive rate (FPR) of 0 and 0.33.
- The (complete) area under the receiver operating characteristic curve (AUC).
- The area under the precision-recall (PR)-curve (AUCPR).

Appendix F.2: Partial and complete area under the ROC curve

As discussed by McClish,[15] normalization of the partial area under the receiver operating characteristic (ROC) curve (pAUC) is needed. For example, consider an area underneath an ROC curve between false-positive rates (FPRs) 0.7 and 0.9 of 0.18. The maximum value this area could attain is 0.2 and the minimum is 0.16. An area of 0.18 between FPRs 0.3 and 0.5 would have the same maximum of 0.2, but a minimum value of 0.08 (figure 12a). As we want to be able to distinguish between these two cases, the pAUC is normalized such that the minimum value is equal to 0.5 (just like the minimum value of the complete AUC). This normalization is defined as follows:

$$pAUC_{norm} = \frac{1}{2}\left(1 + \frac{pAUC - pAUC_{min}}{pAUC_{max} - pAUC_{min}}\right) \tag{3}$$

In this study, we considered the pAUC between 0 and 0.33 FPR (figure 12b).

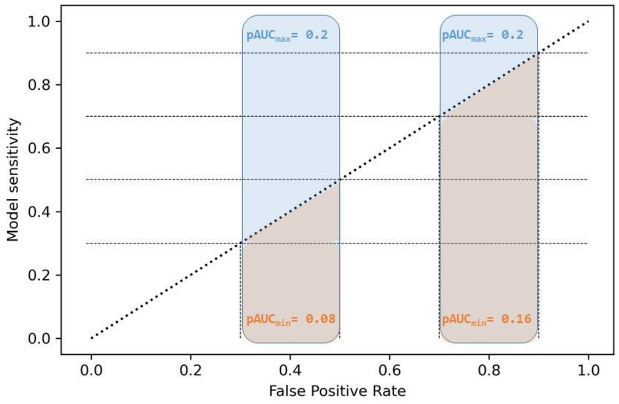

(a) The maximum pAUC between FPR 0.7 and 0.9 and between 0.3 and 0.5 is both 0.2, whereas the minimum areas are 0.16 and 0.08, respectively.

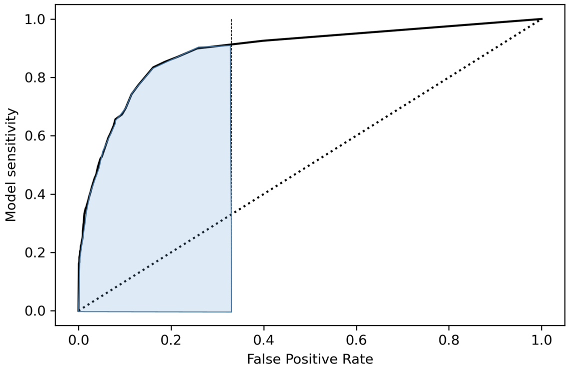

(b) The shaded area denotes the pAUC between FPR 0 and 0.33 (the ROC curve plotted is purely for illustration and not based on study results).

Figure 12: Partial area under the receiver operating characteristic curve (pAUC).

Thus, the pAUC is normalized as follows:

$$pAUC_{norm} = \frac{1}{2}\left(1 + \frac{pAUC - 0.05445}{0.33 - 0.05445}\right) \quad (4)$$

The normalized pAUC was implemented using the 'roc_auc_score' function offered by scikit-learn in Python,[3] setting 'max_fpr' to 0.33. For the complete area under the ROC curve (AUC), we used the same function with default parameters. For both pAUC and AUC, we calculated the bootstrap percentile confidence intervals as described by Qin et al.,[16] using 1000 bootstrap replications, each stratified for positive and negative samples.

Appendix F.3: Area under the precision-recall (PR)-curve

The area under the precision-recall (PR) curve (AUCPR) is a useful performance metric for imbalanced data settings where finding the true positives is important. In this study, a true positive outweights a false positive and we are dealing with imbalanced data. In their work on the AUCPR, Boyd and colleagues[17] recommend to use the average precision (AP) as a point estimate for the AUCPR. Using the AP is preferred over, for instance, computing the AUCPR with the trapezoidal rule, as calculating the AP does not require interpolation that could lead to too optimistic estimates. The AP summarizes a PR-curve as the weighted mean of precisions (PPVs) achieved at each model threshold, with the increase in recall from the previous

threshold used as the weight. It is defined as follows:

$$AP = \sum_n (R_n - R_{n-1})P_n \tag{5}$$

where R_n and P_n are the recall (or sensitivity) and precision (or PPV) at the nth model threshold. To calculate the AP, we used the 'average_precision_score' function offered by scikit-learn in Python.[3] To calculate uncertainty around this metric, we used the binomial confidence interval (CI) as it showed good coverage for 95% CIs in previous work[17] and does not require any additional calculations compared to, for instance, a bootstrapping method. The binomial 95% CI is defined as follows:

$$AP \pm 1.96\sqrt{\frac{AP(1-AP)}{n}} \tag{6}$$

where n is the number of positive samples. The number of positive samples specifies the maximum number of unique recall (sensitivity) values.

Appendix F.4: Comparing performances

Because we want to compare the performances of the new model to the performance of existing EWSs, we need to compare these models based on different performance metrics. Therefore, we conducted a test statistic (Z) described by McClish[15] to compare each of the three area-based performance metrics for model discrimination (pAUC, AUPRC and AUC), which is described as follows:

$$Z = \frac{A_1 - A_2}{\sqrt{Var(A_1 - A_2)}} \tag{7}$$

where A_1 and A_2 are the two areas to be compared, and $Var(A_1 - A_2)$ is the variance of the differences between the areas, approximated from the bootstrap differences in a 1000 sample stratified bootstrapping procedure. Z is compared to the normal distribution to determine the corresponding P-value.

Appendix F.5: Net Benefit

The net benefit (NB) is a metric that can be used for evaluating the clinical implications of medical prediction models. The metric is defined by Vickers and colleagues[18] as follows:

$$NB = \text{benefit} - \text{harm} \times \text{exchange rate} \tag{8}$$

where the 'benefit' is defined as the number of true positives (as a fraction of the total observations) and the 'harm' as the number of false positives (also as a fraction of the total observations). The 'exchange rate' is a clinical judgement of the relative values of benefits (finding cases) and harms (causing false alarms). It can be derived by asking what is the maximum number of triggers (or alarms) one is willing to invest to find one case. For example, a physician may argue: to find one patient who is deteriorating, no more than 20 patients should be checked. This implies that we want to 'weight' finding a true case as nineteen times more important than avoiding one false alarm. Assuming a perfectly calibrated model, the model threshold ($\mathbb{P}_{deterioration}$) corresponding to this exchange rate is 5%.

In this study, we considered early detection of a deteriorating patient as at least four times more important than preventing an unnecessary response (false alarm) and therefore, we plotted the DCA results up to 0.2 deterioration probability. The NB is normalized as the fraction of the maximum NB (i.e. the number of true positives as a fraction of the total observations). So, the normalized NB is be calculated as follows:

$$\text{Standardized NB} = \frac{\frac{TP}{N} - \frac{FP}{N} \times \frac{\mathbb{P}_{deterioration}}{(1-\mathbb{P}_{deterioration})}}{\frac{TP}{N}}. \tag{9}$$

Here, N is the total number of samples and $\mathbb{P}_{deterioration}$ the probability threshold used to trigger a certain action. In the context of early warning, this could be either an urgent clinical response or emergency clinical response.

Appendix F.6: Model calibration

To quantify the calibration performance of medical prediction models, Van Calster and colleagues[19] distinguish increasingly stringent levels of calibration. We evaluated calibration in the weak and moderate sense. To evaluate calibration in the weak

sense, we calculated the calibration intercept and calibration slope (N.B., these are not the intercept and slopes of calibration curves in the probability domain). They were first introduced by Cox in 1958,[20] and result from regressing the predictions in the log-odds domain to the observed posteriors. The calibration slope evaluates the spread of the estimated risks and has a target value of 1. A slope < 1 suggests that estimated risks are too extreme, while a slope > 1 suggests that risk estimates are too moderate. The calibration intercept is an assessment of calibration-in-the-large and has a target value of 0. Negative values suggest overestimation, whereas positive values suggest underestimation. We evaluated calibration in the moderate sense by plotting smoothed calibration curves.

Appendix G: Dynamic model updating

Appendix G.1: Different model updating strategies

For model updating, we examined different strategies consisting of model refitting and different combinations of re-fitting and recalibration. In each of these strategies, models are updated each month from August 2020 until May 2021, by either fitting the model based all data available up to that moment (i.e., the 'Refit strategy', figure 13a), or by splitting the available data in one part used to refit the model and another part to fit an extra mapping function (i.e. a calibrator) using isotonic regression (IR)[21] that is used to re-map the predictions of fitted model. We experimented with three different strategies of splitting the training set into a training and calibration set:

- **Isotonic regression (IR):** Each month, one calibrator is fitted using IR based on the data collected from the four most recent months of all hospitals. The remaining data is used to refit the model and the calibrator, resulting in one 'model-calibrator pair'. The calibrator is used to re-map the predictions of the fitted model (figure 13b).
- **Hospital-specific isotonic regression (HS-IR):** Each month, a separate calibrator is fitted for each hospital using IR based on all the data collected in that specific hospital and a model is fitted using the remaining data, resulting in six 'model-calibrator pairs'. The calibrators are used to re-map the predictions of the corresponding fitted models (figure 13c).
- **Moving, hospital-specific isotonic regression (Moving HS-IR):** Each month, a separate calibrator is fitted for each hospital using IR based on data collected in the four most recent months from that specific hospital and a models is fitted using the remaining data, resulting in six 'model-calibrator pairs'. The calibrators are used to re-map the predictions of the paired fitted models (figure 13d).

We applied each of the strategies and evaluated these by assessing the overall (i.e., based on the full test set) and hospital-specific calibration performances (in the weak and moderate sense).

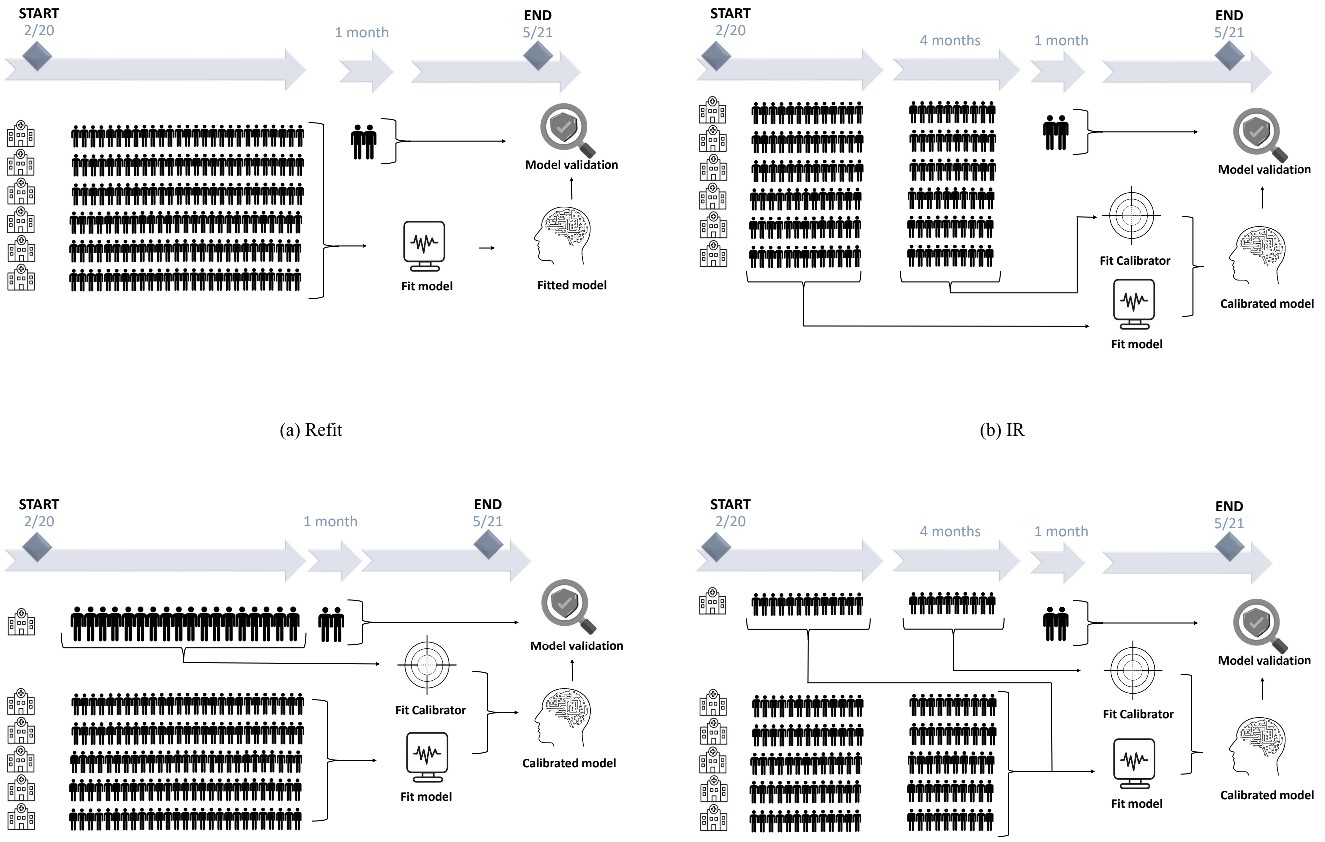

(a) Refit

(b) IR

(c) HS-IR

(d) Moving HS-IR

Figure 13: Schematic overview of how models would predict deterioration among patients admitted in October, 2020, according to the different dynamic model updating strategies. These procedures are repeated each month, from August 2020 until May 2021.

Appendix G.2: Results

Figure 14 shows the overall calibration intercepts and slopes (95% CI), as well as the corresponding partial areas under the receiver operating characteristic curves (pAUCs) yielded by the different model updating strategies for the LR and RF model. Figure 15 shows the corresponding flexible calibration curves. Figures 16 - 27 show the hospital-specific calibration intercepts, slopes, pAUCs and the corresponding flexible calibration curves yielded by the different model updating strategies applied to the RF and LR models. For all figures showing flexible calibration curves, we zoom in to the domain between 0 and 0.2 probability, because the majority of the predictions are in this range.

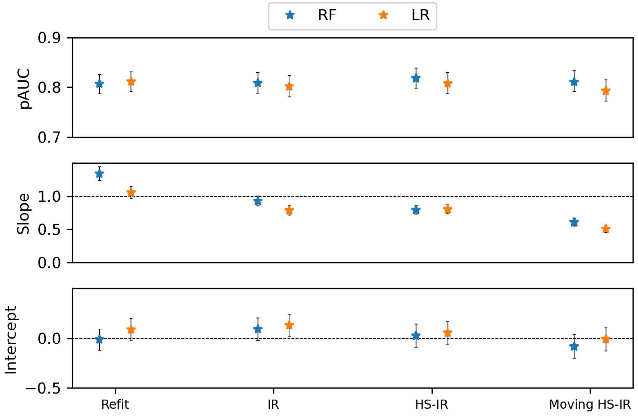

Figure 14: Overall pAUCs (based on full test set), calibration intercepts and slopes yielded by the different dynamic model updating strategies. pAUC=partial area under the receiver operating characteristic curve, RF=random forest, LR=logistic regression.

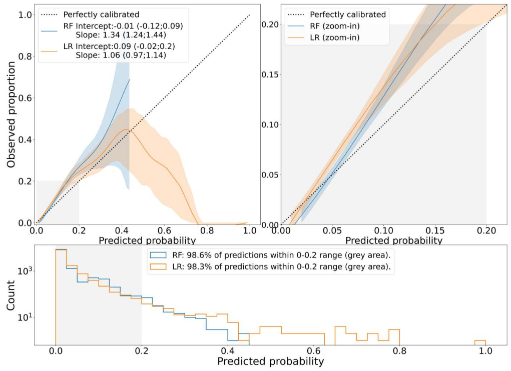

(a) Refit

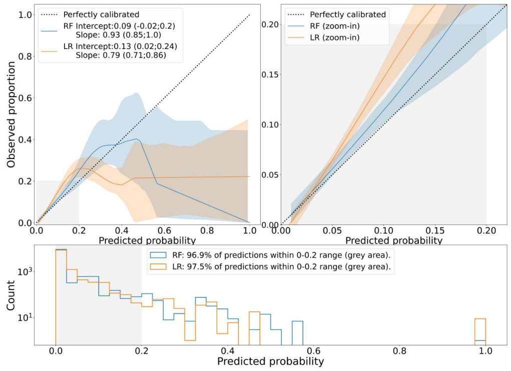

(b) IR

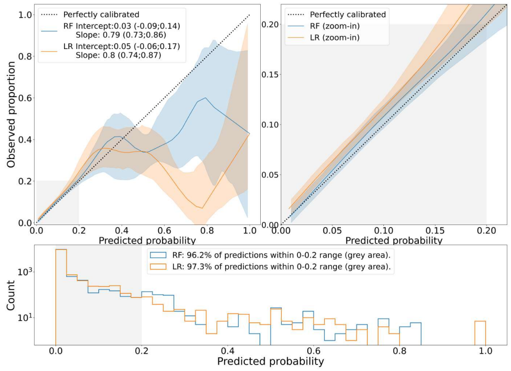

(c) HS-IR

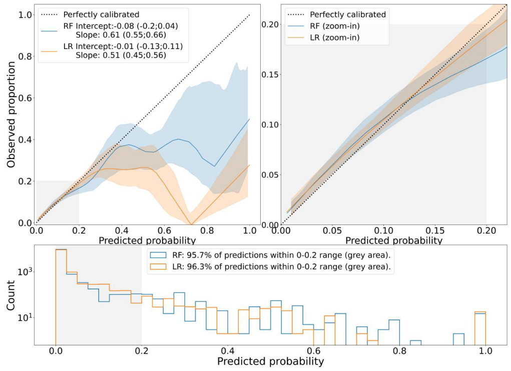

(d) Moving HS-IR

Figure 15: Overall model calibration curves resulting from the different dynamic model updating strategies. (i) Smoothed flexible calibration curves. (ii) Zoom-in of the calibration curve in the 0-0.2 probability range (grey area). Shaded areas around the curves represent the 95% CIs. (iii) Histogram of the predictions (log scale).

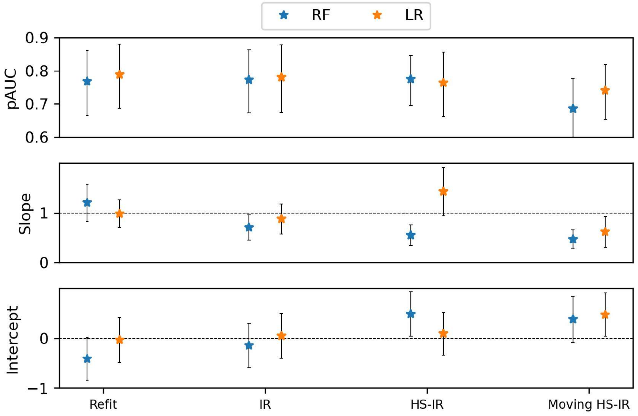

Figure 16: pAUCs, calibration intercepts and slopes yielded by the different dynamic model updating strategies in hospital A. pAUC=partial area under the receiver operating characteristic curve, RF=random forest, LR=logistic regression.

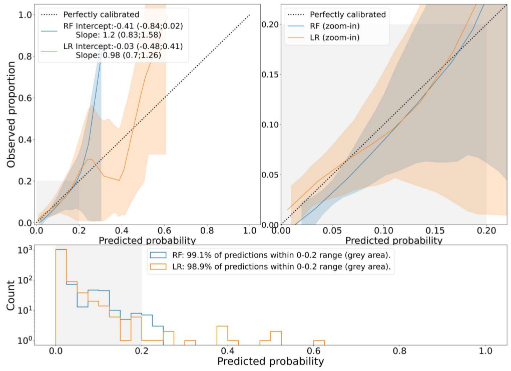

(a) Refit

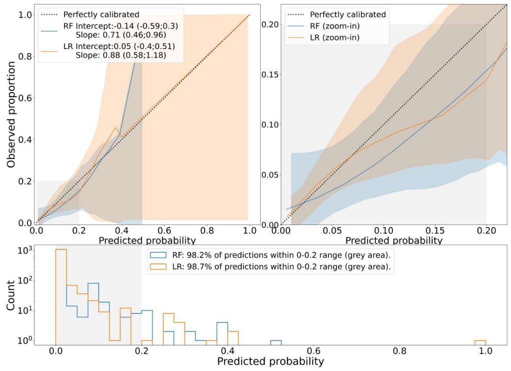

(b) IR

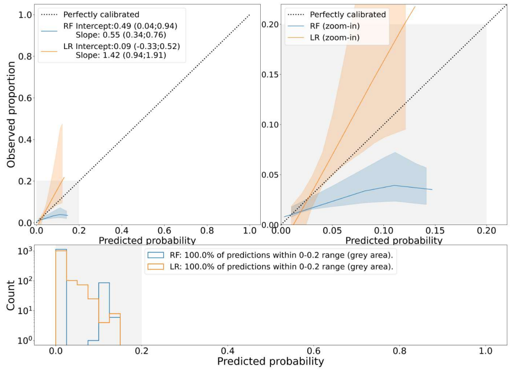

(c) HS-IR

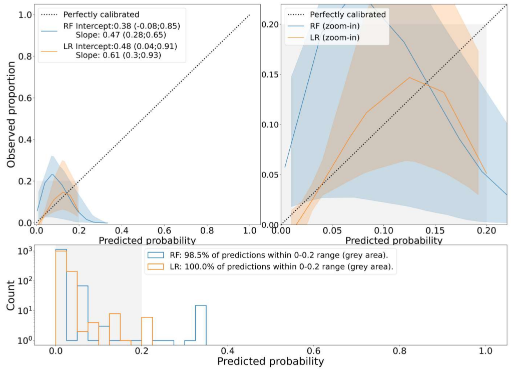

(d) Moving HS-IR

Figure 17: Model calibration curves yielded in hospital A by the different dynamic model updating strategies. (i) Smoothed flexible calibration curves. (ii) Zoom-in of the calibration curve in the 0-0.2 probability range (grey area). Shaded areas around the curves represent the 95% CIs. (iii) Histogram of the predictions (log scale).

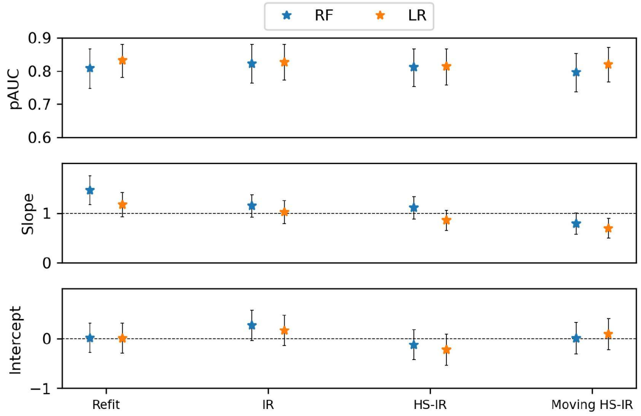

Figure 18: pAUCs, calibration intercepts and slopes yielded by the different dynamic model updating strategies in hospital B. pAUC=partial area under the receiver operating characteristic curve, RF=random forest, LR=logistic regression.

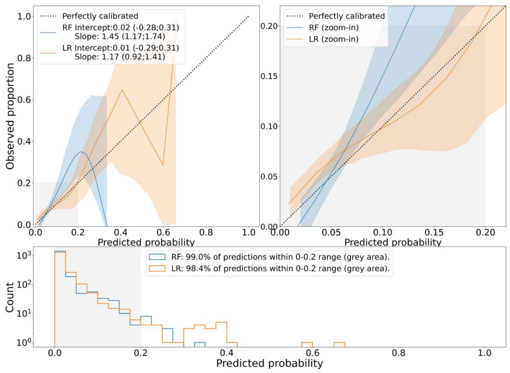

(a) Refit

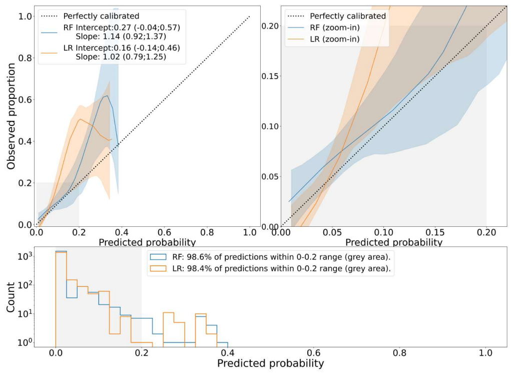

(b) IR

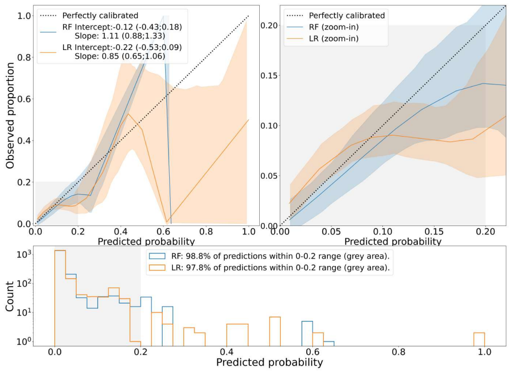

(c) HS-IR

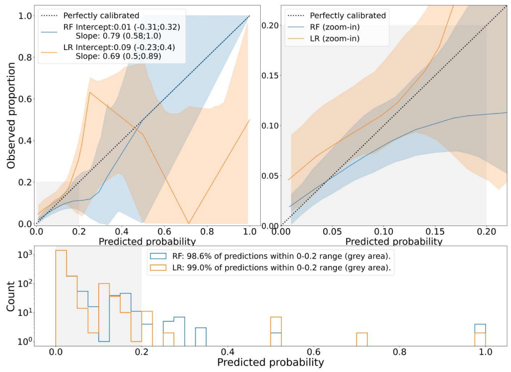

(d) Moving HS-IR

Figure 19: Model calibration curves yielded in hospital B by the different dynamic model updating strategies. (i) Smoothed flexible calibration curves. (ii) Zoom-in of the calibration curve in the 0-0.2 probability range (grey area). Shaded areas around the curves represent the 95% CIs. (iii) Histogram of the predictions (log scale).

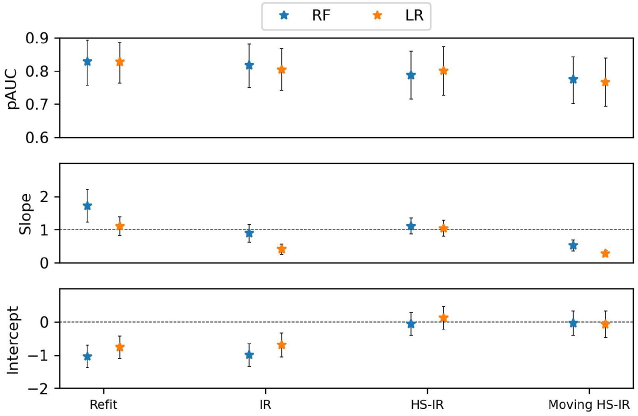

Figure 20: pAUCs, calibration intercepts and slopes yielded by the different dynamic model updating strategies in hospital C. pAUC=partial area under the receiver operating characteristic curve, RF=random forest, LR=logistic regression.

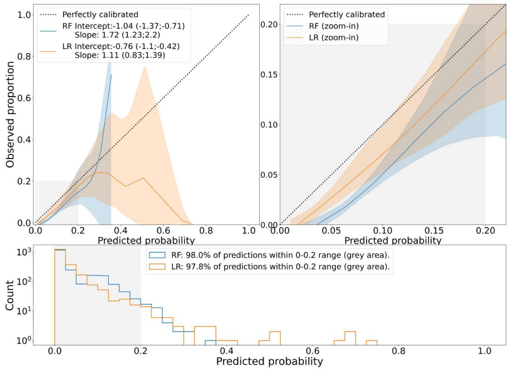

(a) Refit

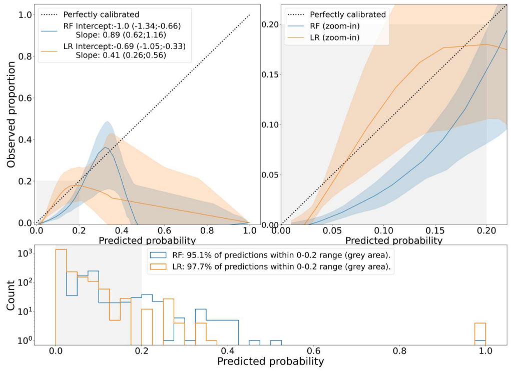

(b) IR

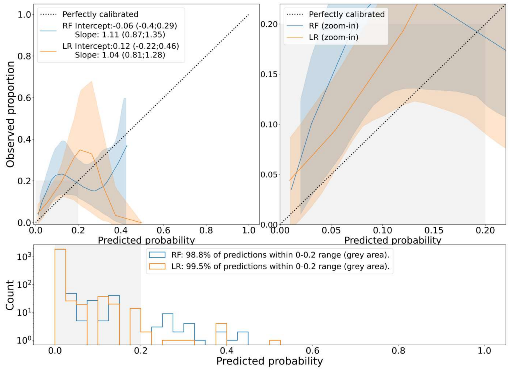

(c) HS-IR

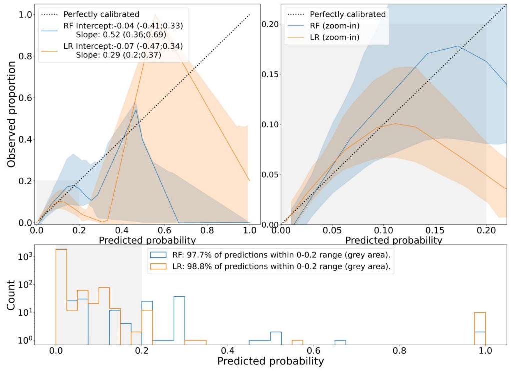

(d) Moving HS-IR

Figure 21: Model calibration curves yielded in hospital C by the different dynamic model updating strategies. (i) Smoothed flexible calibration curves. (ii) Zoom-in of the calibration curve in the 0-0.2 probability range (grey area). Shaded areas around the curves represent the 95% CIs. (iii) Histogram of the predictions (log scale).

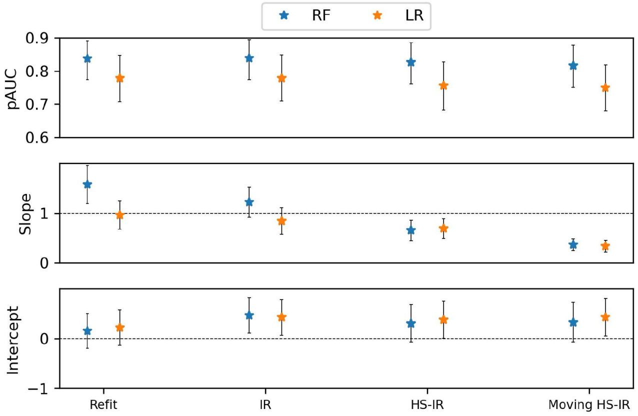

Figure 22: pAUCs, calibration intercepts and slopes yielded by the different dynamic model updating strategies in hospital D. pAUC=partial area under the receiver operating characteristic curve, RF=random forest, LR=logistic regression.

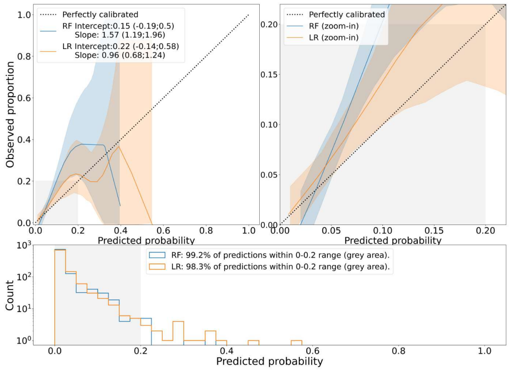

(a) Refit

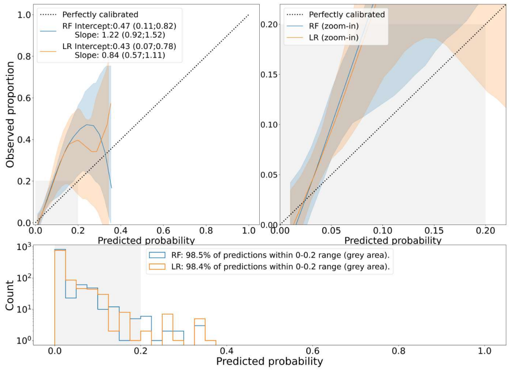

(b) IR

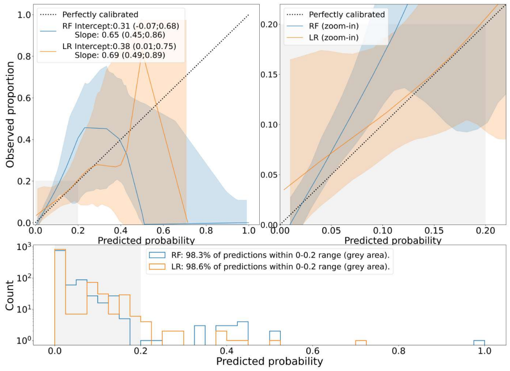

(c) HS-IR

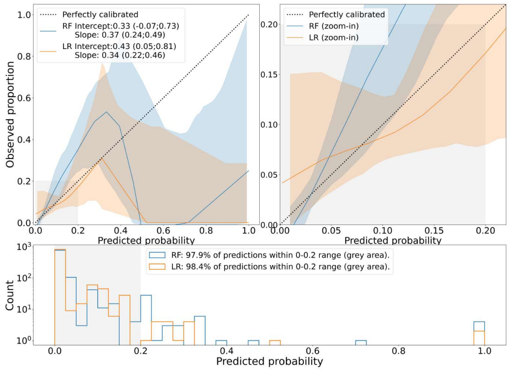

(d) Moving HS-IR

Figure 23: Model calibration curves yielded in hospital D by the different dynamic model updating strategies. (i) Smoothed flexible calibration curves. (ii) Zoom-in of the calibration curve in the 0-0.2 probability range (grey area). Shaded areas around the curves represent the 95% CIs. (iii) Histogram of the predictions (log scale).

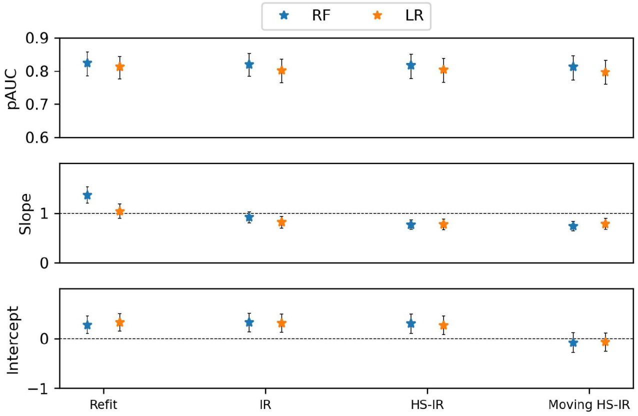

Figure 24: pAUCs, calibration intercepts and slopes yielded by the different dynamic model updating strategies in hospital E. pAUC=partial area under the receiver operating characteristic curve, RF=random forest, LR=logistic regression.

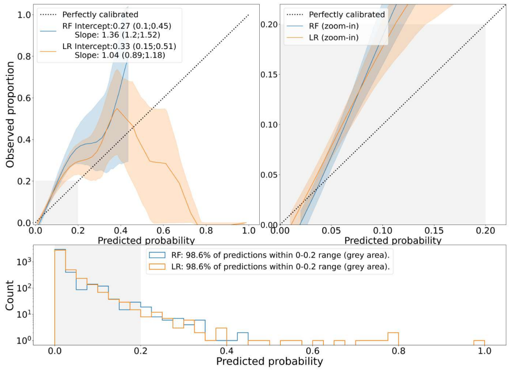

(a) Refit

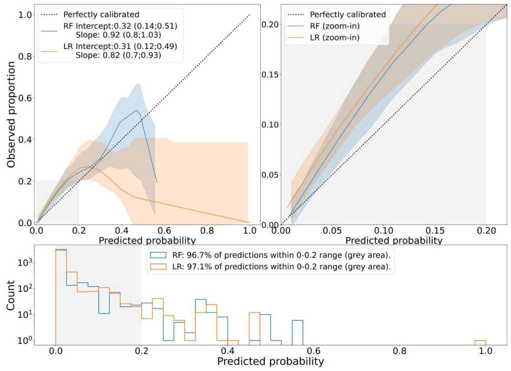

(b) IR

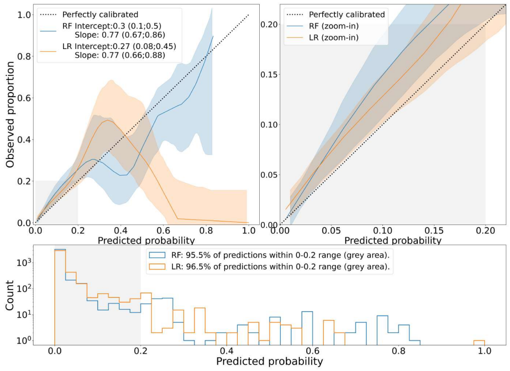

(c) HS-IR

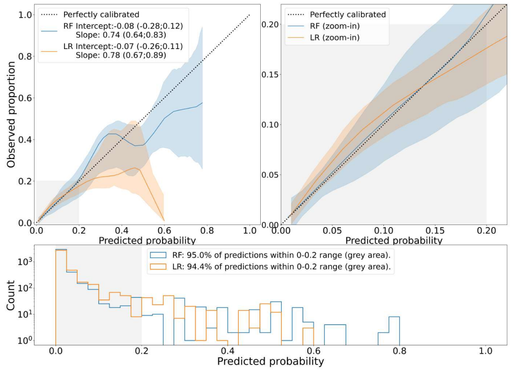

(d) Moving HS-IR

Figure 25: Model calibration curves yielded in hospital E by the different dynamic model updating strategies. (i) Smoothed flexible calibration curves. (ii) Zoom-in of the calibration curve in the 0-0.2 probability range (grey area). Shaded areas around the curves represent the 95% CIs. (iii) Histogram of the predictions (log scale).

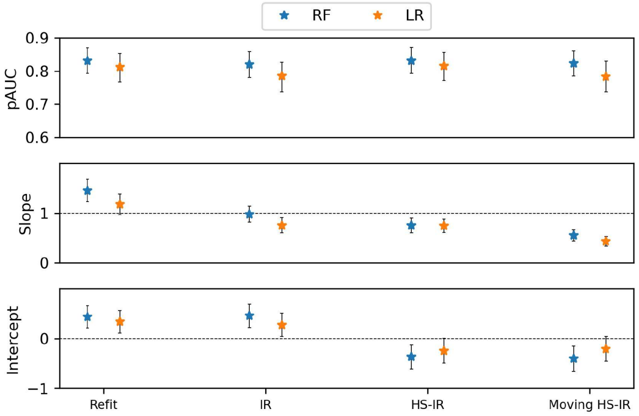

Figure 26: pAUCs, calibration intercepts and slopes yielded by the different dynamic model updating strategies in hospital F. pAUC=partial area under the receiver operating characteristic curve, RF=random forest, LR=logistic regression.

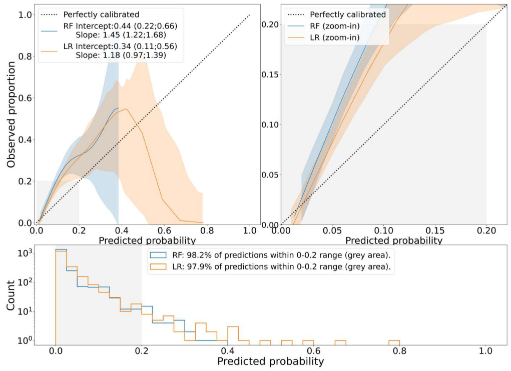

(a) Refit

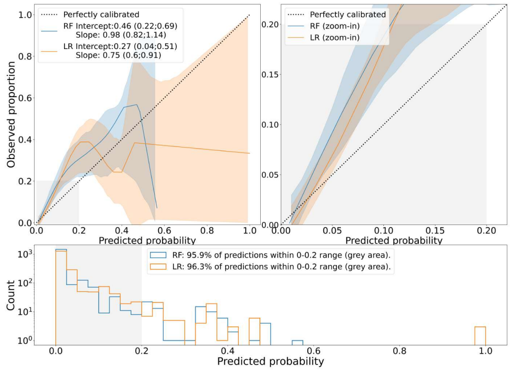

(b) IR

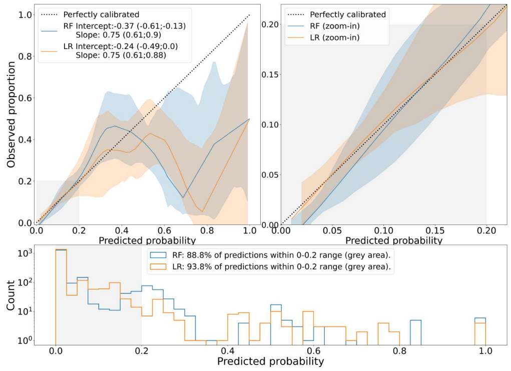

(c) HS-IR

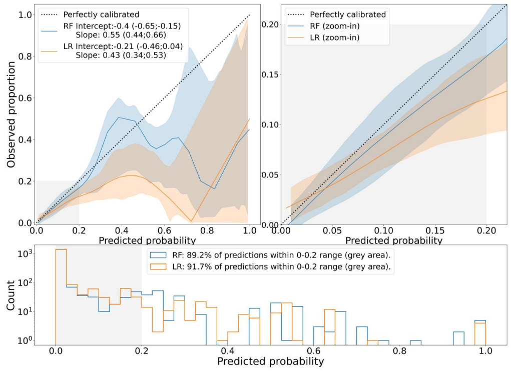

(d) Moving HS-IR

Figure 27: Model calibration curves yielded in hospital F by the different dynamic model updating strategies. (i) Smoothed flexible calibration curves. (ii) Zoom-in of the calibration curve in the 0-0.2 probability range (grey area). Shaded areas around the curves represent the 95% CIs. (iii) Histogram of the predictions (log scale).

Appendix G.3: Evaluation

The discriminative performance (pAUCs) varies marginally for the different strategies (figure 14), whereas the model calibration shows notable differences. According to the hierarchy described by Van Calster and colleagues,[19] calibration in the moderate sense (i.e. the calibration curves) is a more stringent level of calibration than calibration in the weak sense (i.e. the calibration intercept and slope). Therefore, we put most weight in the resulting flexible calibration curves when evaluating the model calibration resulting from the different strategies. In these calibration curves, we focus mainly on the zoomed-in region (between probability 0 and 0.2) that covers the majority of the predictions. Also, we put more weight in the overall than the hospital-specific calibration curves, as they are based on more datapoints and therefore paint a more reliable picture of the model calibration. Finally, as the RF model yielded higher net benefit than the LR model, we put most weight in the calibration of the RF models.

The HS-IR and moving HS-IR strategies both yielded clearly better overall calibration curves compared to the Refit and the IR strategy (figure 15), but mutually show comparable deviations from the diagonal. Among the different hospitals, it varies whether HS-IR or moving HS-IR shows a better calibration curve. For example, the moving HS-IR strategy yielded the best calibration curve in hospital C (figure 21d), whereas in hospital B, the HS-IR strategy yielded the best calibration curve (figure 19c). Thus, solely based on the calibration curves, it is hard to tell which strategy resulted in the best model calibration. We used the overall calibration in the weak sense (figure 14 as a tie breaker. The overall calibration intercepts are very similar, but the overall calibration slopes are closer to target value 1 for the HS-IR strategy. Therefore, we considered the HS-IR strategy as optimal in this study (and show these results of in the main text).

Appendix H: Retrospective validation

We validated the models retrospectively in a 'leave-one-hospital-out' cross-validation procedure. That is, in each iteration, five hospitals formed the development set and the remaining hospital the test set. We repeated this process until each hospital formed the test set once, resulting in six RF and six LR models.

The RF models showed significantly improved ($P<0.001$) discriminative performance compared to the NEWS, with an overall (based on the full test set) pAUC of 0.78 [0.77 to 0.80] versus 0.72 [0.70 to 0.74] (figure 28a). The LR models performed comparable to the RF models (pAUC=0.79 [0.78 to 0.81]) and the MEWS performed worse than all other models (pAUC=0.68 [0.66 to 0.69]). Figure 28b shows the pAUCs yielded by RF and LR models and the NEWS in the individual hospitals. The RF and LR models show comparable overall performance in the decision curve analysis (DCA) and both show a clear improvement in net benefit (NB) compared to the existing EWSs (figure 28c). Both models yielded good calibration, with calibration curves close to the diagonal (figure 28d).

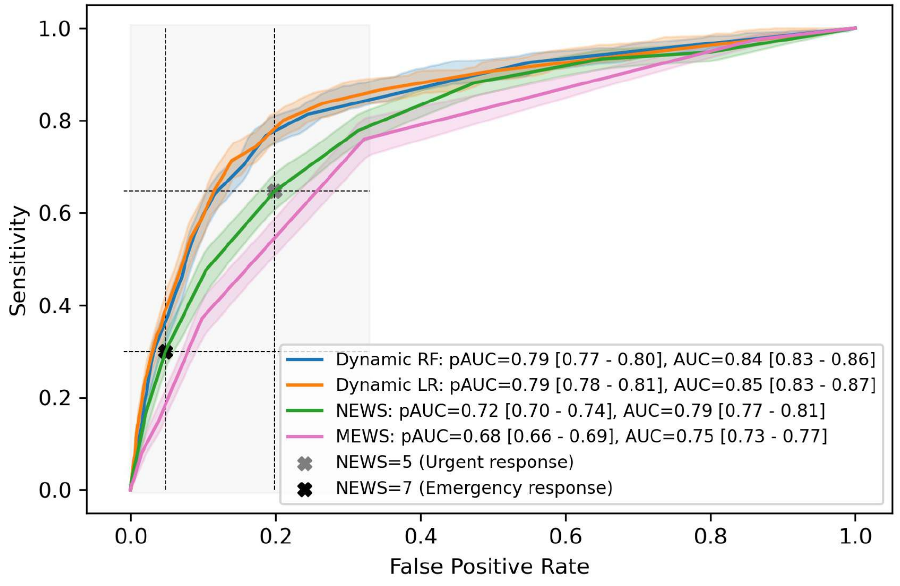

(a) Overall receiver operating characteristic curves for the RF and LR models and the NEWS. We placed two landmarks for a NEW score of 5 and 7, i.e. the recommended trigger thresholds for an urgent and emergency response. We calculated both the pAUC between a false positive rate of 0 and 0.33 (grey area) and the complete AUC.

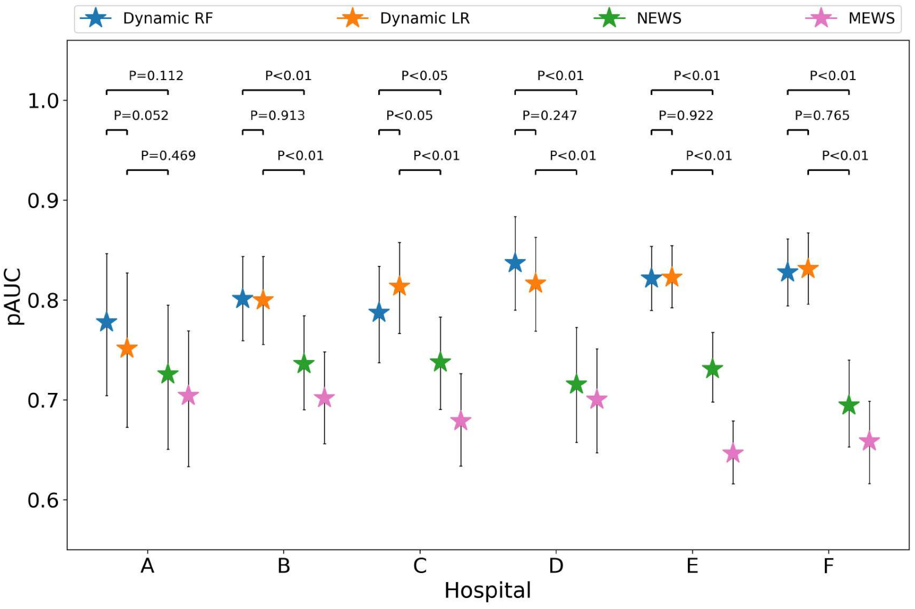

(b) Hospital-specific pAUCs. P values are shown resulting from significance tests for the difference in pAUC between the RF models and NEWS (upper bar), between the RF and LR models (middle bar) and between the LR models and NEWS (lower bar).

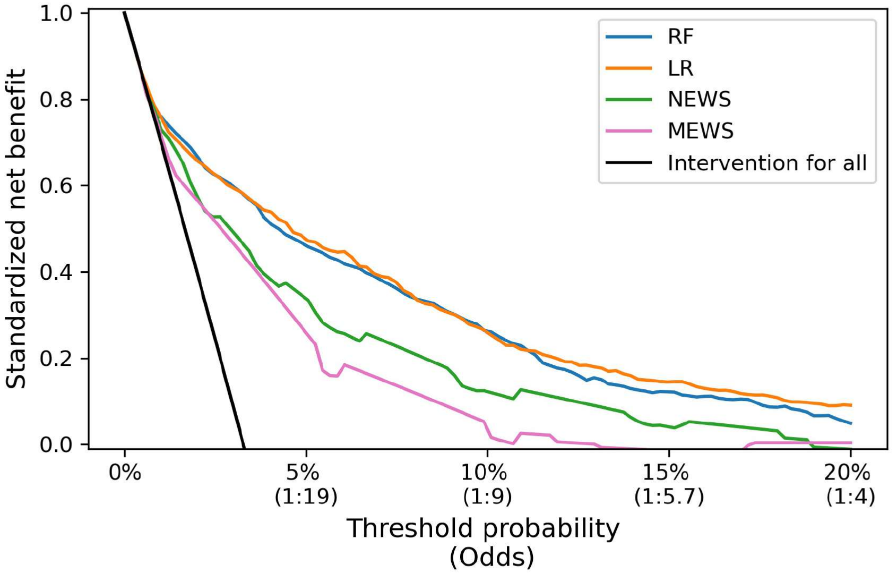

(c) Overall decision curve analysis results. The standardized net benefit is plotted over a range of clinically relevant probability thresholds with corresponding odds. The 'Intervention for all' line indicates the net benefit if a (urgent or emergency) response would always be triggered.

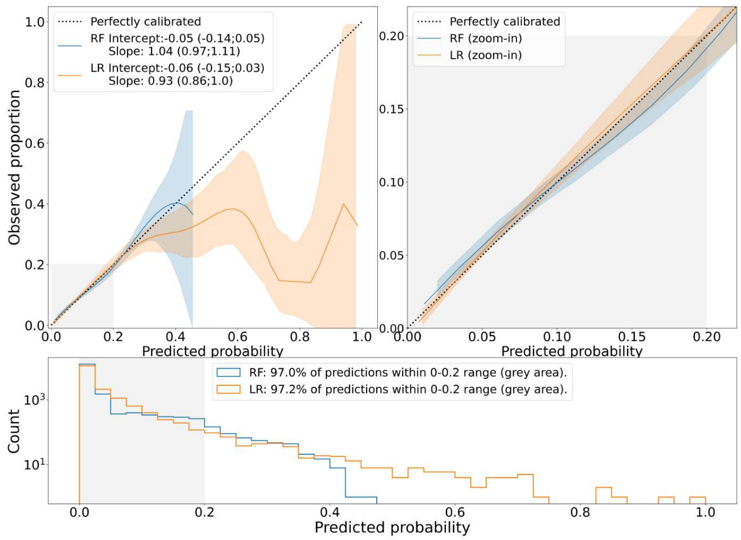

(d) Overall model calibration of the RF model and LR model. (top left) Smoothed flexible calibration curves. (top right) Zoom-in of the calibration curve in the 0-0.2 probability range (grey area). (bottom) Histogram of the predictions (log scale). Shaded areas around each point in the calibration curves (before smoothing) represent the 95% bootstrap percentile CIs (with 1000 bootstrap replications stratified for positive and negative samples). The smooth curves including CIs were estimated by locally weighted scatterplot smoothing (see https://github.com/jimmsmit/COVID-19_EWS for the implementation).

Figure 28: Results of the retrospective (Leave-one-hospital-out) validation procedure.
RF=Random Forest, LR=Logistic Regression, MEWS=Modified Early Warning Score, NEWS=National Early Warning Score

Appendix I: Sensitivity analysis

Appendix I.1: Predictive modeling vs aggregate-weighted early warning scores

To examine the added value of predictive modeling compared with aggregate-weighted early warning scores, we repeated the temporal validation (dynamic strategy) using a RF and LR model fitted only with the predictors required to calculate the MEWS and the NEWS (see table 13) and compared the performance with the corresponding aggregate-weighted EWSs. Figure 29 shows the discriminative performance of the aggregate-weighted scores (i.e. the MEWS and NEWS) and the RF and LR models. The RF and LR models fitted with the MEWS predictor set outperformed the MEWS, while for the NEWS predictor set, the RF and LR models only show marginal improvement compared to the NEWS.

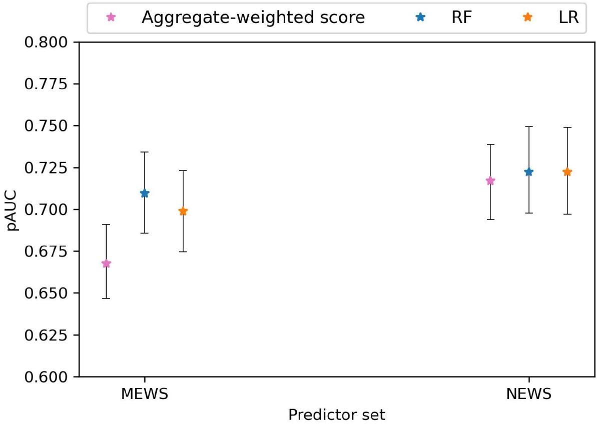

Figure 29: Overall (based on complete test set) pAUCs yielded by the Modified Early Warning Score, the National Early Warning Score and the dynamic RF and LR models fitted using only the predictors required to calculate these aggregate-weighted scores, in the temporal validation.

Appendix I.2: Influence of included predictors on model performance

To further examine the performance of models fitted with different predictor subsets, we repeated the temporal validation (dynamic strategy) with RF and LR models fitted with seven different predictor sets (table 13):

- predictors with an entry density (ED) ≥ 0.5 (the models presented in the main text)
- predictors required to calculate the MEWS
- predictors required to calculate the NEWS
- predictors with an ED ≥ 0.4
- predictors with an ED ≥ 0.3
- top five predictors based on the mean SHAP magnitude
- predictors required to calculate the NEWS, supplemented with the SpO_2/O_2

For all these models, we performed the temporal validation including monthly model updating as described in the main text.

Figure 30 shows the pAUCs, calibration intercepts and slopes yielded by the different models. Figure 31 shows all the flexible calibration curves resulting from the different models. The models fitted with the predictors required to calculate the MEWS and NEWS yielded significantly lower pAUCs and show bad calibration curves compared to the other models. The models fitted with more predictors (ED $\geq$ 0.4 and 0.3) show little difference in pAUC and calibration curves compared to the original models (ED ≥ 0.5), only the calibration curve of the ED ≥ 0.4 model shows more deviation from the diagonal. The models fitted with fewer predictors (top 5) and the models fitted with the NEWS predictor supplemented with SpO_2/O_2 show little difference in pAUC and in the calibration curves compared to the original models (ED $\geq$ 0.5).

**Predictor set**	**Included predictors**	**pAUC**	**Calibration intercept**	**Calibration slope**
Entry density $\geq$ 0.5 (main text)	RR, SpO2, SBP, Temp, HR, O2 (yes/no), AVPU, Age, Sex, LOS, O2 (L/min), SpO_2/O_2, ΔRR, ΔSpO2, ΔSBP, ΔTemp, ΔHR, $\Delta SpO_2/O_2$	RF: 0.82 [0.80;0.84]<br>LR: 0.81 [0.79;0.83]	RF: 0.03 [-0.09;0.14]<br>LR: 0.05 [-0.06;0.17]	RF: 0.79 [0.73;0.86]<br>LR: 0.80 [0.74;0.87]
Required to calculate MEWS	RR, SBP, Temp, HR, AVPU	RF: 0.71 [0.69;0.73]<br>LR: 0.70 [0.67;0.72]	RF: 0.12 [0.01;0.23]<br>LR: 0.49 [0.38;0.61]	RF: 0.60 [0.53;0.68]<br>LR: 0.39 [0.34;0.45]
Required to calculate NEWS	RR, SpO2, SBP, Temp, HR, O2 (yes/no), AVPU	RF: 0.72 [0.70;0.75]<br>LR: 0.72 [0.70;0.75]	RF: 0.14 [0.03;0.25]<br>LR: 0.43 [0.31;0.54]	RF: 0.55 [0.48;0.62]<br>LR: 0.43 [0.38;0.49]
Entry density $\geq$ 0.4	RR, SpO2, SBP, Temp, HR, O2 (yes/no), AVPU, Age, Sex, LOS, O2 (L/min), SpO_2/O_2, ΔRR, ΔSpO2, ΔSBP, ΔTemp, ΔHR, $\Delta SpO_2/O_2$, Haemoglobin, WBC, Sodium, Potassium, Creatinine,	RF: 0.81 [0.79;0.83]<br>LR: 0.80 [0.78;0.82]	RF: -0.03 [-0.15;0.08]<br>LR: 0.05 [-0.06;0.16]	RF: 0.78 [0.72;0.84]<br>LR: 0.82 [0.75;0.89]
Entry density $\geq$ 0.3	RR, SpO2, SBP, Temp, HR, O2 (yes/no), AVPU, Age, Sex, LOS, O2 (L/min), SpO_2/O_2, ΔRR, ΔSpO2, ΔSBP, ΔTemp, ΔHR, $\Delta SpO_2/O_2$, Haemoglobin, WBC, Sodium, Potassium, Creatinine, Haematocrit, Platelet count, ALAT, ASAT, LD, Urea, CRP, RDW	RF: 0.82 [0.80;0.84]<br>LR: 0.79 [0.77;0.81]	RF: -0.02 [-0.14;0.09]<br>LR: 0.02 [-0.09;0.14]	RF: 0.74 [0.67;0.80]<br>LR: 0.78 [0.72;0.85]
Top 5 predictors	SpO_2/O_2, RR, Temp, LOS, O2(L/min)	RF: 0.82 [0.80;0.84]<br>LR: 0.81 [0.79;0.83]	RF: 0.01 [-0.11;0.12]<br>LR: 0.13 [0.02;0.25]	RF: 0.71 [0.65;0.77]<br>LR: 0.70 [0.64;0.76]
NEWS + SpO_2/O_2	RR, SpO2, SBP, Temp, HR, O2 (yes/no), AVPU, SpO_2/O_2	RF: 0.82 [0.80;0.84]<br>LR: 0.79 [0.77;0.81]	RF: 0.02 [-0.10;0.13]<br>LR: 0.17 [0.05;0.28]	RF: 0.78 [0.71;0.84]<br>LR: 0.70 [0.64;0.77]

Table 13: Model performance for different included predictor sets.
pAUC= partial area under the receiver operating characteristic curve, RR= respiratory rate, SBP= Systolic blood pressure, Temp= Temperature, HR= Heart rate, WBC= White blood cell count, LD= Lactate dehydrogenase, CRP= C reactive protein, RDW= Red cell distribution width, LOS= ward length-of-stay

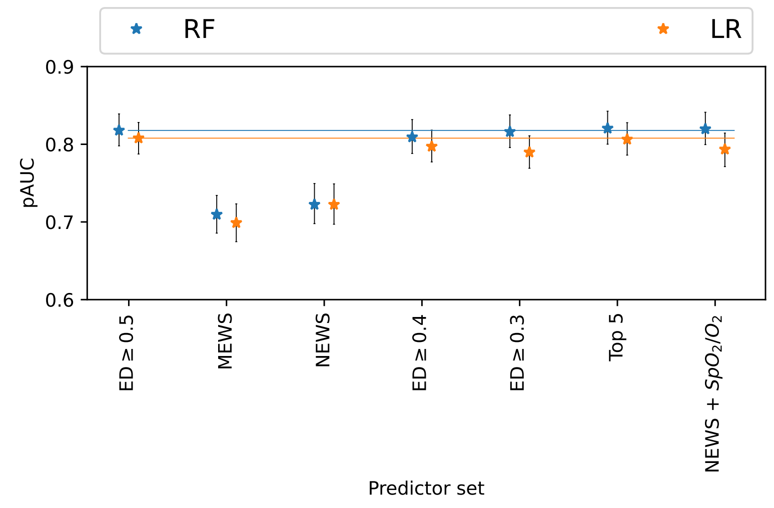

(a) pAUC

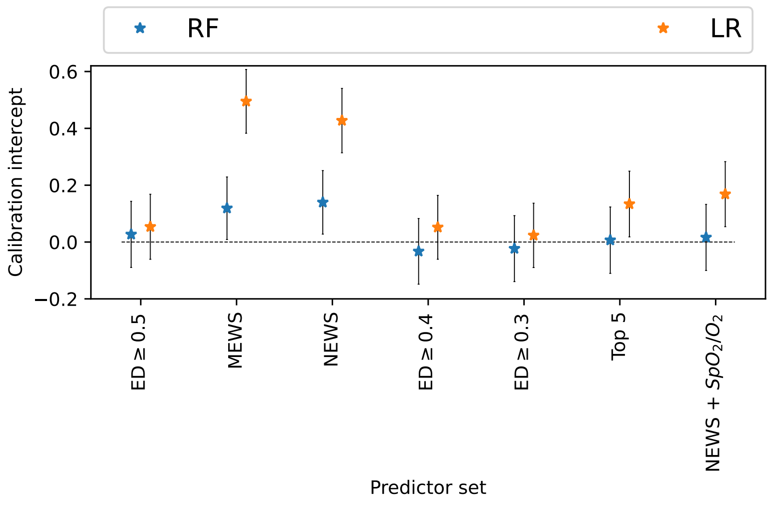

(b) Calibration intercept

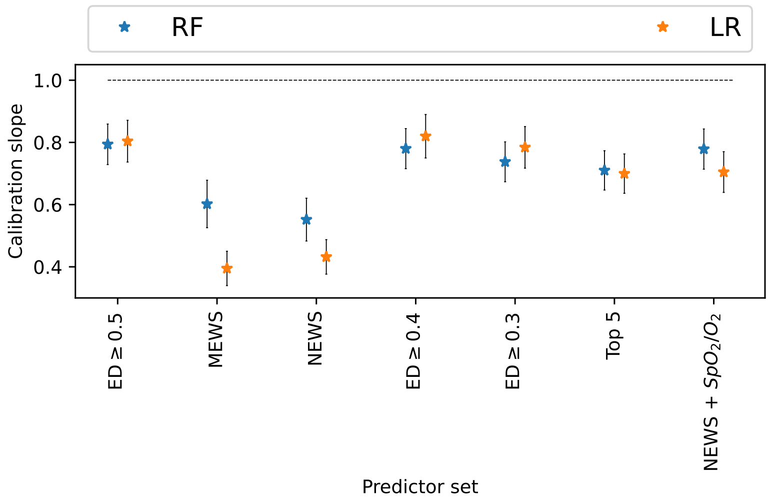

(c) Calibration slope

Figure 30: The discrimination (pAUC) and calibration in the weak sense (intercepts and slopes) yielded by the the LR and RF models fitted with the different predictor sets: ED $\geq$ 0.5 (main text), ED $\geq$ 0.4, ED $\geq$ 0.3, MEWS, NEWS and Top 5.
ED=entry density, MEWS=Modified Early Warning Score, NEWS=National Early Warning Score.

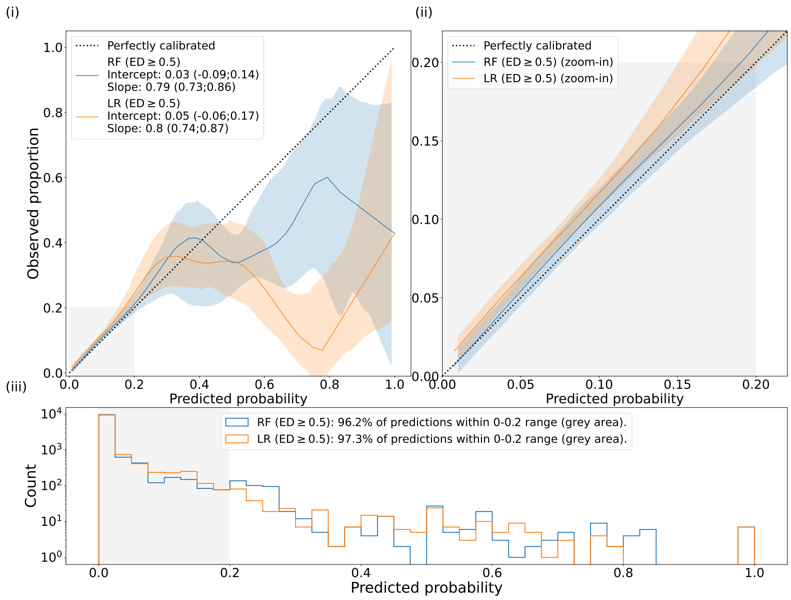

(a) ED $\geq$ 0.5 (main text)

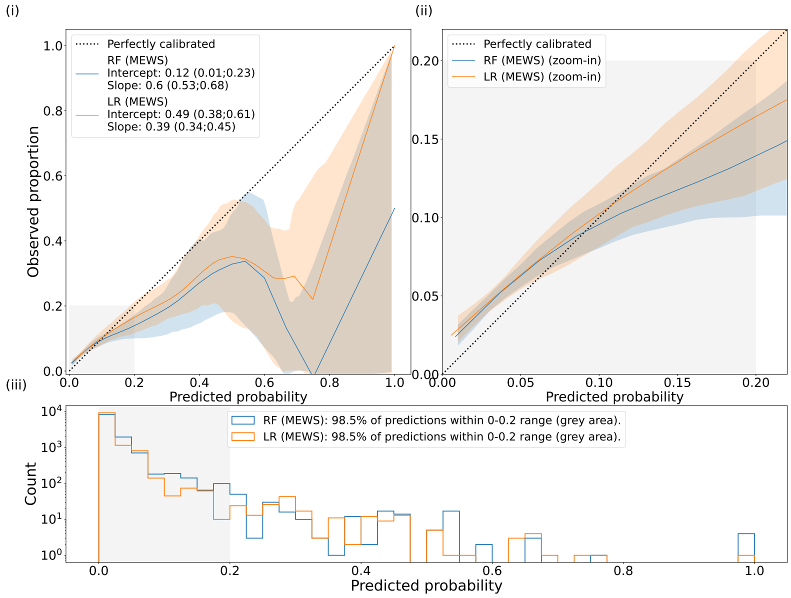

(b) MEWS

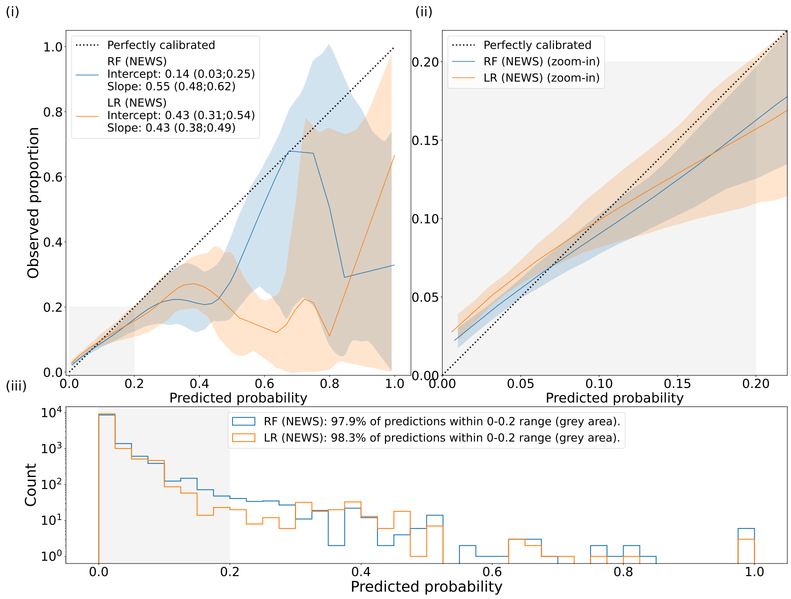

(c) NEWS

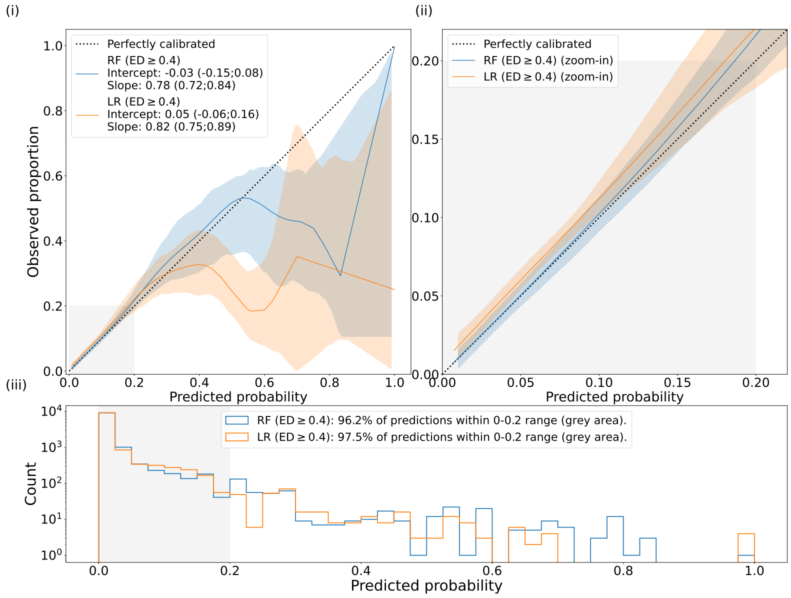

(d) ED ≥ 0.4

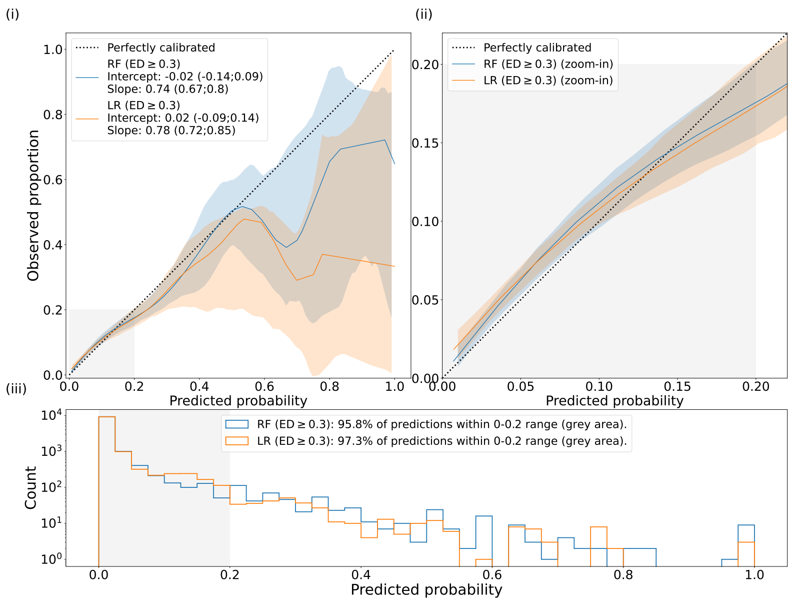

(e) ED $\geq$ 0.3

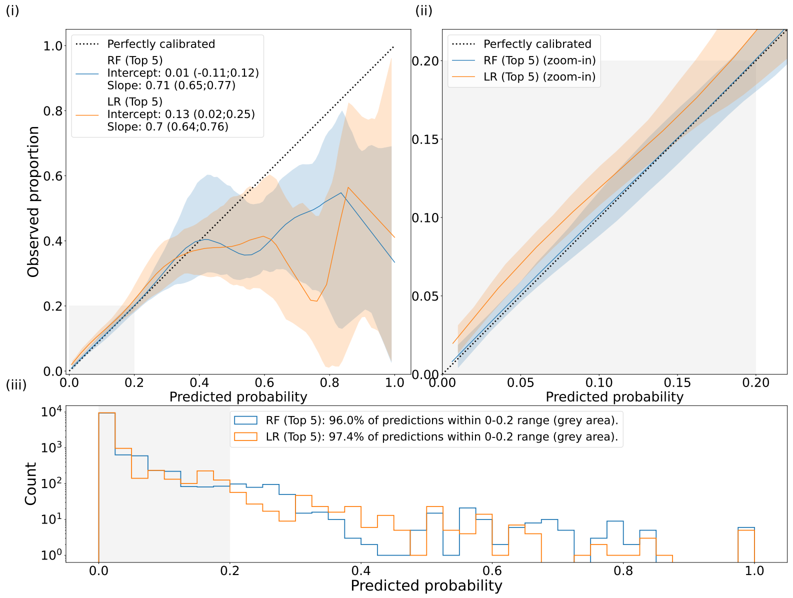

(f) Top 5

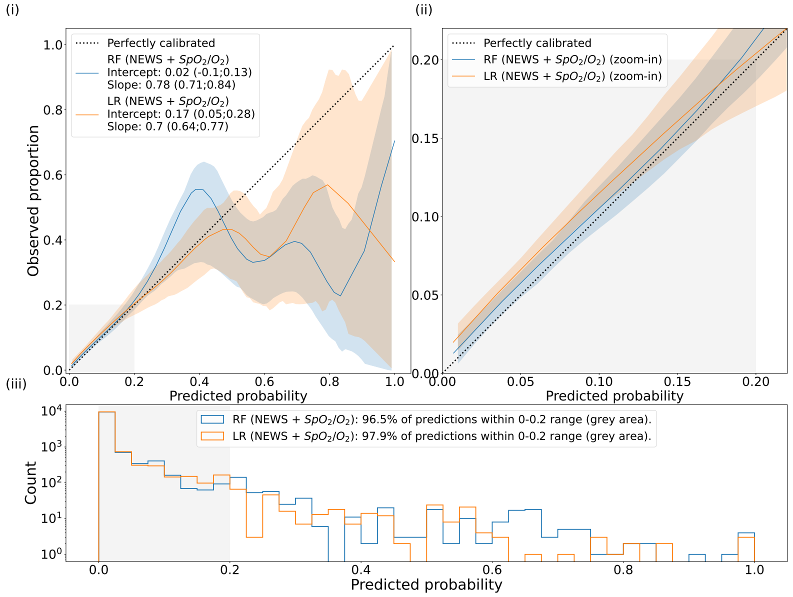

(g) NEWS + SpO_2/O_2

Figure 31: Hospital-specific loess smoothed flexible calibration curves yielded by the LR and RF models fitted with the different predictor sets: ED $\geq$ 0.5 (main text), ED $\geq$ 0.4, ED $\geq$ 0.3, MEWS, NEWS and Top 5. Shaded areas around the curves represent the 95% CIs.)
RF=random forest, LR=logistic regression, MEWS=Modified Early Warning Score, NEWS=National Early Warning Score

Appendix I.3: Influence of imputation on model performance

To examine the influence of imputation on the model performance, we repeated the temporal validation with 50 imputation rounds. That is, in each round, the missing values are imputed by random sampling from the posterior distributions with a different random seed. The resulting model performance in terms of discrimination (pAUCs) and calibration (calibration intercepts and slopes) over the 50 repetitions are visualized as boxplots in figure 32.

All the performance metrics show very little spread, only the spread of the calibration intercepts of the RF models is relatively large. The red stars represent the performances yielded by the models with a single imputation round (imputing values with the highest probability density), as reported in the main text.

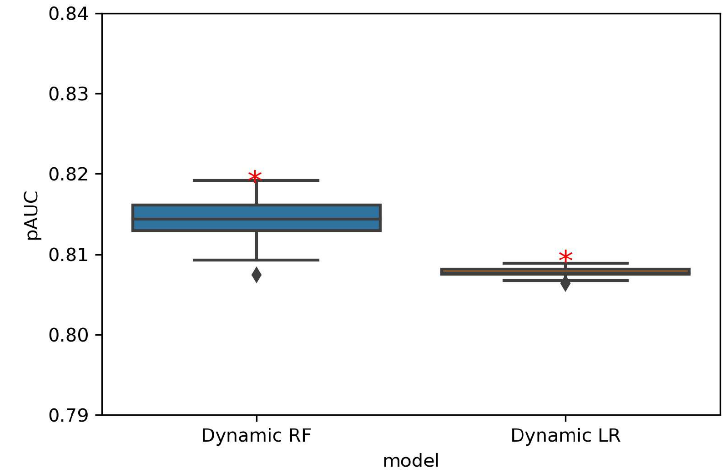

(a) pAUC

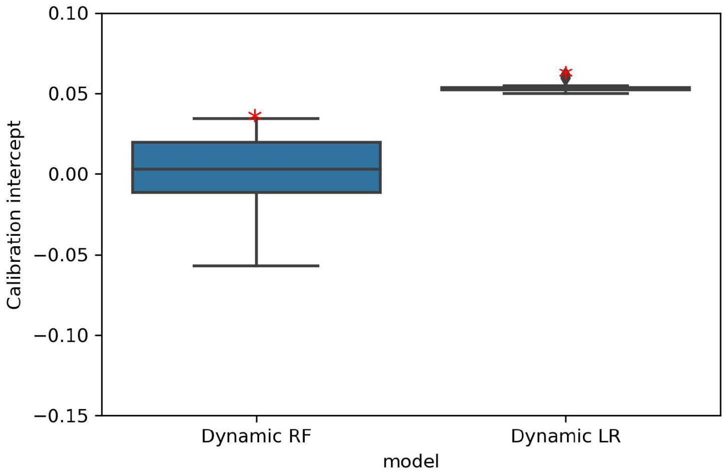

(b) Calibration intercept

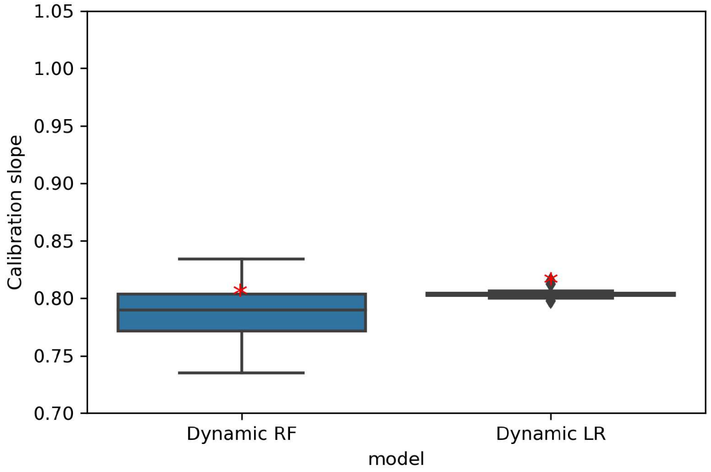

(c) Calibration slope

Figure 32: Boxplots visualizing the distributions of the point estimates of different performance metrics resulting from 50 imputation rounds. Red stars represent the point estimates resulting from the single imputation round presented in the main text.
pAUC = partial area under the receiver operating characteristic curve, RF=random forest, LR=logistic regression.

References

[1] Knight, S. R. *et al.* Risk stratification of patients admitted to hospital with covid-19 using the ISARIC WHO Clinical Characterisation Protocol: development and validation of the 4C Mortality Score. *BMJ* **370** (2020). URL `https://www.bmj.com/content/370/bmj.m3339`.

[2] Romanelli, D. & Farrell, M. W. AVPU Score. (2021).

[3] Pedregosa, F. *et al.* Scikit-learn: Machine learning in python. *Journal of machine learning research* **12**, 2825–2830 (2011).

[4] van Buuren, S. & Groothuis-Oudshoorn, K. mice: Multivariate Imputation by Chained Equations in R. *Journal of Statistical Software* **45**, 1–67 (2011). URL `https://www.jstatsoft.org/index.php/jss/article/view/v045i03`.

[5] Niculescu-Mizil, A. & Caruana, R. Predicting good probabilities with supervised learning. *Proceedings of the 22nd international conference on Machine learning* (2005).

[6] Knight, S. R., Ho, A. & Pius, R. Risk stratification of patients admitted to hospital with covid-19 using the isaric who clinical characterisation protocol: development and validation of the 4c mortality score. *BMJ* **2** (2020).

[7] Smith, G. B., Prytherch, D. R., Meredith, P., Schmidt, P. E. & Featherstone, P. I. The ability of the National Early Warning Score (NEWS) to discriminate patients at risk of early cardiac arrest, unanticipated intensive care unit admission, and death. *Resuscitation* **84**, 465–470 (2013).

[8] Xie, G. *et al.* The role of peripheral blood eosinophil counts in COVID-19 patients. *Allergy: European Journal of Allergy and Clinical Immunology* **76**, 471–482 (2021).

[9] Linssen, J. *et al.* A novel haemocytometric COVID-19 prognostic score developed and validated in an observational multicentre European hospital-based study. *eLife* **9**, 1–28 (2020).

[10] Liu, F. *et al.* Since January 2020 Elsevier has created a COVID-19 resource centre with free information in English and Mandarin on the novel coronavirus COVID- 19 . The COVID-19 resource centre is hosted on Elsevier Connect , the company ' s public news and information (2020).

[11] Foy, B. H. *et al.* Association of Red Blood Cell Distribution Width With Mortality Risk in Hospitalized Adults With SARS-CoV-2 Infection. *JAMA network open* **3**, e2022058 (2020).

[12] Yu, F. *et al.* Quantitative Detection and Viral Load Analysis of SARS-CoV-2 in Infected Patients. *Clinical infectious diseases : an official publication of the Infectious Diseases Society of America* **71**, 793–798 (2020).

[13] Coomes, E. A. & Haghbayan, H. Interleukin-6 in Covid-19: A systematic review and meta-analysis. *Reviews in Medical Virology* **30**, 1–9 (2020).

[14] Dahan, S. M. Ferritin as a Marker of Severity in COVID-19 Patients: A Fatal Correlation. *Orphanet Journal of Rare Diseases* **21**, 1–9 (2020). `arXiv:1011.1669v3`.

[15] McClish, D. K. Analyzing a portion of the ROC curve. *Medical decision making : an international journal of the Society for Medical Decision Making* **9**, 190–195 (1989).

[16] Qin, G. & Hotilovac, L. Comparison of non-parametric confidence intervals for the area under the ROC curve of a continuous-scale diagnostic test. *Statistical Methods in Medical Research* **17**, 207–221 (2008).

[17] Boyd, K., Eng, K. H. & Page, C. D. Area under the precision-recall curve: Point estimates and confidence intervals. In *Machine Learning and Knowledge Discovery in Databases. ECML PKDD 2013. Lecture Notes in Computer Science*, vol. 8190 (Springer, Berlin, Heidelberg, 2013).

[18] Vickers, A. J., van Calster, B. & Steyerberg, E. W. A simple, step-by-step guide to interpreting decision curve analysis. *Diagnostic and Prognostic Research* **3**, 18 (2019). URL `https://doi.org/10.1186/s41512-019-0064-7`.

[19] van Calster, B. *et al.* A calibration hierarchy for risk models was defined: from utopia to empirical data. *Journal of clinical epidemiology* **74**, 167–76 (2016).

[20] Cox, D. R. Two further applications of a model for binary regression. *Miscellanea* 562–565 (1958).

[21] Niculescu-Mizil, A. & Caruana, R. Predicting good probabilities with supervised learning. In *Proceedings of the 22nd International Conference on Machine Learning*, ICML '05, 625–632 (Association for Computing Machinery, New York, NY, USA, 2005). URL `https://doi.org/10.1145/1102351.1102430`.
